# The calcified eggshell matrix proteome of a songbird, the zebra finch (*Taeniopygia guttata*)

**DOI:** 10.1186/s12953-015-0086-1

**Published:** 2015-12-01

**Authors:** Karlheinz Mann

**Affiliations:** Max-Planck-Institut für Biochemie, Abteilung Proteomics und Signaltransduktion, D-82152 Martinsried, Am Klopferspitz 18 Germany

## Abstract

**Background:**

The proteins of avian eggshell organic matrices are thought to control the mineralization of the eggshell in the shell gland (uterus). Proteomic analysis of such matrices identified many candidates for such a role. However, all matrices analyzed to date come from species of one avian family, the Phasianidae. To analyze the conservation of such proteins throughout the entire class Aves and to possibly identify a common protein toolkit enabling eggshell mineralization, it is important to analyze eggshell matrices from other avian families. Because mass spectrometry-based in-depth proteomic analysis still depends on sequence databases as comprehensive and accurate as possible, the obvious choice for a first such comparative study was the eggshell matrix of zebra finch, the genome sequence of which is the only songbird genome published to date.

**Results:**

The zebra finch eggshell matrix comprised 475 accepted protein identifications. Most of these proteins (84 %) were previously identified in species of the Phasianidae family (chicken, turkey, quail). This also included most of the so-called eggshell-specific proteins, the ovocleidins and ovocalyxins. Ovocleidin-116 was the second most abundant protein in the zebra finch eggshell matrix. Major proteins also included ovocalyxin-32 and -36. The sequence of ovocleidin-17 was not contained in the sequence database, but a presumptive homolog was tentatively identified by N-terminal sequence analysis of a prominent 17 kDa band. The major proteins also included three proteins similar to ovalbumin, the most abundant of which was identified as ovalbumin with the aid of two characteristic phosphorylation sites. Several other proteins identified in Phasianidae eggshell matrices were not identified. When the zebra finch sequence database contained a sequence similar to a missing phasianid protein it may be assumed that the protein is missing from the matrix. This applied to ovocalyxin-21/gastrokine-1, a major protein of the chicken eggshell matrix, to EDIL3 and to lactadherin. In other cases failure to identify a particular protein may be due to the absence of this protein from the sequence database, highlighting the importance of better, more comprehensive sequence databases.

**Conclusions:**

The results indicate that ovocleidin-116, ovocleidin-17, ovocalyxin-36 and ovocalyxin-32 may be universal avian eggshell-mineralizing proteins. All the more important it is to elucidate the role of these proteins at the molecular level. This cannot be achieved by proteomic studies but will need application of other methods, such as atomic force microscopy or gene knockouts. However, it will also be important to analyze more eggshell matrices of different avian families to unequivocally identify other mineralization toolkit proteins apart from ovocleidins and ovocalyxins. Progress in this respect will depend critically on the availability of more, and more comprehensive, sequence databases. The development of faster and cheaper nucleotide sequencing methods has considerably accelerated genome and transcriptome sequencing, but this seems to concur with frequent publication of incomplete and fragmented sequence databases.

**Electronic supplementary material:**

The online version of this article (doi:10.1186/s12953-015-0086-1) contains supplementary material, which is available to authorized users.

## Background

The avian eggshell consists of several layers, the innermost eggshell membranes, the calcified layer, and the outermost cuticle [1, 2]. Its biological function is to protect the developing embryo against physical impact while at the same time enabling gas exchange through pores pervading the entire calcified layer. It also protects the egg against microbial invasion and provides calcium to the growing embryo by partial solubilization of the calcified layer. The calcified layer consists to more than 95 % of calcium carbonate in the form of calcite and can be divided into three electron microscopically discernible compartments. The innermost mammillary cone layer makes contact to the underlying eggshell membranes, and the flat surface of individual cones forms the basis for the calcite columns of the palisade layer. Finally, a vertical crystal layer separates palisades from cuticle. Formation of the calcified layer starts in the red isthmus by deposition of nucleating matrix components on the outer eggshell membrane. Starting from these regularly spaced sites, bulk mineralization takes place in the shell gland (uterus) with a transient amorphous calcium carbonate phase preceding calcite crystal formation [3]. Mineralization is thought to be controlled by proteins and proteoglycans that form approximately 2 % of the mature calcite layer [2, 4–9], and much effort has been invested in identifying the responsible components.

Most research on eggshell proteins was performed with chicken eggshell, probably because of the commercial importance and easy availability. Among the first eggshell matrix proteins isolated and characterized were several previously known abundant egg white components, such as ovalbumin [10], lysozyme [11], and ovotransferrin [12]. Egg white proteins are predominantly produced and secreted by the magnum section of the oviduct and may reach the shell gland together with the unfinished egg that is driven by peristaltic oviduct wall movements towards the uterus. However, because lysozyme [11], ovotransferrin [12] and ovalbumin [13] messages were also identified at a much lower level (compared to magnum) in white isthmus and in traces in red isthmus and uterus, it is possible that a presently unknown share of these proteins is produced and secreted in other oviduct sections than magnum. Immunochemical evidence indicated that these proteins were not surface contaminants, but were located within calcified structures.

A potentially much more interesting group of proteins are the so-called eggshell- specific proteins. This label was coined because these proteins were originally found to be produced and secreted by uterus epithelial cells, but not in other oviduct sections and not in a few selected other body tissues. Although it is now known that not all of them are strictly eggshell-specific, this generic term is generally retained. The first of these proteins that was detected and characterized was ovocleidin-17 [14], a C-type lectin-like protein [15] that was reported to influence in vitro calcite formation [16, 17] and to have antimicrobial activity [18]. Next, ovocleidin-116 was cloned and localized by ultrastructural colloidal gold to matrix vesicles throughout the palisade layer and the calcium reserve assembly of the mammillary layer [19]. OC116 was suggested to have a mammalian counterpart, matrix extracellular phosphoprotein (MEPE), belonging to the secretory calcium-binding phosphoprotein (SCPP) group of proteins [20], or small integrin-binding ligand N-linked glycoproteins (SIBLING), a group of proteins including dentin matrix protein, osteopontin, dentin sialoprotein and other proteins important for skeletal and dental mineralization and remodeling [21, 22]. Ovocalyxin-36 is an eggshell-specific protein belonging to the family of antimicrobial lipopolysaccharide-binding/bactericidal permeability-increasing/PLUNC proteins and was located throughout the eggshell including the membranes [23, 24]. It is reported to bind bacterial lipopolysaccharide [25] and to modulate the production of proinflammatory mediators [26], but a possible direct role in eggshell mineralization is unknown at present. Ovocalyxin-32 is a member of the latexin family of carboxypeptidase inhibitors and was located in palisade layer, vertical crystal layer and cuticle [27]. Its function is unknown at present. Other eggshell-specific proteins frequently mentioned in chicken eggshell matrix publications are ovocalyxin-25, a poorly characterized protein containing a protease inhibitor domain, and ovocalyxin-21, which is apparently identical to the gastric secretome component gastrokine-2 [8, 28].

Proteomic mass spectrometry-based high-throughput analysis depends on the availability of comprehensive sequence databases provided by genome sequences or transcriptomes. The era of proteomic analysis of avian eggshells started shortly after the publication of the first avian genome sequence, which was that of chicken [29]. The first studies of this type described the analysis of the proteome and phosphoproteome of the chicken calcified layer acid-soluble matrix [30, 31]. This was followed by proteomic analysis of other eggshell compartments such as the acid-insoluble matrix [32, 33], the eggshell cuticle [34, 35], the soluble fraction of the eggshell membranes in conjunction with the innermost eggshell calcified layer (mammillary cones) [36], and eggshell membranes alone at different stages of chick embryonal development [37]. In addition, the proteome of the uterus fluid bathing the egg during shell mineralization was compared to the proteome of the calcified shell [38] and uterus fluid proteomes at different stages of mineralization were compared to each other [39]. Finally, the eggshell itself was analyzed at different stages of mineralization [40]. The sum of different proteins identified in these studies was counted to be 675 [39] and the sum of reports yielded important information on the distribution of shell proteins in different shell compartments and the temporal sequence of their appearance in the uterus fluid. Proteomic studies were complemented by transcriptomic studies to track expression of proteins with possible importance for mineralization [41–46].

Much less is known about the eggshells of other species. For instance, organic matrix composition of various species was explored by immune-blotting using antibodies against major chicken eggshell proteins [47], the involvement of amorphous calcium carbonate in quail eggshell formation was reported along with identification of ovomucoid and lysozyme as matrix components [48], and a preliminary quail eggshell matrix proteome was published using chicken sequences to identify quail proteins [49]. More recently the publication of genome sequence databases of turkey [50] and quail [51] enabled in-depth proteomic analysis of turkey [52] and quail [53] calcified eggshell proteomes. The number of identified mineralized eggshell proteins of chicken, turkey and quail was 675, 697 and 622, respectively, with an overlap of 311 proteins. Common major proteins also included the so-called eggshell - specific proteins ovocleidin-116 and ovocalyxin-36. Other proteins of this group, such as ovocleidin-17 and ovocalyxin-32 were not contained in all databases and at best tentatively identified in proteomes by circumstantial evidence. All three species analyzed so far are relatively closely related and belong to one avian family, the Phasianidae. Therefore it seemed to be interesting to compare these eggshell proteomes to that of a bird not belonging to this family and thus to analyze the distribution of presumptive biomineralizing proteins between lineages. Because massspectrometry-based high-throughput proteomics still depends on comprehensive sequence databases, the obvious choice for such a comparison was zebra finch, a songbird of the Estrididae family, the genome sequence of which was published in 2010 [54]. Chicken and zebra finch lineages were estimated to have separated 90–100 million years ago, near the base of avian radiation [55, 56].

## Materials and methods

Zebra finch eggs were from birds raised in aviaries of the Max Planck Institute for Ornithology in Seewiesen, Germany [57]. Eggs were cleaned superficially, dried, cracked and emptied. The shells were washed under de-ionized water, dried and transported on dry ice. Eggshell pieces were cleaned in 14 % sodium hypochlorite solution (14 % active Cl_2_; GPR Rectapur, VWR Chemicals, Germany; 10 ml/g) for 2 h at room temperature starting with 5 min sonication to facilitate wetting of surfaces. Clean eggshell pieces were washed with de-ionized water, dried, and stored at −20° until decalcification. Three pools of 20 eggshells were used as biological replicates and each pool was analyzed with 10 technical replicates. Shell samples (approximately 1.2 g per replicate) were decalcified with 40 ml of 10 % acetic acid for 14 h at 4 °C. The resulting clear solution was dialyzed (Spectra/Por 6 dialysis membrane, molecular weight cut-off 2000; Spectrum Europe, Breda, The Netherlands) successively against 3 × 1 l of 10 % acetic acid and 3 × 1 l of 5 % acetic acid at 4–6 °C and then lyophilized.

Reduction, carbamidomethylation and enzymatic cleavage of matrix proteins were performed using a modification of the FASP (Filter-aided sample preparation) method [58] as outlined below. Aliquots of 200 μg of matrix were suspended in 200 μl of 0.1 M Tris, pH 8, containing 6 M guanidine hydrochloride and 0.01 M dithiothreitol (DTT). This mixture was heated to 56 °C for 60 min, cooled to room temperature, and centrifuged at 14,000x g in an Eppendorf bench-top centrifuge 5415D for 15 min. The supernatant was loaded into an Amicon Ultra 0.5 ml 30 K filter device (Millipore; Tullagreen, Ireland). DTT was removed by centrifugation at 14,000x g for 15 min and washing with 2 × 1 volume of the same buffer. Carbamidomethylation was done in the device using 0.1 M Tris buffer, pH 8, containing 6 Mguanidine hydrochloride and 0.05 mM iodoacetamide and incubation for 45 min in the dark. Carbamidomethylated proteins were washed with 0.05 M ammonium hydrogen carbonate buffer, pH 8, containing 2 M urea, and centrifugation as before. Trypsin (2 μg, Sequencing grade, modified; Promega, Madison, USA) was added in 40 μL of 0.05 M ammonium hydrogen carbonate buffer containing 2 M urea and the devices were incubated at 37 °C for 16 h. Peptides were collected by centrifugation and the filters were washed twice with 40 μL of 0.05 M ammonium hydrogen carbonate buffer and twice with 1 % trifluoroacetic acid in 5 % acetonitrile. The acidic peptide solution (pH 1–2) was applied to C18 Stage Tips [59] and the eluted peptides were vacuum-dried in an Eppendorf concentrator.

Peptide mixtures were analysed by on-line nanoflow liquid chromatography using the EASY-nLC 1000 system (Proxeon Biosystems, Odense, Denmark, now part of Thermo Fisher Scientific) with 20 cm (replicates Z1 and Z3) or 50 cm (replicates Z2 and Z3b; Z3b was the same as Z3, run again with the longer column) capillary columns of an internal diameter of 75 μM filled with 1.8 μM Reprosil-Pur C18-AQ resin (Dr. Maisch GmbH, Ammerbuch-Entringen, Germany). Peptides were eluted with a linear gradient from 2–5 % buffer B (80 % acetonitrile in 0.1 % formic acid) in 5 min, 5–30 % B in 90 min, 30–60 % B in 5 min and 60–95 % in 5 min at a flow rate of 250 nl/min and a temperature of 50 °C. The eluate was electro-sprayed into an Orbitrap Q Exactive Plus (Thermo Fisher Scientific, Bremen, Germany) using a Proxeon nanoelectrospray ion source. The instrument was operated in a HCD top 10 mode essentially as described [60]. The resolution was 70,000 for full scans and 17,500 for fragments (both specified at m/z 200). Ion target values were 1e6 and 5e4ms, respectively. Dynamic exclusion time was 20 s. MS runs were monitored using the SprayQc quality monitoring system [61]. Raw files were processed using the Andromeda search engine-based version 1.5.1.6 of MaxQuant (http://www.biochem.mpg.de/5111795/maxquant) with enabled second peptide, iBAQ, and match between runs (match time window 0.5 min; alignment time window 20 min) options [62–64]. The sequence databases used were a *Taeniopygia guttata* subset of UniProt (release 2014_02, 19725 entries; http://www.ebi.ac.uk/ebisearch/search.ebi?db=proteinSequences&t=taeniopygia+guttata) and a *Taeniopygia guttata* subset of NCBI protein database (release 68, 23238 entries; http://www.ncbi.nlm.nih.gov/protein?term=%22Taeniopygia%20guttata%22%5BOrganism%5D%20&cmd=DetailsSearch), combined with the reversed sequences for FDR calculation and sequences of common contaminants, such as human keratins and mammalian cytoskeletal proteins. Carbamidomethylation was set as fixed modification. Variable modifications were oxidation (M), N-acetyl (protein), pyro-Glu/Gln (N-term), phospho (STY), and hydroxyproline. The first search peptide tolerance was set to 20 ppm, the main search peptide tolerance was set to 4.5 ppm. Two missed cleavages were allowed and the minimal length required for a peptide was seven amino acids. Maximal FDR for peptide spectral match, proteins and site was set to 0.01. The minimal score for modified and unmodified peptides was 60. Identifications with only two sequence-unique peptides were routinely validated using the MaxQuant Expert System software of MaxQuant [65] considering the assignment of major peaks, occurrence of uninterrupted y- or b-ion series of at least four consecutive amino acids, preferred cleavages N-terminal to proline bonds, the possible presence of a2/b2 ion pairs, immonium ions and mass accuracy. Fragment peaks of phosphopeptides without MaxQuant standard annotation were annotated manually by comparing masses of predicted fragments (ProteinProspector v5.14.1; http://prospector.ucsf.edu/prospector/cgi-bin/msform.cgi?form=msproduct) to masses in corresponding spectra of raw-files. Only proteins identified in at least two replicate sets were accepted. The iBAQ (intensity-based absolute quantification) [66] option of MaxQuant was used to calculate, based on the sum of peak intensities, the approximate share of each protein in the total proteome, including identifications that were not accepted. This enabled us to discern between minor and major proteins.

Sequence database searches were performed with FASTA (http://www.ebi.ac.uk/Tools/sss/fasta/) [67] against current releases of Uniprot Knowledgebase (UniProtKB) or with NCBI BLASTp (http://blast.ncbi.nlm.nih.gov/Blast.cgi) [68] against current releases of the non-redundant NCBI protein sequence database. The quail sequence database (predicted gene database of *Coturnix japonica* [51]; http://www.nodai-genome.org/japanese_quail.html?lang=en; 30810 entries; downloaded November 2014) was searched using the Local Blast function [69] of BioEdit Sequence Alignment Editor version 7.2.5 from http://www.mbio.ncsu.edu/bioedit/bioedit.html. Other bioinformatics tools used were Kalign (http://www.ebi.ac.uk/Tools/msa/kalign/) [67] and Clustal Omega for sequence alignments (http://www.ebi.ac.uk/Tools/msa/clustalo/) [70], InterPro (http://www.ebi.ac.uk/interpro/) [71] for domain predictions, big-PI Predictor [72] (http://mendel.imp.ac.at/gpi/cgi-bin/gpi_pred.cgi) for prediction of GPI attachment sites, and secretion signal sequences were predicted with SignalP 4.1 (http://www.cbs.dtu.dk/services/SignalP/) [73]. Phosphorylation sites were predicted with NetPhos 2.0 (http://www.cbs.dtu.dk/services/NetPhos/) [74]. Kinase motifs apart from FAM20C consensus phosphorylation motifs were predicted using NetPhosK (http://www.cbs.dtu.dk/services/NetPhosK/) [75] and PhosphoMotif finder (http://www.hprd.org/PhosphoMotif_finder) [76].

SDS-PAGE was done using pre-cast 4-12 % Novex Bis-Tris gels in the MES buffer system using reagents and protocols supplied by the manufacturer (Invitrogen, Carlsbad, CA). The kit sample buffer was modified by adding β-mercaptoethanol to a final concentration of 1 %, and samples were suspended in 25 μl sample buffer/75 μg of organic matrix and heated to 70 °C for 10 min. Samples were centrifuged for 5 min at 14,000x g to sediment PAGE sample buffer-insoluble material. N-terminal sequencing on a Procise 492 cLC (Applied Biosystems) of a 17 kDa protein band blotted on PVDF (Immobilon P, Applied Biosystems) and in-gel cleavage of this protein with trypsin to possibly identify internal peptides were performed following established protocols (J. Kellermann and R. Mentele, core facility of the MPIB). The peptide mixture was mixed with alpha-cyano-4-hydroxy-cinnamic acid as a matrix. MALDI-MS on a 4800 AB Sciex MALDI-TOF/TOF was used to identify peptides suitable for MS/MS sequencing. MS/MS was performed in the same instrument with a 355 nm Nb-YAG laser in positive reflector mode at 20 kV acceleration voltage. Results were evaluated with the Mascot program package (Matrix Science Ltd, London, England; http://www.matrixscience.com/server.html). The peptide mass tolerance was 100 ppm, the MS/MS tolerance was 0.25 Da. Carbamidomethylation was set as fixed modification and the variable modifications were methionine oxidation and tryptophane oxidation. One miss-cleavage was allowed, and the score threshold was set to *p* < 0.05.

## Results and discussion

**Fig. 1:**
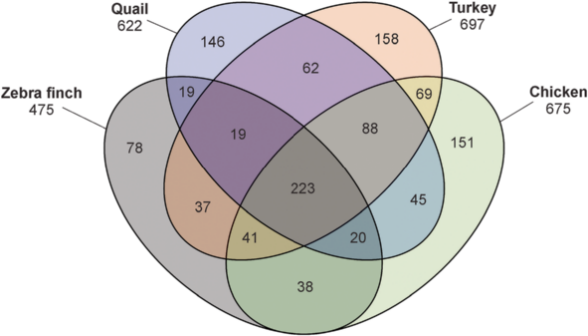
Four-ellipse Venn diagram comparing zebra finch, quail, turkey and chicken calcified eggshell proteomes. The four-ellipse template was taken from https://commons.wikimedia.org/wiki/File:Venn%27s_four_ellipse_construction.png

Compared to chicken, turkey or quail eggshells zebra finch eggshells are extremely thin and fragile. Therefore our usual method to remove cuticle and eggshell membranes mechanically after weakening attachment of these shell compartments to the calcified layer with a short incubation of shell pieces in EDTA was not applicable. This was replaced by incubating in sodium hypochlorite solution, a method commonly used to clean invertebrate skeletal elements from any organic material attached to surfaces, but also applied successfully to chicken eggshells previously [77]. The yield of organic matrix from three different preparations was approximately 10 mg/g of cleaned eggshell. Analysis of zebra finch eggshell matrix yielded 792 preliminary identifications (Additional file 1: ProteinGroups) with 5259 sequence-unique peptides (Additional file 2: Peptides). Elimination of identifications with only one peptide or present only in one replicate group, and the grouping together of obvious fragments of identical proteins, resulted in 475 accepted identifications (Additional file 3: Accepted protein identifications). Most of these proteins/protein groups were previously identified in either chicken, turkey or quail eggshell proteomes. Only 78 proteins were new in zebra finch eggshell proteomes (Fig. 1). Approximate protein quantitation based on iBAQ intensities indicated that 16 proteins with a percentage of ≥1.0 already covered 73 % of the total identified shell proteome (Table 1) and 75 proteins with >0.1 % summed up to 90 % of the total. In the following section I will discuss what I believe to be the most interesting of them, with special attention to the so-called eggshell-specific proteins.

**Table 1:** Major proteins (>0.1 %) of the zebra finch eggshell calcified layer

Protein	Accession	iBAQ %	iBAQ % in turkey ^1^ /quail ^2^ eggshell	Abundance in chicken eggshell ^3^ (emPAI)
Ovalbumin(−like)	gi|224045100	35.20	12.52/15.97	High (65.3)^**3a**^
Similar to ovocleidin-116	gi|224049274/H0YVI0	8.37	31.30/46.40	High (65.3)^**3a**^
Ovalbumin-related protein Y-like	gi|224045098	5.60	0.43/0.21	Intermediate (3.8)^**3a**^
Ovalbumin-like (OVALX?)	gi|224045096/H0YV21	4.08	<0.01/0.21	Intermediate (3.8)^**3a**^
BPIL1/Tenp-like	H0Z7I9/gi|449486399	3.72	0.01/-	Low (0.6)^**3a**^
Similar to serum albumin (ALB)	gi|449499399/H0YTL4	2.93	2.57/0.39	High (113.5)^**3a**^
Similar to ovomucoid	H0YQL9/gi|224067572	1.73	0.05/0.11	Intermediate (2.2)^**3a**^
	gi|449512555/H1A0C8			
Similar to Pigment epithelium-derived factor (PEDF)	H0Z5S2/gi|449479753	1.62	0.64/0.31	Intermediate (6.0)^**3a**^
Similar to BPI fold-containing family B member 4/Ovocalyxin-36	gi|224078167/H0Z7K8	1.59	6.00/7.80	High (23.2)^**3a**^
Insulin-like growth factor-binding protein 5 (IGFBP5)	H0Z3W8/gi|449506910	1.26	-/0.13	Low (0.6)^**3a**^
Similar to vitellogenin-2 (VIT2)	gi|224057610/H0Z847	1.22	0.02/<0.01	Low (1.7)^**3a**^
Similar to ovostatin (A2M-1)	H0ZS62	1.20	0.05/0.08	Low (1.5)^**3a**^
Similar to vitellogenin-1 (TPRXL)	H0Z8U7/gi|224057648	1.08	0.01/0.01	Low (0.8)^**3a**^
Glutathione peroxidase 3 (GPX3)	gi|401664564/H0YRF2	1.06	0.04/0.36	Intermediate (2.2)^**3a**^
Ubiquitin (C)	B5G2Q3/gi|5822013	1.06	0.12/0.12	High (9.0)^**3a**^
Actin(s)	H0Z0Q3/gi|224061779	1.05	-/0.36	High (9.0)^**3a**^
Putative apolipoprotein A-I	B5G356/gi|350538495	0.98	0.05/0.17	Intermediate (2.2)^**3a**^
Similar to mesothelin	gi|449476510/H0ZYM3	0.91	<0.01/<0.01	Low (0.4)^**3a**^
Similar to Nucleobindin-2 (NUCB2)	H0ZEB8/gi|449501999	0.76	0.23/0.07	Intermediate (7.3)^**3a**^
Similar to leukocyte antigen 86 (LY86)	H0YVQ1/gi|224045162	0.72	<0.01/<0.01	Intermediate (0.3)^**3a**^
Similar to ovotransferrin (LTF)	H0Z885/gi|449509524	0.69	2.22/0.19	High (22.9)^**3a**^
Similar to Sulfhydryl oxidase 1 (QSOX1)	H1A439/gi|449509315	0.56	1.08/0.18	Intermediate (4.7)^**3a**^
Similar to Mucin-5 AC	gi|449504198/H0ZGZ6/	0.54	0.15/0.35	Low (1.4)^**3a**^
	H0ZGY9/H0ZGW8			
Similar to plasminogen (LPA-2)	gi|224048112/H0ZML2	0.53	<0.01/-	-
Similar to ovoinhibitor	gi|449474881/H0YQL7	0.53	0.01/0.01	High (12.1)^**3a**^
Similar to cathepsin D (CTSD)	H0ZGM2/gi|224050910	0.50	0.22/0.18	Intermediate (5.3)^**3a**^
Similar to multiple inositol polyphosphate phosphatase 1 (MINPP1)	gi|224052234/H0Z3R0	0.43	<0.01/<0.01	Low (2.1)^**3a**^
Similar to ovocalyxin-32 (RARRES1)	H0ZLS5/gi|449510033	0.40	−/−	High (71.0)^**3a**^
Similar to Angiopoietin-related protein 3 (ANGPTL3)	gi|224058351/H0ZHW1	0.39	−/−	Low (2.0)^**3a**^
Fibronectin type III domain-containing protein 1 (FNDC1)	gi|449497556/H0ZMA1	0.39	−/−	Intermediate^**3b**^
Similar to tumor necrosis factor receptor superfamily member 6B (TNFRSF6B)	gi|224078299/H0Z9P3	0.38	0.01/0.01	Intermediate^**3b**^
Calcitonin gene-related peptide 2 (CALCB)/ procalcitonin	H0ZDU9/gi|224050809	0.37	-/0.01	-
Similar to N-acetyl-glucosamine-6-sulfatase (GNS)	H0Z834/gi|449481578	0.36	0.04/0.03	Low (0.8)^**3a**^
Similar to Tetraspanin (TSPAN1)	gi|449508571/H0ZCW3	0.33	−/−	-
Similar to carbonic anhydrase 4(CA4)	H0ZCC0/gi|449480381	0.32	0.35/0.03	Intermediate (2.9)^**3a**^
Similar to avidin	H0YUD8/gi|224090240	0.30	2.67/1.95	Intermediate (2.2)^**3a**^
Similar to Lysyl oxidase 2 (LOXL2)	H1A3U9/gi|449488345	0.30	0.01/0.04	Low (0.2)^**3a**^
Prostatic acid phosphatase-like; domain: Histidine phosphatase superfamily	gi|449493197	0.29	−/−	Low (1.6)^**3a**^
Similar to alpha-2-HS-glycoprotein (AHSG)	gi|449509821/H0ZJJ6	0.29	0.01/-	Low (1.3)^**3a**^
Similar to lactadherin (MFGE8)	H0ZD74/gi|449471648	0.27	1.25/0.36	High (15.9)^**3a**^
Similar to stanniocalcin-1(STC1)	H0Z285/gi|449488187	0.27	0.01/<0.01	Low (0.5)^**3a**^
Similar to 45 kDa calcium-binding protein (SDF4)	H0Z0U7/gi|224080053	0.26	0.09/0.03	Intermediate (2.9)^**3a**^
Similar to ovostatin	gi|449485099/H1A5U5	0.23	0.05/0.34	Low (1.5)^**3a**^
Putative cystatin	B5G368/gi|197129295	0.22	1.67/0.11	High (45.4)^**3a**^
Similar to heat shock cognate 71 kDa protein (HSPA8)	gi|224083318/H0YQE7	0.22	0.01/0.01	Low (1.23)^**3a**^
Putative peroxiredoxin 1	B5G0M2/gi|197128341	0.21	0.01/0.02	Low (1.0)^**3a**^
Semaphorin-3 F	gi|224066001/H0Z7X2	0.21	<0.01/-	-
Similar to epididymis-specific alpha-mannosidase (MAN2B2)	H0ZIJ5/gi|449501297	0.21	0.06/0.02	Low (0.8)^**3a**^
Slit homolog 2 protein (SLIT2)	H0ZH52/gi|449500994	0.21	0.01/0.01	Low (0.6)^**3a**^
Similar to glia-derived nexin (SERPINE2)	gi|224060004/H0ZCA5	0.20	0.18/0.31	High (11.1)^**3a**^
Similar to proactivator polypeptide (PSAP-1)	H0Z157/gi|449504732	0.20	0.06/0.12	High (9.0)^**3a**^
Peptidyl-prolyl cis-trans isomerase B	B5G4N8/gi|350536587	0.19	-/0.06	High (13.7)^**3a**^
Similar to lysosomal Pro-X carboxypeptidase (PRCP)	gi|224043680/H0ZRS7	0.19	-/<0.01	-
Renin/prorenin receptor (ATP6AP2)	H0Z8C1/gi|449482762	0.19	0.05/0.03	Low (1.8)^**3a**^
Similar to cathepsin L1/L2 (CTSL1)	H0YQQ3/gi|449513868	0.18	0.02/0.02	Low (0.2)^**3a**^
Similar to apovitellenin-1 (APOVLDLII)	gi|224044051/H0ZT64	0.16	0.01/0.16	Low (1.5)^**3a**^
Alpha-enolase (ENO1)	H0Z0D8/gi|224079993	0.16	0.01/0.02	Low (1.1)^**3a**^
Similar to beta-hexosaminidase subunit beta (HEXB)	H0Z6E7/gi|224091413	0.16	0.03/<0.01	Low (0.6)^**3a**^
Similar to Netrin-3 (NTN3)	gi|449475443/H0YYI9	0.15	−/−	-
Similar to Carboxypeptidase (CTSA)	H0Z6V7/gi|449486379	0.15	0.08/0.02	Intermediate (2.4)^**3a**^
Histone H4	B5FXC8/gi|350536313	0.14	0.04/0.03	Low (1.2)^**3a**^
EGF-containing fibulin-like extracellular matrix protein 1 (EFEMP1)/Fibulin-5	H0Z947/gi|224047478	0.14	0.02/-	Low (0.2)^**3a**^
Ezrin (EZR)	H0ZLB9/gi|449497375	0.14	<0.01/0.02	Low (1.2)^**3a**^
Syntenin-1 (SDCBP)	H0ZLL3/gi|449494168	0.13	0.02/0.10	Intermediate (2.8)^**3a**^
Similar to hyaluronan and proteoglycan link protein 3 (HAPLN3)	H0ZD86/gi|449471652	0.13	0.05/0.07	Low (1.2)^**3a**^
Similar to angiotensinogen (AGT)	gi|224047800/H0ZI58	0.12	<0.01/0.01	Low (0.2)^**3a**^
Similar to vitamin K-dependent protein S (PROS1)	H0ZTE6/gi|449485775	0.12	0.03/-	Low (0.4)^**3a**^
L-lactate dehydrogenase	B5G2G3/gi|310703675	0.11	0.01/0.01	Low (0.2)^**3a**^
Similar to ceroid-lipofuscinosis neuronal protein 5 (CLN5)	H0ZQM7/gi|224043499	0.11	0.02/0.01	Low (0.6)^**3a**^
Similar to Riboflavin-binding protein	H0ZCK8/gi|224058069	0.11	0.02/0.01	Low (0.7)^**3a**^
Similar to Out at first protein homolog (OAF)	H0YQ63/gi|224083284	0.11	<0.01/0.03	Low (1.7)^**3a**^
Similar to alpha-2-antiplasmin	gi|449480130/H0Z5Q3	0.11	0.60/0.80	Intermediate^**3b**^
Similar to solute carrier family 2, facilitated glucose transporter member 3 (SLC2A3)	gi|449484853/H0ZSC5	0.11	-/0.01	Low (1.8)^**3a**^
Similar to nephronectin (NPNT)	H0Z095/gi|449500286	0.11	0.02/0.01	Intermediate (3.8)^**3a**^
Similar to alpha-2-macroglobulin-like 1	H0ZSA0	0.11	-/0.01	Low (0.2)^**3a**^

The complete list of accepted proteins/protein groups is shown in Supplementary file 3.^**1**^, data from [52];^**2**^, [53]; ^**3**^, estimate based on emPAI values (in brackets).^**3a**^, [30];^**3b**^, [38] (estimate based on peak intensities reported in this publication)

### Ovocleidins and ovocalyxins (so-called eggshell-specific proteins)

**Fig. 2:**
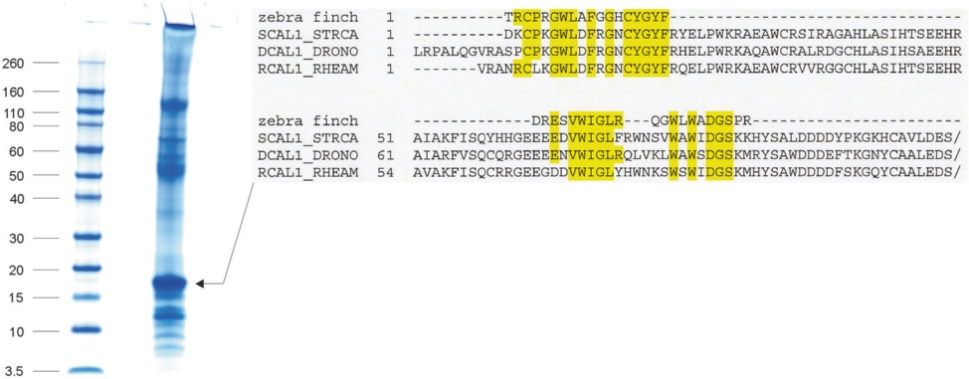
SDS-PAGE analysis of zebra finch eggshell matrix. Gel electrophoretic analysis of 75 μg of eggshell matrix applied in 25 μl sample buffer. The relative mobility of molecular weight markers is indicated in kDa. The arrow points to a prominent 17 kDa band that was examined by N-terminal and internal amino acid sequencing. The resulting sequences and their alignment to similar eggshell proteins are shown at right. Identical amino acids are highlighted

The most problematic of this group of proteins in terms of possible conservation between species is ovocleidin-17 (OC17). This C-type lectin-like protein is a major component of the chicken eggshell matrix [14, 15, 30]. It is contained in the chicken matrix at all stages of mineralization, but seems to be most abundant when amorphous calcium carbonate (ACC) is transformed into calcite aggregates [40]. Suggested functions are regulation of calcite crystallization [39] and anti-microbial activity [18]. The postulated direct function in mineralization is essentially based on in vitro calcium carbonate precipitation assays [16, 17, 78] and is supported by computer simulations [79–81]. The sequence of OC17 was not contained in the chicken genomic sequence database published in 2004 [29] and a full-length cDNA-derived sequence was published only very recently [82]. Before, the OC17 sequence in databases was that of the mature protein derived from Edman chemistry sequencing of the isolated protein [15]. Previous failure to detect OC17 mRNA is suggested to be related to the very high GC content (72.17 %) of the transcript [82]. The turkey and quail genes or mRNAs were apparently also not detected because no OC17 sequence was found in the corresponding sequence databases. Nevertheless, isolation and sequence analysis of very similar major eggshell matrix proteins in goose (ansocalcin [83], ostrich (struthiocalcins [84]), emu (dromaiocalcins [85]) and rhea (rheacalcins [85]) suggested widespread conservation of this protein. Furthermore, there is some circumstantial evidence for its presence in turkey [47, 52] and quail [49]. The zebra finch sequence database is apparently also missing a good candidate for an OC17 homolog. Protein XP_002189493/gi|449509191 suggested previously to be the zebra finch OC17 [8] is more similar to regenerating islet-derived protein 4 (REG4) than to known eggshell C-type lectin-like proteins. REG4 is a C-type lectin-like protein secreted by regenerating pancreas islet cells. This protein was identified among the major proteins of turkey and quail eggshell proteomes [52, 53], as a minor protein in chicken eggshell proteome [30], and also as a minor component of zebra finch eggshell matrix (Additional file 3). SDS polyacrylamide electrophoresis of zebra finch eggshell matrix (Fig. 2) showed a major protein band at a relative mobility of approximately 17 kDa, the expected migration distance of mature OC17. N-terminal sequencing of the blotted protein using Edman chemistry yielded the first 18 amino acids of this protein and sequence database searches with FASTA showed it to be a member of the C-type lectin-like family. There were three previously characterized eggshell C-type lectin-like proteins among the 10 best matches, with sequence identities of 70–75 % and FASTA e-values of 0.022–0.23 (Fig. 2). These three proteins were struthiocalcin-1 from ostrich [84], dromaiocalcin-1 from emu [85], and rheacalcin-1 from rhea [85]. No zebra finch protein was among the first 500 matches. In addition, the protein in this band was cleaved in-gel with trypsin and the eluted peptides were analyzed by MALDI-TOF/TOF MS/MS. The peptide sequences obtained were QGWLWADGSPR, DRESVWIGLR, and the shorter version of the latter, ESVWIGLR. The first peptide sequence matched completely to an uncharacterized protein of the Tibetan ground tit, XP_005534326 (aa567–577; e-value 0.008) in NCBI BLASTp, and was located to a predicted C-type lectin-like domain of this protein. The second peptide and its shorter version matched to many proteins with low confidence. However, all peptide sequences also aligned to the sequences of the ratite eggshell C-type lectin-like proteins (Fig. 2), yielding an overall identity of 60 %. Therefore I believe that these peptides may belong to the same protein as the N-terminal sequence. Staining intensity of this 17 kDa band indicated that this C-type lectin-like protein may be among the major zebra finch eggshell matrix proteins (Fig. 2). The sum of evidence suggested that this protein may be an unknown C-type lectin-like protein, possibly a close relative or even homolog, of chicken OC17.

Ovocleidin-116 (OC116) is a major protein in all eggshell matrices analyzed so far [30, 52, 53]. The zebra finch homolog was correctly predicted previously from published database sequences [8]. OC116 was the second most abundant among the proteins identified by LC-MS/MS in zebra finch eggshell matrix (Table 1). Its sequence similarity to OC116 of the Phasianidae family was rather low with only approximately 34 % identity, but the presence of phosphorylated amino acids (see below) supported its identification. Chicken OC116 was also detected in bone and cultured bone cells [86, 87] and is thus not truly eggshell-specific. In the chicken eggshell matrix OC116 was localized throughout the palisade layer and the mammillary layer [19] and reached its highest concentration in the growing eggshell during formation of the palisade layer [40]. In vitro crystallization experiments indicated a direct interaction of OC116 with calcium carbonate [17], but any molecular detail of its proposed regulatory function in eggshell mineralization is lacking at present.

Zebra finch ovocalyxin-36 (OCX36) and ovocalyxin-32 (OCX32) were also previously predicted correctly from published database sequences [8] and were among the major proteins of the zebra finch eggshell matrix. OCX36 was also a major component of other analyzed matrices, but OCX32 was not identified in turkey and quail. Its presence in the eggshell matrix of a member of a very different lineage indicates that failure to identify this protein previously in turkey and quail was due to the absence of its sequence in databases, but not necessarily its absence in eggshell matrix itself. The sequence identity of zebra finch ovocalyxins to chicken homologs was 58 % and 56 %, respectively. OCX36 occurs throughout all eggshell layers including the membranes [23] but seems to be enriched in eggshells of the terminal phase of shell mineralization (16 h; [40]) and also belongs to the most abundant proteins in eggshell cuticle [34, 88]. As a member of the LBP/BPI/PLUNC-like family [24] OCX36 was suggested to be part of the anti-microbial egg defense and indeed the isolated protein was shown to bind bacterial lipopolysaccharide (LPS) and to inhibit growth of Staphylococcus aureus [25]. It also modulates immune responses [26], but it is not known if, and how, this may be related to eggshell formation. OCX32 is most abundant in the matrix of shells when large calcite crystals form [40] and is present in the outer palisade layer, the vertical crystal layer and the cuticle [27]. In the latter, OCX32 was a major protein [34, 88]. OCX32 was linked to eggshell strength and mammillary layer thickness in several genetic studies [89–91]. However, its specific function in shell mineralization is unknown at present.

Two less well characterized ovocalyxins occasionally appearing in the literature are the predicted protease inhibitor ovocalyxin-25 (OCX25) and ovocalyxin-21 (OCX21) [39]. Both proteins were not identified in eggshell matrices other than that of chicken. While sequences of OCX25 homologs seem to be missing from zebra finch, turkey and quail databases, this is different for OCX21. This protein is identical to gastrokine-2 (GNK2; E1C2G7_CHICK) [34, 39], a protein of the gastric mucosal secretome, and was among the most abundant chicken eggshell proteins ([30], IPI00574331.1). Similar to turkey and quail the zebra finch database contains a protein fragment (H0Z0L8_TAEGU) with high similarity to other GNK2 proteins, and also contains the Brichos domain found in GNK2. In conclusion, the absence of this protein from eggshell matrices other than chicken suggests that its role in eggshell mineralization may not be generally essential.

### Major egg white proteins in the zebra finch eggshell matrix

Egg white proteins were among the first chicken major eggshell matrix proteins identified (see Background section) and were also identified in all other analyzed eggshell matrices (Table 1). Among the major proteins of zebra finch eggshell matrix with a percentage >1 % identified by LC-MS/MS were three proteins similar to ovalbumin (gi|224045100, gi|224045098, gi|224045096/H0YV21). Of these, ovalbumin-like protein gi|224045100 was the most similar to other ovalbumins and the most abundant component of the eggshell matrix (Additional file 3). Its identification as the ovalbumin homolog was confirmed by the identification of ovalbumin’s phosphorylation sites conserved across different species [92] (see below). The other entries most probably encode for ovalbumin-like proteins Y and X, respectively. The function of ovalbumin in eggshell mineralization, if any, is not clear at present. There are many reports of effects of this protein on in vitro crystallization of calcium carbonate [93–95], although these effects may be weak when compared to those of members of the so-called eggshell-specific group of proteins [17].

Next in abundance after ovalbumin-like proteins and OC116 followed the protein of accession H0Z7I9/gi|449486399 that was previously suggested to be the zebra finch homolog of protein Tenp [96]. Tenp (Transiently expressed in neural precursors) was first detected in the developing retina and brain of chicken embryos [97]. It was then shown to occur in chicken egg white in the first proteomic study of egg white [98] and more recently in emu egg white [99]. It was also identified in chicken egg yolk [100] and vitelline membrane [101]. The eggshell matrices of chicken [30] and turkey [52], but not that of quail [53], contained small amounts of Tenp. The presence of a bactericidal/permeability increasing domain (BPI) in the sequence of Tenp identifies it as a member of the BPI-like family of innate immune proteins and its antimicrobial activity was confirmed recently [99].

Other abundant egg white proteins in zebra finch eggshell matrix were proteins similar to ovomucoid, ovostatin, ovotransferrin, ovoinhibitor, avidin, cystatin, and riboflavin-binding protein. The abundance of these proteins in different matrices was variable (Table 1). All of these proteins show antimicrobial activity [102, 103] and may contribute to egg defense in the eggshell gland during matrix assembly and calcification. Lysozyme C, another major egg white protein with antibacterial properties, was highly abundant in the eggshell matrices of chicken, turkey and quail [53], but was identified in zebra finch eggshell matrix only as a minor component (Additional file 3). The possible biological significance of this discrepancy is unknown at present.

### Other major proteins

An important protein with respect to biomineralization is carbonic anhydrase [104]. This enzyme catalyzes the reversible hydration of CO_2_, thereby providing HCO_3_^−^ for CaCO_3_ precipitation. The zebra finch eggshell enzyme (H0ZCC0) was most similar to carbonic anhydrase IV (CA4), a membrane-associated extracellular α-carbonic anhydrase isozyme with a GPI (glycosylphosphatidylinositol) anchor. This was confirmed by prediction of a GPI attachment site at G_289_ of H0ZCC0 and a secretion signal sequence in aa1-18. A carbonic anhydrase IV isozyme was also identified at a similar abundance in turkey eggshell matrix and at a lower abundance in the matrices of quail and chicken eggshell (Table 1). A link to biomineralization is also known for calcitonin gene-related peptide 2 (CALCB; H0ZDU9), which was, however, difficult to differentiate from calcitonin itself (CALCA) by sequence comparisons because of its similarity to both. H0ZDU9 was a major protein in zebra finch eggshell matrix but no homolog was identified in turkey and chicken eggshells and only traces of a similar protein were found in quail eggshell matrix (Table 1). The identified peptides were distributed over the whole sequence of the protein with the exception of the secretion signal sequence. However, the most abundant peptides were from the N- and C-terminal propeptide regions, while evidence for the presence of the hormone itself (aa82-113 of the precursor) was scarce. Calcitonin and related peptides were shown to regulate bone formation in mouse knockout mutants [105]. However, there does not appear to be any evidence for the involvement of the propeptides in this effect. For the N-terminal propeptide contradicting evidence for a role as a bone cell mitogen have been published [106, 107]. Thus, a possible function of the propeptides in zebra finch eggshell matrix remains unknown.

### Missing proteins

Apart from the missing so-called eggshell-specific proteins OCX25 and OCX21 discussed above, several other major proteins previously suggested to have a function in eggshell matrix assembly or mineralization in the family Phasianidae were not identified. Most surprisingly, osteopontin, a member of the SIBLING family of mineralization-related secreted phosphoproteins [21, 22], was not identified. This widespread protein was identified as a major protein of the shell matrix of all three phasianid species [30, 31, 52, 53]. The involvement of this multifunctional protein in mineralization processes was reviewed previously [22, 108, 109]. Its activity is generally inhibitory and depends on phosphorylation. The localization of osteopontin in chicken eggshell predominantly at the surface of mammillary cones and eggshell pores and at the margins of calcite columns of the palisade layer supports an inhibitory function in eggshell mineralization by binding to selected crystal surfaces [110]. The zebra finch databases contain sequences similar to chicken osteopontin (H0YVH9, 67.8 % identity; gi|224049272, 54 % identity) If one of these is a homolog of chicken osteopontin, this protein may in fact be absent from the shell proteome of zebra finch. Other major proteins proposed previously to have a function in chicken, turkey and quail eggshell matrix because of their high abundance in all three, but apparently absent in zebra finch eggshell matrix, include EDIL3 (H0Z8Z8, 94.4 % identity to chicken protein), lactadherin/MFGE8 (H0ZD74, 88.7 % identity), and the extracellular chaperone clusterin (H0ZIU4, 28 % identity). However, the latter has a very low similarity to chicken clusterin and may not represent the zebra finch clusterin homolog of the chicken eggshell and egg white protein [111]. No zebra finch homolog was found in databases for the major chicken eggshell matrix protein EXFAB (extracellular fatty acid-binding protein) that is consequently also missing in the proteome.

### Zebra finch eggshell phosphoproteins

We showed previously that higher energy collisional dissociation (HCD) fragmentation, also used in the present report, is well suited to determine peptide phosphorylation sites [112]. Applied to low-complexity proteomes such as those of biomineral matrices, this fragmentation technique can yield useful information about major phosphoproteins and their phosphorylation sites without prior enrichment of modified peptides [53, 113]. Because phosphorylation was reported previously to potentially affect biomineralization processes [114–116]) Ser, Thr and Tyr phosphorylation was included among the variable modifications used for MaxQuant search of raw files.

Zebra finch eggshell matrix yielded fewer phosphoproteins and phosphosites than chicken [31] and quail [53] eggshell matrix. However, phosphopeptides were not enriched before analysis as in chicken, and the most important shell matrix phosphoprotein of chicken and quail matrix, osteopontin, was not identified in zebra finch eggshell (see above). Overall, 17 phosphosites in eight phosphoproteins (Table 2) were identified. Nine of the phosphosites agree with the predominant consensus motif for phosphorylation by the secreted kinase FAM20C (S/T-X-[D,E,pS], a kinase that appears to be identical to Golgi casein kinase and is known to modify many biomineralization-related extracellular proteins, such as the members of the SIBLING cluster [117, 118]. However, some other kinase target sequences not matching this motif are also recognized by FAM20C [119]. FAM20C was also identified as minor zebra finch eggshell matrix protein (Additional file 3). Other kinase motifs detected in phosphopeptides were those recognized by protein kinases PKG, PKC, or CK I.

**Table 2:** Phosphoproteins and phosphosites in zebra finch eggshell matrix

Accession (protein)	Peptide	Number of P-sites	Proba-bility ^1^	Mod/unmod
Gi|224045100 Ovalbumin-like	_57_AIHFDKIPGFGE**pS**VESQCGASVSIHNSLK_86_	1	1	221/5
	_63_IPGFGE**pS**VESQCGASVSIHNSLK_86_	1	1	748/5
	_342_VAG**p[** **S** **S]**GAGVDDTSVSEEIR_360_	1	[1]	37/1333
Gi|224049274/H0YVI0 Ovocleidin-116	_94_REPPAGSAGTAPEHSDN**p[** **S** **ST]**EVVEYGIVFKPK_125_	1	[1]	123/124
	_228_GAGDEG**pS**GEATVSGQGQEGVK_248_	1	1	445/169
	_249_QGTGTGGVAL**p[S** **S** **]**VTEK_265_	1	[>0.9]	51/681
	_355_RLDVTAAP**pS**GEDDSIPTHR_373_	1	0.99	3/40
	_387_GDSVA**pS** SLR_395_	1	0.85	1/59
	_396_DGHL**pT**GEDEEGATTIGVDR_414_	1	1	7/543
	_508_GG**pS**GEVGTTTSRPSR_522_	1	1	9/3
	_546_VDTAPSPSGKPSGWASSGAQTSAGGHGDAGR**p**[**S**HA**S** ]K_582_	1	[1]	21/0
	_556_PSGWASSGAQTSAGGHGDAGR**p**[**S**HA**S**]K_582_	1	[1]	15/15
Gi|224057610/H0Z847 Vitellogenin-2	_1056_IINEVNAE**pS**EEEGESSLYGDMK_1077_	1	1	3/0
	_1550_MPA**pS**ELQPPIWNVFAAPSAVLENLK_1575_	1	1	34/1
Gi|224057612 Vitellogenin-2-like	_1218_AM**pS**QPEFLGDSKPPILAAVLR_1238_	1	1	1/21
H0Z8U7/gi|224057648 Similar to vitellogenin-1	_1252_ TA**pS**FPLASAAEGER_1265_	1	1	35/17
Gi|449474881 Similar to ovoinhibitor	_**602**_ **pS**GEAIAACPYILR_614_	1	1	35/10
H0Z5S2/gi|449479753 Similar to PEDF	_291_E**pT**RLQSLFTSPDFSK_305_	1	1	1/0
Gi|449480130/H0Z5Q3 Similar to alpha-2-antiplasmin	_2374_EAQD**pS**REATDANEYRVPK_2391_	1	1	1/0

Phosphorylation sites are presented in bold print. In some cases it was not possible to unequivocally identify the exact phosphorylation site. One cause may be partial phosphorylation of neighboring target residues as shown in square brackets. Amino acid residues with the higher prediction score [74] are underlined in such instances. Underlined amino acids outside square brackets indicate alternative minor phosphorylation sites with a probability lower than 0.75. 1, only the highest location probability (MaxQuant) is indicated

**Fig. 3:**
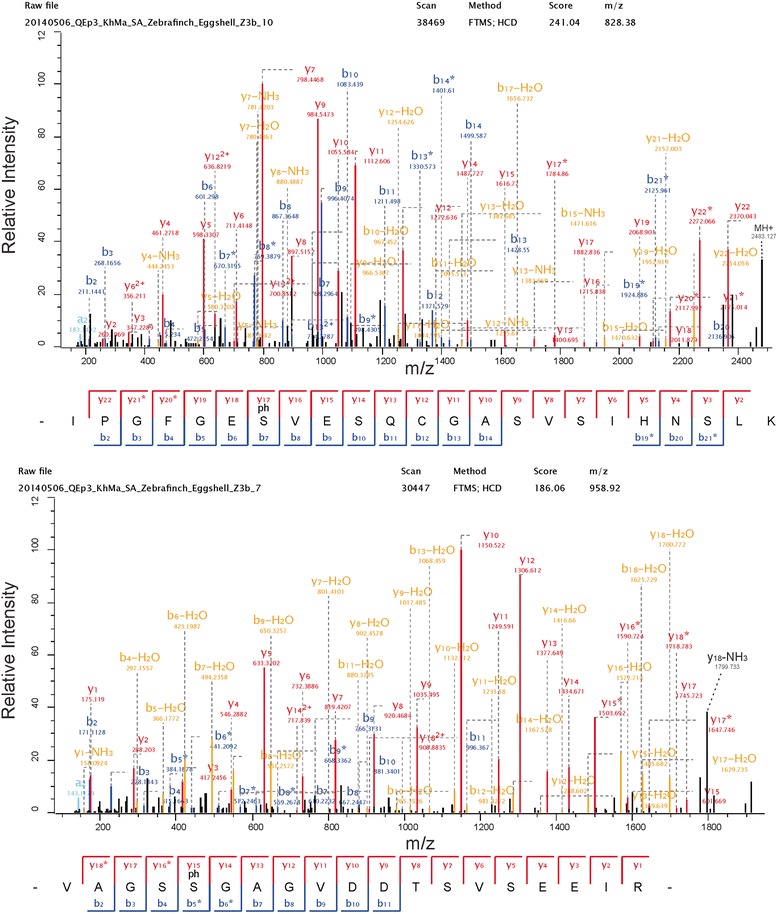
Ovalbumin phosphopeptide spectra. The spectrum on top is from a triply charged peptide with a mass error of 0.19 ppm and a PEP of 1.2e-178. The spectrum below is shows the fragments of a doubly charged peptide with a mass error of 0.40 ppm and a PEP of 8.9e-62. Y-ions are shown in red, b-ions in blue, and fragments with neutral loss of ammonia or water are in orange. A few additional annotations of major fragments not annotated automatically are in black. Asterisks indicate loss of the phospho group. These fragments are important for site location

**Fig. 4:**
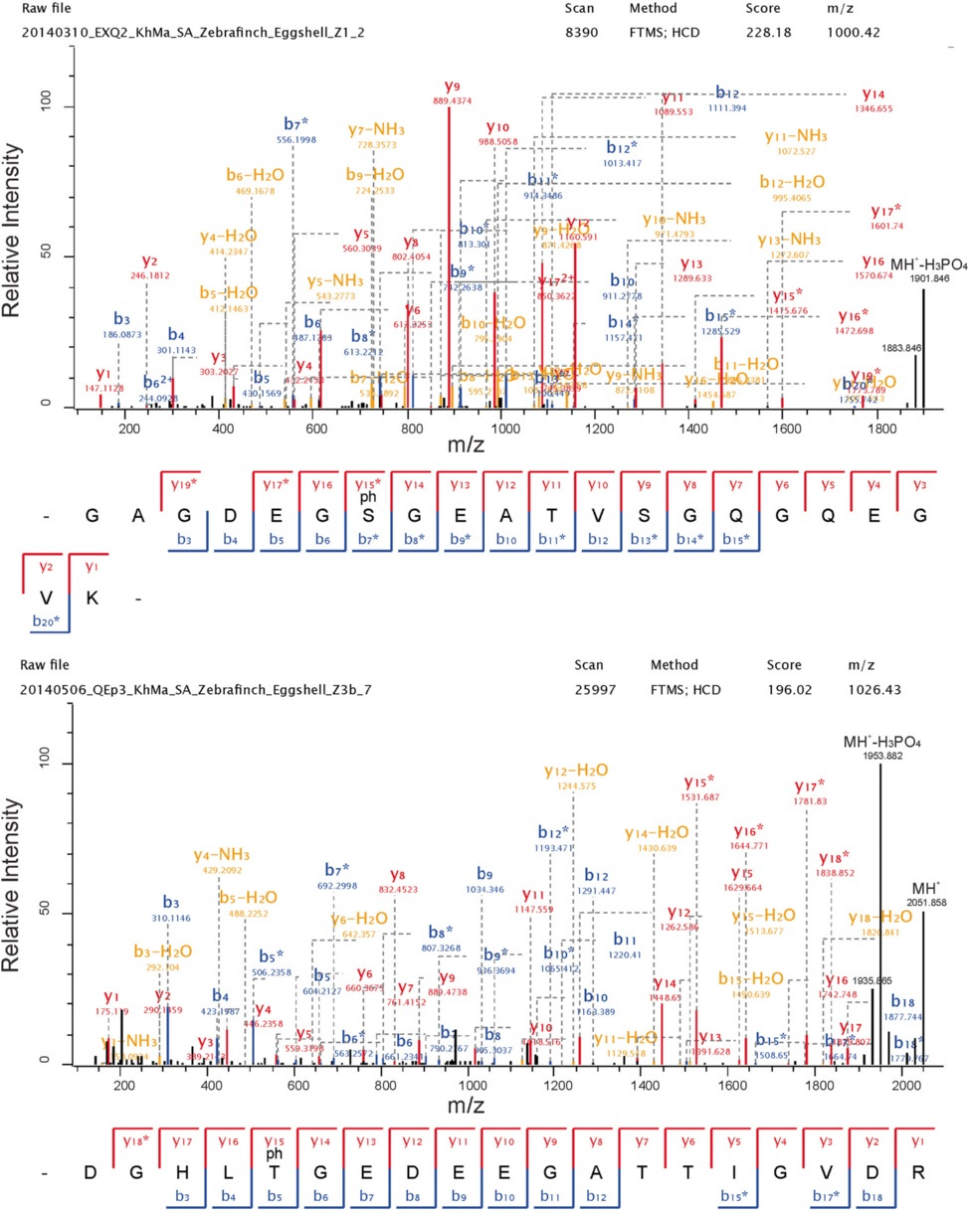
Some selected ovocleidin-116 phosphopeptide spectra. Spectra of to two doubly charged OC116 phosphopeptides. The peptide on top has a mass error of 0.24 ppm and a PEP of 3.6e-144. The peptide below was measured with a mass error of >0.02 and has a PEP of 8.4e-145. Y-ions are shown in red, b-ions in blue, and fragments with neutral loss of ammonia or water are in orange. A few additional annotations of major fragments not annotated automatically are in black. Asterisks indicate loss of the phospho group

**Fig. 5:**
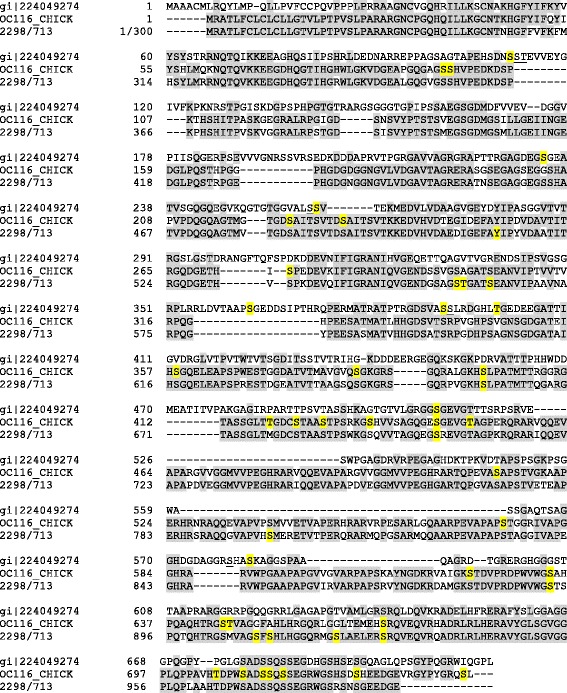
Alignment of OC116 sequences and phosphorylation sites. Comparison of sequences and identified phosphorylation sites of zebra finch OC116 (gi|224049274), chicken OC116 (OC116_CHICK); [31], and quail OC116 (entries 2298/713; [53]). Identical amino acids are shown on grey background and phosphorylated amino acids are shown on yellow background

Two phosphorylation sites detected in ovalbumin-like protein gi|224045100 correspond to previously identified ovalbumin phosphorylation sites [92]. This can be used as a diagnostic feature to identify the ovalbumin homolog among three highly abundant ovalbumin-like proteins in zebra finch eggshell matrix (Table 1). Selected phosphopeptide spectra for both sites are shown in Fig. 3. The major phosphoprotein of the zebra finch eggshell was ovocleidin-116 with at least eight phosphorylation sites (Table 2). Typical spectra for two of them are shown in Fig. 4. Conservation of sites among the species is low. Only a single Ser was phosphorylated in all three ovocleidins (zebra finch, chicken and quail; Fig. 5), indicating that overall phosphorylation may be more important than site conservation.

**Fig. 6:**
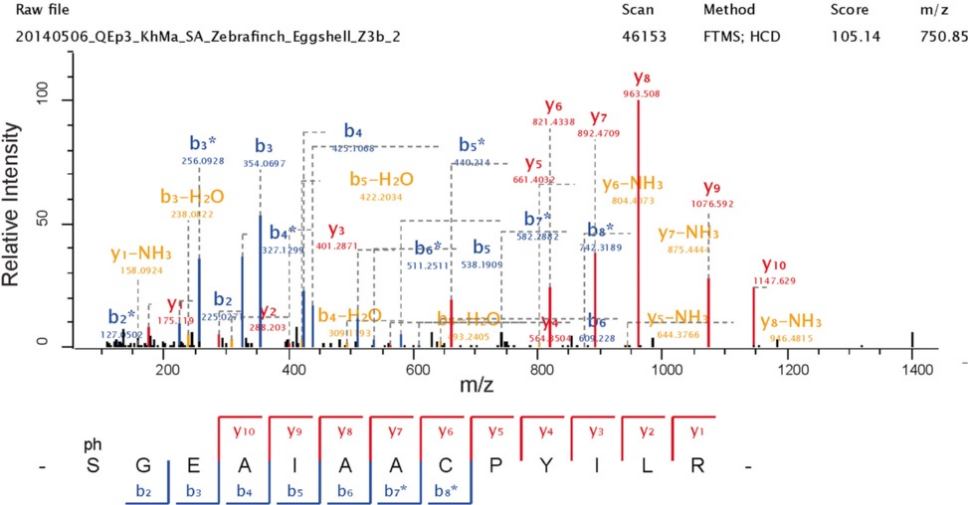
Ovoinhibitor phosphopeptide spectrum. This doubly charged peptide was measured with a mass error of 3.23 ppm and a PEP of 0.002. Y-ions are shown in red, b-ions in blue, and fragments with neutral loss of ammonia or water are in orange. Asterisks indicate loss of the phospho group

**Fig. 7:**
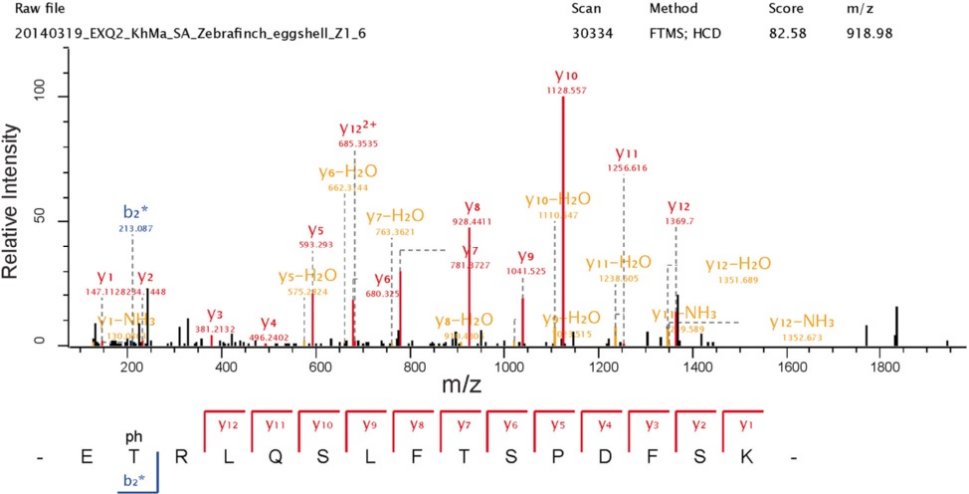
Phosphopeptide spectrum of a protein similar to PEDF. The doubly charged peptide has a mass error of 4.35 ppm and a PEP of 0.0003. Y-ions are shown in red, b-ions in blue, and fragments with neutral loss of ammonia or water are in orange. Asterisks indicate loss of the phospho group

**Fig. 8:**
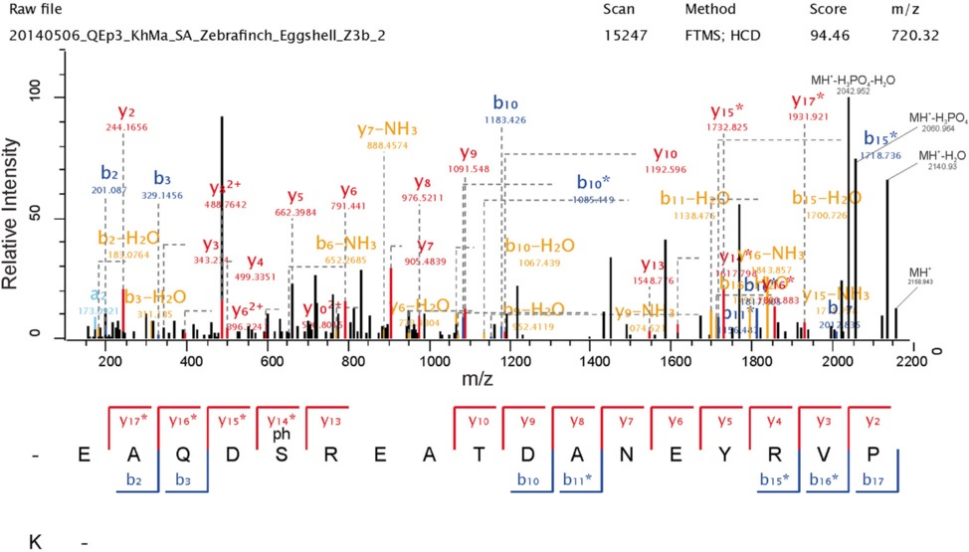
Phosphopeptide spectrum of similar to alpha-2-antiplasmin. This figure shows the triply charged single alpha-2-antiplasmin phosphopeptide with a mass error of 0.83 ppm and a PEP 0f 3.76e-7. Y-ions are shown in red, b-ions in blue, and fragments with neutral loss of ammonia or water are in orange. A few additional annotations of major fragments not annotated automatically were added in black. Asterisks indicate loss of the phospho group

Four phosphorylation sites were identified in vitellogenins (Table 2), all from outside the heavily phosphorylated phosvitin part of the precursor. One of the two vitellogenin-2 phosphorylation sites, S_1064_, corresponds to VIT2_CHICK S_1064_ that was reported to be phosphorylated previously [31]. The other three sites were not reported previously although a phosphorylation site was also identified in quail vitellogenin-1 in approximately the same region [53]. A novel phosphorylation site was identified in a protein similar to ovoinhibitor (Table 2; Fig. 6), a protein that was not reported to be phosphorylated previously. A single phosphorylation site identified in a protein similar to PEDF (Table 2; Fig. 7) was not the same as phosphorylation sites previously reported for this protein in chicken [31]. This peptide occurred only once and contained one missed cleavage. While the non-phosphorylated C-terminal product of complete cleavage, LQSLFTSPDFSK, was identified 68 times, the N-terminal phosphorylated sequence alone, EpTR, was too short to yield an identifiable peptide. Thus, identification of this phosphorylation site depended on miss-cleavage by trypsin, which may explain its low frequency. Similarly, the phosphopeptide of gi/449480130/H0Z5Q3 (similar to α-2-antiplasmin) occurred only once and was the result of two missed cleavages (Table 2; Fig. 8). The un-phosphorylated cleavage products EATDANEYR and EATDANEYRVPK were identified 27 and 11 times, respectively. MaxQuant phosphopeptide data are shown in Additional file 4: Phospho(STY)Sites, and include data of identifications not accepted after manual validation or failure to agree with the thresholds defined in Materials and Methods.

## Conclusions

The major purpose of this study was to analyze the zebra finch eggshell matrix to determine the conservation of important proteins, such as the so-called eggshell-specific proteins, among avian species other than Phasianidae. The conservation of chicken OCX32, OCX36, OC116 and, possibly, OC17 homologs in zebra finch eggshell points to the importance of these proteins for eggshell mineralization and indicates that previous failure to identify some the proteins in turkey or quail was due to the absence of the sequences in the respective databases, but not the eggshell matrix itself. The same may be true for other proteins thought to be important for eggshell mineralization, such as osteopontin. These results underline the need for more comprehensive sequence databases for future research, enabling analysis of the proteomic inventory of eggshell matrices from other species to possibly define a common eggshell mineralization toolkit.

The exact role in mineralization played by the identified proteins is not clear at present and cannot be elucidated by proteomic or transcriptomic studies, although the abundance of particular proteins in different shell compartments [34, 35], in uterus fluid [39] and eggshell matrix [40] at different stages of mineralization, or the transcriptomic and proteomic exploration of eggshell protein expression differences linked to particular traits, such as eggshell strength [40, 89, 91], may yield initial clues. The same applies to the popular in vitro calcium carbonate precipitation and crystallization assays that have also been applied to isolated eggshell proteins [12, 17, 27, 78] but seem to be rather tentative and vague and may therefore be complemented by modern electron microscopic techniques, such as atomic force microscopy, to study at higher resolution the assembly of matrix on substrates or mineral nucleation on organic matrices [120]. Another approach to gene and protein function is gene knockouts that have been used extensively in laboratory animals like mouse, but are also applicable to chicken [121, 122]. The latter study is particularly interesting because genetic engineering was applied to the ovalbumin gene. Ovalbumin is a major protein not only of egg white, but also of avian eggshell matrices. Finally, there are examples from other biominerals than eggshell showing that matrix proteins form functional complexes [123]. Thus it may be promising to study protein-protein interaction in the eggshell matrix by such techniques as immunoprecipitation and affinity purification combined with mass spectrometric analysis of binding partners [124, 125] or mass spectrometric analysis of native complexes with and without cross-linking of interacting proteins [126–128].

## Additional files

Additional file 1:
**ProteinGroups.** This table contains MaxQuant ProteinGroup output and contains the complete list of identifications, including those not accepted after manual validation or because they did not match the thresholds defined in Materials and Methods. The table also includes relevant additional data such as the complete set of accession numbers forming one group, the calculated molecular weight of each accession, iBAQ intensities and scores. (XLS 687 kb) Additional file 2:
**Peptides.** This table contains all peptides of identifications in Additional file 1 in alphabetic order starting with the first amino acid. It also contains additional data such as scores, charge state, and mass accuracy. (XLS 2094 kb) Additional file 3:
**Accepted identifications.** This table shows all accepted identifications with annotation derived from FASTA searches, iBAQ-derived abundances, number of unique and total peptides, and presence of the protein in eggshell matrices of other species. (DOCX 231 kb) Additional file 4:
**Phospho(STY)sites.** This file contains the MaxQuant output for phosphopeptides and also includes peptides not accepted after manual validation. The data include phosphopeptide sequences, localization probabilities and scores. (XLS 95 kb)

## Abbreviations

Aa: Amino acid
FDR: False discovery rate
HCD: Higher-energy collision-induced dissociation
iBAQ: intensity-based absolute quantification
MS/MS: tandem mass spectrometry
OC: Ovocleidin
OCX: Ovocalyxin
PAGE: Polyacrylamide gel electrophoresis
SIBLING: Small integrin-binding ligand N-linked glycoproteins
TOF: Time-of-flight
