# Supplementary material for: The calcified eggshell matrix proteome of a songbird, the zebra finch (Taeniopygia guttata)
Source: Proteome Sci. 2015 Dec 1;13:29. doi: 10.1186/s12953-015-0086-1 (PMC4666066; doi:10.1186/s12953-015-0086-1)
Supplement: Additional file 3: — Accepted identifications. This table shows all accepted identifications with annotation derived from FASTA searches, iBAQ-derived abundances, number of unique and total peptides, and presence of the protein in eggshell matrices of other species. (DOCX 231 kb) [file 12953_2015_86_MOESM3_ESM.docx]

**Table S1**

**Zebra finch eggshell shell matrix proteins**

| **Accession** | **Protein** | **Most similar chicken^1^, turkey^1^**  **and quail^2^ proteins** | **% identity** | **e-value** | **Unique and razor peptides** | **Total peptides** | **% of total**  **(iBAQ)** | **Presence in other avian shell proteome** |
| --- | --- | --- | --- | --- | --- | --- | --- | --- |
|  |  |  |  |  |  |  |  |  |
| **B5G4G7**  **gi\|197129680** | Putative tubulin β-2/3 (TUBB3); shares 10 peptides with H0YVD4 (TUBB2A-1) | F1NYB1_CHICK  G1MYE7_MELGA  (5568) | 100.0  97.3  91.0 | 5.0e-195  7.9e-190  0e0 | 15 | 224  (Z1,2,3, 3b) | 0.07 | C,T |
| **H0Z8N9**  **gi\|224094009** | Ras-related protein Rap-1b (RAP1B) | RAP1B_CHICK  G1NDE6_MELGA  (23291) | 100.0  100.0  94 | 4.9e-63  4.9e-63  1e-56 | 2 | 11  (Z1,3,3b) | 0.02 | C,T |
| **B5FX76**  **gi\|350537703** | Putative retinoic acid receptor responder (tazarotene induced) 2 (RARRES2) | F1NIP4_CHICK | 72.5 | 2.4e-51 | 3 | 39  (all) | 0.02 | C |
| **B5FXC8**  **gi\|350536313** | Histone H4 | H4_CHICK  G1NRJ0_MELGA  25266 | 100.0  100.0  100 | 1.2e-38  1.2e-38  2e-54 | 6 | 60  (all) | 0.14 | C,T,Q |
| **B5FXD7**  **gi\|350535761** | Ras-related protein ral-A (RALA) | G1NI18_MELGA  E1C524_CHICK  9087 | 99.5  99.5  84 | 1.4e-68  1.4e-68  5e-78 | 2 | 5  (Z1,3b) | <0.01 | T,Q |
| **B5FXE8**  **gi\|350536229** | Annexin (A4 variant 2) ; shares 1 peptide with H0Z7J3 | H9KZI4_CHICK  H9H0B3_MELGA  23558 | 88.4  92.6  88 | 2.0e-116  8.3e-101  8-46 | 4 | 12  (Z1,3b) | <0.01 | C,T,Q |
| **B5FXS4**  **gi\|350537817** | Putative corticotropin releasing hormone (CHR) | Q703P0_CHICK  G3US39_MELGA | 75.4  73.3 | 9.5e-26  3-0e-6 | 3 | 19  (Z1,2,3b) | 0.01 |  |
| **B5FXT6**  **gi\|350539463** | Ribosomal protein SA (RPSA)/37kDa laminin receptor | G1NGU2_MELGA  RSSA_CHICK  6069 | 99.7  99.7  100 | 4.3e-115  4.3e-115  3e-93 | 3 | 10  (Z2,3,3b) | <0.01 | T,Q |
| **B5FY92**  **gi\|350538677** | Putative myosin regulatory light chain isoform L20-B variant 1 or 2/MYL9/MRCL3 | E1C6R9_CHICK  G1NB45_MELGA  13689 | 100.0  99.4  93 | 1.1e-66  1.8e-66  2e-93 | 3 | 3  (Z1,3b) | <0.01 | T |
| **gi\|224165426**  **B5FYB6** | Putative stem cell antigen 2 variant 2 /LY6E-2 | G1NJZ3_MELGA  E1BWP5_CHICK | 78.6  75.4 | 8.0e-42  5.7e-40 | 2 | 30  (all) | 0.04 |  |
| **B5FYJ8**  **gi\|350535014** | Putative myelin protein zero-like 1 variant 1 (MPZL1) | G1NNN7_MELGA  E1C603_CHICK  22094 | 86.7  86.7  86 | 2.0e-64  8.9e-64  3e-60 | 5 | 84  (all) | 0.10 | C,Q |
| **H0ZBT5**  **gi\|350539159** | Lactoylglutathione lyase/Glyoxylase 1 (GLO1) | F1N9Q2_CHICK  G1NDZ9_MELGA  22249 | 95.5  84.4  96 | 1.8e-68  8.4e-58  3e-73 | 5 | 46  (Z1,3,3b) | 0.02 | T |
| **gi\|197127712**  **H0ZF58** | Similar to Ig lambda light chain | G1N5T4_MELGA  R9PXM5_CHICK  25336 | 65.3  64.9  50 | 8.9e-60  1.9e-57  6e-24 | 4 | 35  (Z1,2,3b) | 0.01 | C,T,Q |
| **H0ZH50**  **gi\|350534704** | Putative N-acetylglucosamine-1-phosphotransferase γ-subunit variant 2 or 3 (GNPTG) | G1N506_MELGA  E1BS68_CHICK  13255 | 95.1  89.0  63 | 4.3e-119  1.3e-101  5e-34 | 5 | 60  (all) | 0.02 | C,T |
| **H0Z5L8**  **gi\|350539461** | Actin-related protein 2/3 complex subunit 3 (ARPC3) | E1C8Y3_CHICK  G1MXF3_MELGA  23402 | 97.7  97.7  82 | 1.3e-80  1.5e-80  2e-63 | 2 | 14  (Z2,3b) | 0.01 | C |
| **B5G027**  **gi\|197128146** | Putative tyrosine 3-monooxgenase/ tryptophan 5-monooxgenase activation protein γ polypeptide variant 1/14-3-3 gamma (YWHAG); shares 5 peptides with other 14-3-3 proteins | 1433G_CHICK  G1MX52_MELGA  13883 | 99.6  100.0  100 | 2.9e-98  1.7e-86  1e-123 | 2 | 7  (Z1,3b) | 0.01 | Q |
| **B5G0H9**  **gi\|325652120** | Nucleoside diphosphate kinase | NDK_CHICK  G1N8T1_MELGA  20396 | 93.5  91.5  94 | 6.5e-63  1.6e-61  3e-59 | 6 | 60  (Z1,3,3b) | 0.05 | C,T,Q |
| **B5G0M2**  **gi\|197128341** | Putative peroxiredoxin 1, variant 2; shares 1 peptide with H0ZAF6 | PRDX1_CHICK  G1NDB7_MELGA  19644 | 97.5  96.5  95 | 1.6e-88  3.7e-88  4e-83 | 11 | 256  (all) | 0.21 | C,T,Q |
| **B5G0S3**  **gi\|350537761** | Putative peroxiredoxin 6 | F1NBV0_CHICK  G1MYX6_MELGA  18867 | 93.8  93.8  93 | 1.9e-92  5.9e-78  2e-74 | 10 | 66  (all) | 0.02 | C,T,Q |
| **B5G106**  **gi\|350534770** | Putative β-globin; shares 3 peptides with CON | HBB_CHICK  P84479_MELGA  20126 | 92.5  91.1  97 | 2.1e-60  2.3e-59  5e-81 | 6 | 24  (Z2,3b) | 0.02 | C,T,Q |
| **B5G117**  **gi\|397529593** | Similar to thioredoxin | THIO_CHICK  G1N8X0_MELGA  28900 | 87.6  90.6  70 | 2.5e-40  6.6e-38  1e-31 | 2 | 10  (Z1,3b) | 0.02 | C |
| **H0YVJ6**  **gi\|197128543** | Malate dehydrogenase | G1N071_MELGA  MDHC_CHICK  18583 | 98.8  98.8  98 | 1.2e-139  1.2e-139  6e-86 | 7 | 65  (all) | 0.03 | C,T,Q |
| **B5G1C3**  **gi\|197128586** | Putative myosin alkali light chain 6 smooth muscle form variant 1 | Q6W5H0_MELGA  MYL6_CHICK  15120 | 98.7  98.7  75 | 1.0e-59  1.2e-59  2e-57 | 3 | 11  (Z1,3b) | 0.01 | C |
| **B5G1H7**  **gi\|350537173** | Putative RAB5C member RAS oncogene family variant 3 | G1MWE3_MELGA  RAB5C_CHICK  14057 | 99.5  99.5  99 | 1.8e-84  1.8e-84  1e-121 | 3 | 13  (Z3,3b) | 0.01 | C,T,Q |
| **B5G4N1**  **gi\|197129744** | Putative calmodulin variant 1 | CALM_CHICK  G1NK53_MELGA  17243 | 94.6  94.6  94 | 1.0e-44  2.8e-44  2e-68 | 6 | 131  (all) | 0.09 | C,T |
| **B5G1N3**  **gi\|350539033** | Similar to β-2-microglobulin | Q6L754_CHICK  B2MG_MELGA | 50.9  50.0 | 9.3e-25  1.3e-20 | 3 | 52  (Z1,2,3, 3b) | 0.08 | C |
| **H0Z6G7**  **gi\|350524686** | Destrin | G1N515_MELGA  DEST_CHICK  17268 | 99.4  98.8  99 | 1.2e-73  2.7e-73  1e-92 | 4 | 17  (Z1,2,3, 3b) | 0.01 | C,T,Q |
| **H0Z8F6**  **gi\|350534852** | Ras-related protein rab-14 | G1N6Z1_MELGA  RAB14_CHICK  16957 | 100.0  100.0  59 | 2.2e-80  2.2e-80  8e-66 | 2 | 3  (Z1,3b) | <0.01 |  |
| **B5G276**  **gi\|350537141** | Transthyretin | TTHY_CHICK  G1NDK1_MELGA | 86.7  72.7 | 3.4e-56  1.9e-46 | 2 | 18  (Z2,3,3b) | 0.02 | C |
| **B5KFL8**  **gi\|350534650** | Triosephosphate isomerase (TPI) | TPIS_CHICK  Q70I42_MELGA  14790 | 98.1  97.5  78 | 4.4e-89  2.1e-80  9e-89 | 4 | 7  (Z2,3,3b) | <0.01 | C,T,Q |
| **B5G2G3**  **gi\|310703675** | L-lactate dehydrogenase | LDHB_CHICK  G1NLS0_MELGA  22431 | 88.3  82.9  84 | 1.6e-123  8.7e-115  6e-57 | 11 | 150  (all) | 0.11 | C,T,Q |
| **B5G2Q3**  **gi\|5822013** | Ubiquitin (C) | G1NR52_MELGA  RS27A_CHICK  11531 | 100.0  100.0  100 | 8.3e-28  8.4e-28  6e-38 | 5 | 204  (all) | 1.06 | C,T,Q |
| **B5G2T1**  **gi\|350537671** | Putative creatine kinase B variant 1 | KCRB_CHICK  G1NL16_MELGA | 95.3  95.0  81 | 1.5e-165  2.5e-165  0e0 | 8 | 42  (all) | <0.01 | C,T |
| **B5G356**  **gi\|350538495** | Putative apolipoprotein A-I | G1MVX1_MELGA  APOA1_CHICK  12041 | 83.3  82.2  81 | 2.3e-63  2.2e-62  1e-117 | 28 | 710  (all) | 0.98 | C,T,Q |
| **B5G368**  **gi\|197129295** | Putative cystatin variant 1 or 2 | CYT_CHICK  G1N522_MELGA  14227 | 67.9  65.2  66 | 5.4e-35  4.1e-33  1e-41 | 4 | 133  (all) | 0.22 | C,T,Q |
| **B5G3G3**  **gi\|350534666** | Peptidyl-prolyl cis-trans isomerase | R4GHX2_CHICK  G1N1Y0_MELGA  21947 | 95.2  83.9  96 | 2.4e-63  1.0e-54  4e-74 | 2 | 9  (Z1,3b) | <0.01 | C,T,Q |
| **B5G3N2**  **gi\|350538681** | Putative tubulin α-2 chain variant 2 (TUBA1C) | G1NFQ5_MELGA  TBA1_CHICK | 99.5  98.3  98 | 8.8e-184  2.4e-179  0e0 | 12 | 166  (all) | 0.07 | C |
| **B5G3P8**  **gi\|323668297** | Putative hemoglobin α-A | HBA_CHICK  HBA_MELGA  14194 | 80.3  80.3  64 | 3.3e-46  3.9e-46  3e-57 | 7 | 39  (Z1,3b) | 0.04 | C,T,Q |
| **B5G3Z6**  **gi\|228480202** | Putative Parkinson disease autosomal recessive early onset 7 variant 1 (PARK7/DJ-1) | G1MQE7_MELGA  D5M8S2_CHICK  11279 | 96.8  96.8  98 | 4.0e-73  4.0e-73  3e-97 | 2 | 4  (Z1,3b) | <0.01 | Q |
| **B5G418**  **gi\|350539567** | Putative actin-related protein 2/3 complex subunit 2 (ARPC2) | F1P1K3_CHICK  G1NGB7_MELGA  8830 | 99.3  91.1  99 | 6.7e-130  1.1e-116  1e-129 | 2 | 10  (Z1,3b) | <0.01 |  |
| **B5G464**  **gi\|350539029** | Putative neuroserpin variant 4 (SERPINI1) | NEUS_CHICK  G1ND93_MELGA  9774 | 93.4  92.7  92 | 2.8e-162  3.4e-162  1e-157 | 13 | 171  (all) | 0.08 | C,T,Q |
| **B5G472**  **gi\|350535745** | Ferritin (FTH1) | FRIH_CHICK  G1NB58_MELGA  17575 | 96.1  97.9  97 | 1.1e-74  1.4e-57  4e-93 | 3 | 23  (Z1,3b) | 0.01 | Q |
| **B5G4N8**  **gi\|350536587** | Peptidyl-prolyl cis-trans isomerase | PPIB_CHICK  18232 | 95.8  96 | 1.1e-76  1e-82 | 10 | 162  (all) | 0.19 | C,Q |
| **H0ZHR2**  **gi\|350539311** | Proteasome subunit β type (PSMB1) | G1NHW9_CHICK  Q6JLB2_MELGA | 96.6  96.1 | 9.8e-84  1.3e-83 | 3 | 11  (Z1,3b) | <0.01 | T |
| **B5KFQ2**  **gi\|197129902** | Putative CD9 antigen variant 2/tetraspanin | Q9IBC9_CHICK  G1NMA0_MELGA  A5HU02_COTCO | 78.6  78.6  76.4 | 1.1e-78  3.8e-77  4.6e-74 | 4 | 31  (Z1,3b) | 0.05 | C,T,Q |
| **B5KFT9**  **gi\|350535583** | Putative lysozyme C | LYSC_CHICK  LYSC_MELGA  LYSC_COTJA | 42.6  43.9  43.2 | 4.3e-28  1.2e-27  5.8e-29 | 5 | 57  (all) | 0.05 | C,T,Q |
| **gi\|224042875**  **H0ZGC4** | Similar to growth arrest-specific protein 6 (GAS6) | F1P3F0_CHICK  G1NPQ8_MELGA  3409 | 86.2  82.4  87 | 0e0  9.2e-137  0e0 | 12 | 220  (all) | 0.06 | C,T,Q |
| **H0ZHG1**  **gi\|224042906** | UDP-glucuronic acid decarboxylase 1 (UXS1) | E1BV28_CHICK  G1NPP3_MELGA  11592 | 99.0  97.8  100 | 4.5e-170  4.0e-142  1e-135 | 13 | 157  (all) | 0.07 | C,T,Q |
| **H0ZIS5**  **gi\|224042983** | α-1,3-mannosyl-glycoprotein 4-beta-N-acetylglucosaminyltransferase A (MGAT4A) | MGT4A_CHICK  G1NPK1_MELGA  7592 | 98.9  98.3  89 | 0e0  0e0  0e0 | 2 | 5  (Z1,3b) | <0.01 | T |
| **H0ZP19**  **gi\|224043319** | Similar to NHL repeat-containing protein 3 (NHLRC3) | F1P590_CHICK  G1NQ87_MELGA  20532/27081 | 83.6  84.5  82 | 1.4e-115  8.2e-88  6e-69 | 2 | 70  (all) | 0.07 | C,T,Q |
| **H0ZQM7**  **gi\|224043499** | Similar to ceroid-lipofuscinosis neuronal protein 5 (CLN5) | F1NZF1_CHICK  G1NPZ1_MELGA  17881/17749 | 89.7  91.7  91 | 1.7e-147  3.0e-138  6e-89 | 12 | 165  (all) | 0.11 | C,T,Q |
| **H0ZQZ6**  **gi\|224043550** | Similar to vitelline membrane outer layer protein 1 (VMO1) | VMO1_CHICK  G1NQI2_MELGA | 76.1  73.2 | 6.4e-68  9.4e-64 | 3 | 23  (Z1,3b) | 0.01 | C |
| **gi\|224043680**  **H0ZRS7** | Similar to lysosomal Pro-X carboxypeptidase (PRCP) | G1NQQ2_MELGA  F1NWF2_CHICK  6656 | 88.6  86.3  83 | 7.5e-188  1.7e-182  1e-119 | 10 | 193  (all) | 0.19 | Q |
| **gi\|224043738**  **H0ZS50** | Calpain-5 (CAPN5) | G1NQT4_MELGA  E1C292_CHICK  15925 | 95.9  95.4  98 | 0e0  0e0  1e-112 | 7 | 16  (Z2,3b) | <0.01 | C,T,Q |
| **H0ZSU0**  **gi\|224043866** | γ-enolase (ENO2); shares 3 peptides with H0Z0D8 (ENO1) | F1NG74_CHICK  G1NMW0_MELGA  6560 | 99.5  99.5  99 | 2.0e-167  2.0e-167  0e0 | 2 | 14  (all) | 0.01 |  |
| **H0ZT07**  **gi\|224043948** | V-type proton ATPase catalytic subunit (ATP6V1A) | F1NBW2_CHICK  G1NN61_MELGA  3432 | 99.8  99.7  94 | 0e0  0e0  0e0 | 5 | 20  (Z1,3b) | <0.01 |  |
| **H0ZT46**  **gi\|224044019** | β-1,4-galactosyltransferase 4 (B4GALT4) | E1C9B0_CHICK  G1NNC1_MELGA  28248 | 81.2  81.7  90 | 6.7e-130  7.8e-130  7e-35 | 4 | 18  (all) | <0.01 | T,Q |
| **gi\|224044051H0ZT64** | Similar to apovitellenin-1 (APOVLDLII) | G1NND5_MELGA  APOV1_CHICK  APOV1_COTJA | 58.5  57.5  54.7 | 1.9e-23  6.5e-21  1.0e-20 | 3 | 88  (all) | 0.16 | C,T,Q |
| **gi\|224044183**  **H0ZTD7** | A disintegrin and metalloproteinase with thrombospondin motifs 1 (ADAMTS1) | F1P3T6_CHICK  G1NNU1_MELGA  2489 | 86.9  95.4  96 | 0e0  0e0  0e0 | 7 | 39  (Z1,3,3b) | 0.01 | T,Q |
| **H0ZTJ7**  **gi\|224044274** | Similar to heat shock 70 kDa protein 13 (HSPA13) | G1NNS3_MELGA  F1ND59_CHICK  13580 | 82.6  82.0  79 | 4.6e-152  3.3e-150  3e-96 | 13 | 194  (all) | 0.08 | C,T,Q |
| **gi\|224044358**  **H0ZTP3** | T-complex protein 1 subunit theta (CCT8) | TCPQ_CHICK  G1NNU8_MELGA  7146 | 96.5  94.9  84 | 1.1e-210  1.2e-205  0e0 | 6 | 35  (Z1,3,3b) | <0.01 | T,Q |
| **gi\|224044540**  **H0YPN5** | Similar to ADP-ribosylation factor 1 (Fragment; ARF1) | G1MUL7_MELGA  F1NN08_CHICK  18374 | 100.0  100.0  100 | 4.4e-68  4.4e-68  2e-92 | 6 | 27  (all) | 0.01 | C,T,Q |
| **H0YTI8**  **gi\|224044919** | Similar to collagen α-2(I) chain (COL1A2) | F1P0H9_CHICK  G1NB83_MELGA  473 | 96.0  89.3  95 | 0e0  2.8e-185  0e0 | 3 | 12  (Z1,3b) | <0.01 | C |
| **gi\|224044975**  **H0YU13** | Similar to programmed cell death 6-interacting protein (PDCD6IP) | Q5ZJ70_CHICK  G1NH54_MELGA  8791 | 92.2  86.7  97 | 2.4e-148  9.7e-137  1e-188 | 25 | 190  (all) | 0.02 | C,T,Q |
| **gi\|224044985**  **H0YU54** | Similar to protein disulfide-isomerase A4 (PDIA4) | G1MRI5_MELGA  F1NDY9_CHICK  3258 | 94.1  94.7  93 | 5.9e-73  7.1e-73  0e0 | 11 | 49  (Z1,3,3b) | 0.01 | C,T,Q |
| **gi\|224045096**  **H0YV21** | Ovalbumin-like (OVALX?); shares 4 peptides with gi\|224045098 | R9TNA6_CHICK  G1MYK6_MELGA  9384 | 70.7  61.1  71 | 3.9e-120  1.1e-103  1e-127 | 10 | 1237  (all) | 4.08 | C,T,Q |
| **gi\|224045098** | Ovalbumin-related protein Y-like; shares 4 peptides with gi\|224045096 | E1BTF4_CHICK  G1MYK6_MELGA  11321/8047 | 64.0  56.0  64 | 1.5e-93  2.0e-81  3e-99 | 24 | 2077  (all) | 5.60 | C,T,Q |
| **gi\|224045100** | Ovalbumin-like | OVAL_MELGA  OVAL_CHICK  OVAL_COTJA  25985/20852 | 69.4  68.1  65.8  74/69 | 1.6e-120  8.3e-118  1.6e-114  1e-36/46 | 40 | 14315  (all) | 35.20 | C,T,Q |
| **H0YVQ1**  **gi\|224045162** | Similar to leukocyte antigen 86 (LY86) | G1MXR9_MELGA  F1P4F3_CHICK  23368 | 80.6  79.4  81 | 4.4e-60  9.1e-60  4e-24 | 8 | 371  (all) | 0.72 | C,T,Q |
| **gi\|224045188**  **H0YW82** | Similar to adseverin (SCIN) | ADSV_CHICK  G1NC77_MELGA  7222 | 92.7  92.2  83 | 0e0  0e0  0e0 | 9 | 71  (all) | 0.01 |  |
| **H0YX57**  **gi\|224045262** | Similar to coiled-coil domain-containing protein 126 (CCDC126) | G1NDK8_MELGA  E1BS45_CHICK  24241 | 92.1  92.1  90 | 1.7e-51  1.7e-51  7e-52 | 2 | 9  (Z1,3,3b) | 0.01 |  |
| **H0ZET4**  **gi\|224045955** | Endoplasmic reticulum resident protein 44 (ERP44) | G1N6U7_MELGA  E1BSL7_CHICK  12819 | 96.4  97.2  97 | 1.9e-162  2.8e-162  7e-72 | 5 | 30  (all) | <0.01 | C,T |
| **H0ZLL3**  **gi\|449494168** | Syntenin-1 (SDCBP) | Q5ZHM8_CHICK  G1NEY6_MELGA  24420 | 93.9  90.3  92 | 1.6e-107  3.0e-89  5e-54 | 6 | 153  (all) | 0.13 | C,T,Q |
| **gi\|449494396**  **H0ZNK9** | Calbindin (CALB1) | CALB1_CHICK  G1NH64_MELGA  24585 | 97.3  97.1  98 | 5.2e-98  2.9e-86  6e-57 | 10 | 98  (all) | 0.05 | C,T,Q |
| **gi\|224046577**  **H0ZPB2** | 14-3-3 protein zeta (YWHAZ); shares 3 with other 14-3-3 proteins | G1NI29_MELGA  1433Z_CHICK  20075 | 99.6  100.0  100 | 1.3e-95  3.3e-95  2e-79 | 13 | 105  (all) | 0.06 | C,T,Q |
| **H0ZR58**  **gi\|224046792** | Similar to prostate stem cell antigen (PSCA) | F1NXM7_CHICK  G1NJY7_MELGA  20430 | 73.1  73.1  69 | 3.3e-34  1.4e-33  8e-41 | 2 | 6  (Z1,3,3b) | 0.01 | C,T,Q |
| **H0YVG5**  **gi\|449495521** | Similar to UDP-GlcNAc:β-Gal β-1,3-N-acetylglucosaminyltransferase 2 (B3GNT2) | Q5ZK57_CHICK  G1NRV7_MELGA  6642 | 90.4  90.7  90 | 5.5e-168  2.0e-169  0e0 | 4 | 18  (Z1,3b) | 0.01 | C,T,Q |
| **H0Z947**  **gi\|224047478** | EGF-containing fibulin-like extracellular matrix protein 1 (EFEMP1)/Fibulin-5 | E1C6M8_CHICK  G1MSX1_MELGA | 98.2  98.4 | 4.9e-171  8.5e-121 | 13 | 240  (all) | 0.14 | C,T |
| **H0ZA92**  **gi\|224047508** | Similar to endoplasmic reticulum lectin 1 (ERLEC1) | F1NCV8_CHICK  G1MUM9_MELGA | 91.8  94.4 | 5.7e-208  1.0e-177 | 7 | 73  (all) | 0.02 | C,T |
| **gi\|224047800**  **H0ZI58** | Similar to angiotensinogen (AGT) | G1NHN9_MELGA  F1NDH2_CHICK  10880 | 67.8  67.8  67 | 4.0e-131  8.5e-130  1e-93 | 16 | 255  (all) | 0.12 | C,T,Q |
| **gi\|224048112**  **H0ZML2** | Similar to plasminogen (LPA-2) | F1NWX6_CHICK  G1NIF9_MELGA | 84.1  85.1 | 0e0  0e0 | 44 | 927  (all) | 0.53 | T |
| **H0ZMX2**  **gi\|224048143** | Similar to pantetheinase | E1BUA6_CHICK  G1NKC5_MELGA  6577 | 82.6  81.4  80 | 1.6e-185  6.5e-183  0e0 | 8 | 108  (all) | 0.05 | C,T,Q |
| **H0ZNK5**  **gi\|224048215** | Similar to acid sphingomyelinase-like phosphodiesterase 3a (SMPDL3A) | G1NKS2_MELGA  E1C6B2_CHICK | 90.0  90.1 | 1.2e-172  1.4e-172 | 2 | 15  (Z1,2,3b) | <0.01 |  |
| **H0ZNV1**  **gi\|224048253** | Similar to tyrosine-protein kinase receptor/ Proto-oncogene tyrosine-protein kinase (ROS1) | G1NKX7_MELGA  F1NQL9_CHICK  94 | 78.6  78.6  78 | 0e0  0e0  0e0 | 20 | 201  (all) | 0.01 | C,T,Q |
| **H0ZNY7**  **gi\|224048279** | Collagen α-1(X) chain (COL10A1), all peptides from non-triple helical domains; shares 1 peptide with gi\|449489078 (COL8A2) | F1NRH2_CHICK  G1NKZ8_MELGA  3922 | 89.2  88.9  87 | 7.9e-122  2.3e-95  0e0 | 12 | 226  (all) | 0.10 | C,T,Q |
| **H0ZR08**  **gi\|224048543** | Elongation factor 1-α (EEF1A1) | G1NM38_MELGA  EF1A_CHICK  4629/5279 | 99.4  99.4  89 | 7.8e-119  7.8e-119  0e0 | 9 | 131  (all) | 0.04 | C,T,Q |
| **H0ZS28**  **gi\|224048717** | 14-3-3 protein theta (YWHAQ); shares 3 peptides with H0Z4W0 | 1433T_CHICK  G1NMK1_MELGA  26318/26068 | 100.0  100.0  100 | 1.9e-101  1.9e-101  3e-51 | 6 | 62  (all) | 0.03 | C,T,Q |
| **H0YUM4**  **gi\|224049201** | Annexin (ANXA5) | F1NJI0_CHICK  G1NDY6_MELGA  11133 | 95.3  94.3  77 | 1.2e-123  1.2e-122  8e-88 | 7 | 66  (all) | 0.01 | C,T,Q |
| **gi\|224049274**  **H0YVI0** | Similar to ovocleidin-116 | OC116_CHICK  G1N6E1_MELGA  2298 | 34.2  34.5  32 | 4.5e-9  2.3e-7  7e-75 | 66 | 6498  (all) | 8.37 | C,T,Q |
| **gi\|224049282**  **H0YVK4** | Similar to ADP-ribose pyrophosphatase, mitochondrial (NUDT9) | G1N6G8_MELGA  F1NSM1_CHICK  21236/20048 | 85.0  84.4  92 | 2.4e-112  2.0e-111  4e-73 | 6 | 52  (all) | 0.02 | C,T,Q |
| **H0Z4U0**  **gi\|224049669** | Similar to cathepsin O (CTSO) | G1MS56_MELGA  Q5ZMK0_CHICK | 90.6  89.2 | 1.9e-117  2.6e-115 | 4 | 41  (Z1,2,3b) | 0.04 | C,T |
| **H0Z4V8**  **gi\|224049671** | Similar to platelet-derived growth factor C (PDGFC) | PDGFC_CHICK  G1MS78_MELGA  26854 | 93.9  93.9  95 | 1.0e-144  3.2e-131  6e-33 | 8 | 144  (all) | 0.05 | T,Q |
| **H0Z7S4**  **gi\|224049762** | Similar to vascular endothelial growth factor C (VEGFC) | E1C7C6_CHICK  G1N5F9_MELGA | 93.2  94.6 | 3.9e-168  3.3e-154 | 2 | 10  (Z1,3b) | <0.01 | T |
| **H0Z9P7**  **gi\|224049828** | Similar to acid ceramidase (ASAH1) | R4GH41_CHICK  G1NG36_MELGA | 90.1  91.9 | 2.1e-172  6.8e-166 | 11 | 136  (all) | 0.09 | C,T |
| **H0ZG96**  **gi\|224050039** | Similar to sodium-dependent phosphate transport protein 2B (SLC34A2) | F6T808_CHICK  G1NIK2_MELGA  2856 | 93.0  93.0  93 | 0e0  0e0  0e0 | 3 | 44  (all) | 0.01 | C |
| **H0ZGM0**  **gi\|224050051** | Similar to (extracellular) superoxide dismutase [Cu-Zn] (SOD3) | G1NIN0_MELGA  F1NHI4_CHICK  11203 | 90.1  87.7  91 | 6.8e-74  1.6e-71  6e-89 | 3 | 12  (Z1,3b) | <0.01 | T |
| **H0ZHW3**  **gi\|224050102** | Heparan sulfate glucosamine 3-O-sulfotransferase 1 (HS3ST1) | E1C4I5_CHICK  G3UQ78_MELGA  9140 | 96.2  90.7  93 | 1.6e-134  2.8e-62  1e-172 | 5 | 19  (Z1,3b) | 0.01 | C,T,Q |
| **H0ZDU9**  **gi\|224050809** | Calcitonin gene-related peptide 2 (CALCB)/procalcitonin | G1N4N0_MELGA  B0FYW9_CHICK  18915 | 95.7  94.9  95 | 5.9e-56  1.1e-55  1e-71 | 6 | 190  (all) | 0.37 | Q |
| **gi\|224050848**  **H0ZF34** | Similar to lactate dehydrogenase (LDHA) | LDHA_CHICK  G1N679_MELGA  18757 | 92.8  92.2  91 | 1.9e-132  2.8e-131  2e-84 | 10 | 93  (all) | 0.04 | C,T,Q |
| **H0ZGM2**  **gi\|224050910** | Similar to cathepsin D (CTSD) | CATD_CHICK  G1N8P0_MELGA  3793/15278 | 78.3  76.7  71 | 1.3e-142  3.9e-139  1e-168 | 17 | 477  (all) | 0.50 | C,T,Q |
| **H0ZJZ8**  **gi\|224051089** | Similar to prothrombin (F2) | G1NEM6_MELGA  F1NXV6_CHICK  3477 | 83.9  83.4  82 | 0e0  0e0  0e0 | 10 | 52  (all) | 0.02 | C,T,Q |
| **H0ZKB2**  **gi\|224051108** | Glycosyltransferase-like LARGE2 (GYLTL1B) | F1NX24_CHICK  G3USW8_MELGA  2336 | 95.8  93.0  95 | 0e0  0e0  0e0 | 2 | 5  (Z1,3b) | <0.01 | C,T |
| **H0ZKM9**  **gi\|224051141** | Similar to calcineurin B homologous protein 1 (CHP1) | CHP1_CHICK  G1NFA6_MELGA  18410 | 93.8  96.2  73 | 1.8e-70  1.3e-68  1e-70 | 5 | 37  (all) | 0.01 |  |
| **H0ZNH2**  **gi\|224051372** | Similar to actin, α cardiac muscle 1 (ACTC1); shares 11 peptides with H0Z0Q3 and 9 with H0Z125 | ACTC_CHICK  G1NIB0_MELGA  7084 | 100.0  100.0  94 | 8.6e-164  8.6e-164  0e0 | 3 | 49  (all) | 0.03 | C |
| **H0ZQS5**  **gi\|224051643** | Similar to legumain (LGMN) | E1C958_CHICK  G1NKC8_MELGA  21557/9286 | 90.5  81.3  92 | 1.6e-175  2.1e-154  4e-71 | 7 | 51  (all) | 0.01 | C,T,Q |
| **H0ZQY2**  **gi\|224051673** | Similar to α-1-antiproteinase 2 (SERPINA12-1/SerpinA4) | E1BS56_CHICK  G1NKH5_MELGA  1506 | 79.9  79.1  75 | 7.0e-129  1.1e-127  1e-168 | 8 | 55  (all) | 0.01 | T,Q |
| **H1A503**  **gi\|224051675** | Similar to α-1-antiproteinase 2 (SERPINA1) | G1NKH9_MELGA  E1C7T1_CHICK  6024 | 62.5  63.0  64 | 1.9e-104  3.5e-104  1e-143 | 10 | 71  (all) | 0.03 | C,T,Q |
| **H0ZRE5**  **gi\|224051739** | Heat shock protein HSP90-α (HSP90AA1); shares 5 peptides with gi\|449496679 | G1NKX1_MELGA  HS90A_CHICK  1525 | 98.9  98.8  98 | 7.3e-197  2.2e-196  0e0 | 22 | 309  (all) | 0.07 | C,T,Q |
| **H0ZSA4**  **gi\|224051968** | Similar to ERO1-like α (ERO1L); shares 1 peptide with H0ZJE2 (ERO1LB) | E1C0W7_CHICK  G1NM09_MELGA  14501 | 89.9  83.7  93 | 5.3e-192  1.2e-151  1e-113 | 2 | 5  (Z3b) | <0.01 |  |
| **gi\|224052234**  **H0Z3R0** | Similar to multiple inositol polyphosphate phosphatase 1 (MINPP1) | F1NPQ2_CHICK  G1MS05_MELGA  16370/21856 | 79.3  83.1  85 | 3.4e-100  2.0e-92  4e-68 | 19 | 441  (all) | 0.43 | C,T,Q |
| **gi\|224052361**  **H0Z7J3** | Similar to annexin A11(ANXA11); shares 1 peptide with B5FXE8/H0Z049 | F6SU00_CHICK  G1MRM3_MELGA  1989 | 87.6  77.8  66 | 9.1e-80  1.9e-53  6e-62 | 11 | 82  (all) | 0.01 | C,T,Q |
| **H0YXN8**  **gi\|224053915** | Aminoimidazole-4-carboxamide ribonucleotidetransformylase/IMP cyclohydrolase (PURH/ATIC) | Q5XKY5_CHICK  G1MWQ1_MELGA  7800/6182/15838 | 93.2  93.0  66 | 0e0  0e0  1e-136 | 7 | 35  (Z1,3b) | 0.01 |  |
| **H0ZAB5**  **gi\|224054950** | Polypeptide N-acetylgalactosaminyltransferase (GALNT3) | E1C241_CHICK  G1NE91_MELGA  15232/16021 | 94.1  93.8  93 | 0e0  0e0  3e-94 | 4 | 15  (Z1,2,3b) | <0.01 | C,T,Q |
| **gi\|224055990**  **H0ZM41** | Secreted frizzled-related protein 3 (FRZB) | SFRP3_CHICK  G1MYQ7_MELGA  24774/25330 | 95.0  96.1  96 | 6.3e-128  9.5e-52  1e-44 | 6 | 126  (all) | 0.11 | T |
| **gi\|224056071**  **H0ZN82** | α-1,6-mannosylglycoprotein 6-beta-N-acetylglucosaminyltransferase A (MGAT5) | F1NEL5_CHICK  G1NII7_MELGA  21418 | 98.2  98.7  97 | 0e0  0e0  6e-72 | 10 | 76  (all) | 0.01 | C,T,Q |
| **H0Z2Y3**  **gi\|224057270** | α-amylase (AMY1A) | F1NF53_CHICK  G1N3Q5_MELGA  8741/13022/17747/  10098 | 86.8  85.9  80 | 4.5e-194  8.9e-193  1e-167 | 5 | 41  (all) | 0.01 | C,T,Q |
| **gi\|224057264** | Pancreatic alpha-amylase-like; shares 3 peptides with H0Z2Y3 | F1NW02_CHICK  G1N3Q5_MELGA  8741/13022 | 82.0  81.4  75 | 3.5e-211  5.3e-209  1e-162 | 2 | 4  (Z1,3b) | <0.01 | C,T,Q |
| **H0Z3S3**  **gi\|224057306** | Similar to exostosin-like protein 2 (EXTL2) | R4GMG4_CHICK  G1N4T8_MELGA | 87.5  93.0 | 1.0e-134  8.7e-79 | 8 | 75  (all) | 0.06 | C |
| **gi\|224057610**  **H0Z847** | Similar to vitellogenin-2 (VIT2) | VIT2_CHICK  G5E7Q4_MELGA  Q9PUB0_COTJA  205 | 78.9  78.0  81.8  78 | 0e0  0e0  5.6e-57  0e0 | 104 | 4651  (all) | 1.22 | C,T,Q |
| **gi\|224057612** | Vitellogenin-2-like; shares 2 peptides with H0Z858 (CTBS) | E1BYN5_CHICK  G1NAD5_MELGA  1870 | 72.2  66.6  71 | 0e0  0e0  1e-124 | 55 | 964  (all) | 0.10 | C,T,Q |
| **gi\|224057626**  **H0Z889** | Similar to deoxyribonuclease-2-β (DNASE2B) | Q2XP49_CHICK  G1NAH7_MELGA  9668 | 79.2  78.2  80 | 6.5e-133  1.2e-130  2e-75 | 6 | 62  (all) | 0.03 | C,Q |
| **H0Z8U7**  **gi\|224057648** | Similar to vitellogenin-1 (TPRXL) | VIT1_CHICK  G1NAV2_MELGA  188 | 80.6  80.1  79 | 5.7e-193  1.4e-187  0e0 | 97 | 3549  (all) | 1.08 | C,T,Q |
| **H0ZCK8**  **gi\|224058069** | Similar to riboflavin-binding protein | G1NDJ8_MELGA  RBP_CHICK  B9A8Q3_COTJA | 82.2  81.3  78.0 | 7.8e-90  3.3e-89  5.1e-85 | 9 | 139  (all) | 0.11 | C,T,Q |
| **H0ZD13**  **gi\|224058079** | Protein O-linked-mannose β-1,2-N-acetylglucosaminyltransferase 1 (POMGNT1) | E1C532_CHICK  G1NDR4_MELGA  10369 | 95.9  95.8  90 | 0e0  0e0  1e-137 | 6 | 19  (Z2,3b) | <0.01 | C,T |
| **gi\|224058351**  **H0ZHW1** | Similar to angiopoietin-related protein 3 (ANGPTL3) | B1B565_CHICK  G1NGR5_MELGA  8710/6951 | 75.7  75.6  81 | 3.7e-103  3.0e-67  1e-125 | 16 | 532  (all) | 0.39 | C |
| **gi\|224060004**  **H0ZCA5** | Similar to glia-derived nexin (SERPINE2) | G1N0D9_MELGA  E1BWU2_CHICK  6327 | 95.5  86.0  84 | 2.1e-165  1.6e-128  1e-177 | 17 | 294  (all) | 0.20 | C,T,Q |
| **H0ZFA1**  **gi\|224060506** | Similar to apolipoprotein D (APOD) | G1N591_MELGA  Q5G8Y9_CHICK  28649 | 84.7  83.1  80 | 5.0e-78  5.6e-77  1e-35 | 5 | 114  (all) | 0.05 | C,T,Q |
| **H0ZGP1**  **gi\|224060593** | Similar to mannan-binding lectin serine protease 1 (MASP1) | F1N9E1_CHICK  G1N6P6_MELGA  2402 | 95.1  93.4  94 | 0e0  0e0  0e0 | 14 | 120  (all) | 0.05 |  |
| **H0ZJM5**  **gi\|224060745** | Eukaryotic initiation factor 4A-II (EIF4A2) | IF4A2_CHICK  G1NAW6_MELGA  7583 | 99.5  94.5  98 | 8.5e-151  4.1e-137  0e0 | 9 | 62  (all) | 0.02 | C,T,Q |
| **gi\|224060935**  **H0ZLM2** | Protein phosphatase 1L (PPM1L) | G1NDI2_MELGA  E1BTL4_CHICK  17452/24040 | 97.8  96.1  94 | 9.0e-93  8.4e-92  4e-71 | 7 | 51  (all) | 0.01 | C,T,Q |
| **H0ZMH0**  **gi\|224061188** | Similar to ceruloplasmin (CP) | F1N9R5_CHICK  G1NEW3_MELGA  12147/2749 | 83.1  82.9  85 | 0e0  0e0  3e-98 | 6 | 26  (Z1,3b) | <0.01 | T,Q |
| **H0Z0Q3**  **gi\|224061779** | Actin, cytoplasmic type 5; shares 14 peptides with H0Z125 and 12 with H0ZNH2 | ACT5_CHICK  G1NS52_MELGA  7084 | 99.7  99.7  99 | 2.2e-163  2.2e-163  0e0 | 18 | 895  (all) | 1.05 | C,Q |
| **gi\|224063020**  **H0ZIR1** | Similar to furin (FURIN) | Q91000_CHICK  G1NDT8_MELGA  2010/4372 | 85.2  74.8  81 | 0e0  2.3e-144  0e0 | 5 | 49  (all) | 0.01 | C,T,Q |
| **H0ZAJ9**  **gi\|224064295** | 72 kDa type IV collagenase (MMP2) | G1MZ17_MELGA  MMP2_CHICK  2942 | 96.2  96.2  96 | 0e0  0e0  0e0 | 18 | 232  (all) | 0.06 | C |
| **H0ZEP6**  **gi\|224064697** | Similar to T-cell immunomodulatory protein (ITFG1) | F1NWE2_CHICK  G1N0M3_MELGA  3546 | 89.4  89.2  89 | 0e0  0e0  0e0 | 5 | 21  (Z1,2,3b) | <0.01 | T |
| **H0ZH17**  **gi\|224064940** | Glucose-6-phosphate isomerase (GPI); shares 2 peptides with CON | F1NIJ6_CHICK  G1N314_MELGA  6110/18402 | 95.3  95.2  86 | 0e0  1.5e-193  5e-45 | 11 | 112  (all) | 0.02 |  |
| **H0ZHA8**  **gi\|224064949** | Similar to fatty acyl-CoA hydrolase, medium chain/carboxylesterase (CES1) | F1NYT3_CHICK  21184/19722/17274 | 78.6  79 | 3.0e-203  3e-49 | 5 | 32  (Z1,2,3b) | <0.01 | Q |
| **H0Z5T4**  **gi\|224065927** | Similar to glycosyltransferase 8 domain-containing protein 1 (GLT8D1) | E1BX23_CHICK  G1MQ81_MELGA  7174 | 89.2  88.5  85 | 3.5e-152  7.4e-134  0e0 | 2 | 9  (Z1,3b) | <0.01 |  |
| **gi\|224066001** | Semaphorin-3F-like; shares 23 peptides with H0Z7X2 | H9L024_CHICK  G1MYJ4_MELGA  9592/18183 | 97.4  97.2  79 | 0e0  0e0  1e-143 | 26 | 501  (all) | 0.21* | T |
| **H0Z7X2** | Similar to semaphorin-3F (SEMA3F), shares 23 peptides with gi\|224066001 | G1MYJ4_MELGA  H9L251_CHICK | 92.8  93.0 | 2.6e-190  2.7e-190 | 1 | 10  (Z1,2,3b) |  |  |
| **H0YQM2**  **gi\|224067576** | Uncharacterized protein; domain: Kazal (aa26-82) |  |  |  | 2 | 13  (Z1,3,3b) | 0.02 |  |
| **gi\|224067663**  **H0YR83** | SPARC | F1P291_CHICK  G1N2Z5_MELGA  SPRC_COTJA | 93.3  93.3  95.0 | 3.4e-125  8.9e-99  1.6e-124 | 7 | 52  (all) | 0.03 | C,T,Q |
| **H0YRC2**  **gi\|224067677** | Annexin (ANXA6) | F1NVG9_CHICK  G1N3X9_MELGA  2886 | 93.5  94.3  91 | 0e0  3.5e-211  0e0 | 10 | 53  (Z1,3,3b) | <0.01 | C,Q |
| **H0YSK2**  **gi\|224068038** | Similar to transforming growth factor-β-induced protein ig-h3 (TGFBI) | O42390_CHICK  G1N7Q8_MELGA  2800 | 93.1  94.2  93 | 0e0  0e0  0e0 | 14 | 201  (all) | 0.06 | C,T,Q |
| **gi\|224068202**  **H0YTF7** | Similar to SLIT3; shares 3 peptides with H0ZH52 (SLIT2) | F1NF14_CHICK  G3UNZ8_MELGA  350 | 95.9  92.9  94 | 0e0  0e0  0e0 | 16 | 65  (all) | <0.01 | Q |
| **gi\|224070374**  **H0ZCR4** | β-1,3-N-acetylglucosaminyltransferase lunatic fringe (LFNG) | G1MUY6_MELGA  F1NZ16_CHICK  11943 | 98.6  98.6  98 | 7.2e-98  2.9e-97  1e-113 | 5 | 36  (Z1,3b) | 0.02 | Q |
| **H0ZCS3**  **gi\|224070382** | Carbohydrate sulfotransferase 12 (CHST12) | E1BR90_CHICK  G1NR95_MELGA  5910 | 95.0  94.7  94 | 6.2e-185  2.3e-184  0e0 | 14 | 249  (all) | 0.10 | C,T,Q |
| **H0ZEX5**  **gi\|224070509** | Ras-related C3 botulinum toxin substrate 1 (RAC1) | G1N850_MELGA  F1N8D9_CHICK  30108/18289 | 100.0  100.0  98 | 6.1e-76  1.3e-75  1e-26 | 5 | 31  (all) | 0.04 | C,T,Q |
| **H0YVQ5**  **gi\|224072899** | Uncharacterized protein (DPP7)/similar to dipeptidyl peptidase 2 | E1BZ81_CHICK  G1MQD8_MELGA  13115 | 86.6  83.4  84 | 3.0e-170  4.0e-160  1e-108 | 7 | 35  (Z1,3b) | 0.02 | C,T,Q |
| **gi\|224072995**  **H0YWL2** | Similar to δ-aminolevulinic acid dehydratase (ALAD) | G1MSP0_MELGA | 93.8 | 5.5e-104 | 2 | 9  (Z1,3b) | 0.01 |  |
| **H0Z1Z0**  **gi\|224073506** | Similar to torsin-1B (TOR1B) | F1NWW1_CHICK  G1N0L0_MELGA  16016/10652 | 87.7  87.4  88 | 1.8e-123  4.8e-123  4e-94 | 7 | 38  (all) | 0.01 | C,T,Q |
| **gi\|224073965**  **H0ZAB3** | 78 kDa glucose-regulated protein (HSPA5); shares 2 peptides with H0YQE7 (HSPA8) | GRP78_CHICK  G1N8R5_MELGA  A0PA15_COTJA/  3436 | 98.6  99.8  98.6 | 0e0  0e0  0e0 | 24 | 465  (all) | 0.08 | C,T,Q |
| **H0YY18**  **gi\|224074383** | Similar to N-sulfoglucosamine sulfohydrolase (SGSH) | F1NGI6_CHICK  G1N333_MELGA  11716 | 90.0  66.5  91 | 6.4e-198  1.1e-79  1e-102 | 9 | 45  (Z1,3,3b) | 0.01 | T,Q |
| **H0YZ76**  **gi\|224074434** | Rho GDP-dissociation inhibitor 1 (ARHGDIA) | G1N1C7_MELGA  F1P3P3_CHICK  14877 | 98.3  98.3  98 | 6.2e-77  6.2e-77  1e-100 | 2 | 3  (Z1,3b) | <0.01 | C,T,Q |
| **gi\|224074517**  **H0Z1D4** | Similar to β-2-glycoprotein 1 (APOH) | G1N5Y2_MELGA  F1NYG4_CHICK  16433 | 76.7  77.4  54 | 6.6e-118  1.0e-117  5e-36 | 4 | 21  (Z1,3,3b) | 0.02 | C,T,Q |
| **H0ZB43**  **gi\|224074837** | Meteorin-like (METRNL) | R4GHN2_CHICK  G1MWQ7_MELGA  19142/24425 | 96.1  95.3  94 | 3.4e-115  4.8e-115  5e-69 | 6 | 30  (Z1,2,3b) | 0.02 | C,T,Q |
| **gi\|224075483**  **H0ZZS7** | Similar to metalloproteinase inhibitor 2 (TIMP2) | R4GIL5_CHICK  G3UTJ0_MELGA  20284 | 94.3  92.5  94 | 3.5e-48  2.4e-46  1e-57 | 2 | 7  (Z1,3,3b) | 0.03 | C,T,Q |
| **gi\|224076322**  **H0Z519** | Unconventional myosin-I (MYO1C) | F1NG39_CHICK  G1N0G6_MELGA  1056 | 96.2  92.3  96 | 0e0  0e0  0e0 | 12 | 59  (all) | <0.01 | C,T,Q |
| **H0Z6B5**  **gi\|224076380** | Similar to vitronectin (VTN) | O12945_CHICK  G1N1Y6_MELGA  4894/6811 | 75.8  75.5  69 | 3.1e-111  1.4e-109  0e0 | 10 | 40  (all) | 0.02 | C,T |
| **gi\|224078167**  **H0Z7K8** | Similar to BPI fold-containing family B member 4/ovocalyxin-36 | Q53HW8_CHICK  G1N6M8_MELGA  21806 | 57.6  58.3  61 | 4.5e-114  7.3e-114  5e-41 | 12 | 1048  (all) | 1.59 | C,T,Q |
| **gi\|224078299**  **H0Z9P3** | Similar to tumor necrosis factor receptor superfamily member 6B (TNFRSF6B) | G1N4U8_MELGA  F1NCY6_CHICK  19295 | 76.4  75.6  41 | 2.0e-42  2.1e-42  3e-29 | 6 | 147  (all) | 0.38 | C,T,Q |
| **H0YW24**  **gi\|224079514** | Similar to procollagen-lysine,2-oxoglutarate 5-dioxygenase 1 (PLOD1) | PLOD1_CHICK  G1N380_MELGA  2406 | 92.9  92.9  92 | 0e0  0e0  0e0 | 11 | 129  (all) | 0.02 | C,T,Q |
| **H0Z0D8**  **gi\|224079993** | α-enolase(ENO1); shares 3 peptides with H0ZSU0 (ENO2) | F1NZ78_CHICK  G1MX98_MELGA  7773 | 96.8  95.1  95 | 7.0e-170  4.0e-168  0e0 | 17 | 380  (all) | 0.16 | C,T,Q |
| **H0Z0U7**  **gi\|224080053** | Similar to 45 kDa calcium-binding protein (SDF4) | CAB45_CHICK  G1MW20_MELGA  7982/15194 | 86.2  86.2  83 | 2.1e-129  2.1e-129  1e-154 | 18 | 378  (all) | 0.26 | C,T,Q |
| **gi\|449487178**  **H1A3R7** | Similar to ras-like protein Cdc42a (CDC42) | G1N4S3_MELGA  CDC42_CHICK  17386 | 100.0  99.4  100 | 1.4e-69  2.6e-69  3e-92 | 4 | 13  (Z1,3b) | 0.01 | C,T,Q |
| **H0YSH0**  **gi\|224081746** | Similar to tissue α-L-fucosidase (FUCA1) | F1P4X3_CHICK  G1N260_MELGA  4244/10646 | 83.6  83.3  86 | 4.0e-177  1.6e-147  1e-159 | 10 | 120  (all) | 0.06 | C,T,Q |
| **H0YQ63**  **gi\|224083284** | Similar to out at first protein homolog (OAF) | OAF_CHICK  G1MTX0_MELGA  15505 | 93.2  88.7  94 | 3.2e-69  2.2e-63  2e-62 | 4 | 93  (all) | 0.11 | C,T,Q |
| **gi\|224083318**  **H0YQE7** | Similar to heat shock cognate 71 kDa protein (HSPA8); shares 2 peptides with H0ZAB3 (HSPA5) | F1NWP3_CHICK  G1MSW3_MELGA  A0PA16_COTJA | 99.8  99.8  99.8 | 0e0  0e0  0e0 | 28 | 612  (all) | 0.22 | C,T,Q |
| **H0YQL6**  **gi\|449489503** | Similar to amyloid-like protein 2 (APLP2); shares 1 peptide with H0ZTL2 (APP) | F1P0A7_CHICK  G1MQW1_MELGA  2239 | 92.8  92.8  92 | 2.1e-190  2.4e-190  0e0 | 8 | 75  (Z1,3,3b) | 0.01 | C,T,Q |
| **gi\|449489729**  **H0Z037** | Similar to tropomyosin α-1 chain (TPM3) | G5E7W7_MELGA  H9L074_CHICK  18026 | 94.7  97.4  85 | 7.4e-46  1.4e-44  3e-72 | 4 | 14  (Z1,3b) | 0.01 | T |
| **gi\|224085833**  **H1A4K1** | F-actin-capping protein subunit α-1 (CAPZA1) | CAZA1_CHICK  G1N026_MELGA  29694 | 97.9  98.6  98 | 5.2e-121  3.5e-118  5e-32 | 2 | 23  (all) | 0.01 |  |
| **gi\|224086482** | Keratin type I 19-like; shares 10 peptides with CON | F1NDN9_CHICK  G1MVR3_MELGA  11974 | 88.1  84.5  90 | 1.0e-96  9.1e-92  1e-122 | 8 | 76  (all) | 0.01 | C,T,Q |
| **H0ZG62**  **gi\|224087229** | Similar to RAB11A | RB11A_CHICK  G1NBU1_MELGA  21797/20214 | 99.1  100.0  98 | 3.9e-74  4.0e-69  3e-39 | 3 | 14  (Z1,3b) | 0.01 | C,T,Q |
| **H0YPL5**  **gi\|224088482** | Uncharacterized protein/ α-L-iduronidase (IDUA) | Q5F366_CHICK  G1N693_MELGA  3233 | 85.1  77.1  85 | 0e0  3.2e-212  0e0 | 16 | 202  (all) | 0.04 | T,Q |
| **A2CIZ3**  **gi\|91805309** | ATP synthase subunit α (ATP5A1) | F1NI22_CHICK  G1NC68_MELGA  1976 | 97.5  96.9  98 | 3.1e-63  2.9e-62  1e-103 | 3 | 5  (Z1,3b) | <0.01 | C,T,Q |
| **H0YUD8**  **gi\|224090240** | Similar to avidin | G1MSZ9_MELGA  AVID_CHICK  29257 | 68.4  67.3  76 | 7.5e-41  9.0e-41  3e-14 | 2 | 59  (all) | 0.30 | C,T,Q |
| **H0YXU7**  **gi\|224090725** | Similar to Golgi membrane protein 1 (GOLM1) | E1C3N9_CHICK  20862 | 68.1  53 | 3.2e-62  1e-34 | 4 | 86  (all) | 0.02 | C,Q |
| **H0YYZ8**  **gi\|224090855** | Guanine nucleotide-binding protein G(q) subunit α (GNAQ); shares 1 peptide with H0YQK2 (GNA11) | Q5F3B5_CHICK  GNA11_MELGA  13584 | 98.3  89.7  75 | 9.5e-151  2.2e-139  2e-68 | 1 | 18  (all) | <0.01 | C |
| **H0Z6E7**  **gi\|224091413** | Similar to β-hexosaminidase subunit beta (HEXB) | F1NTQ2_CHICK  G1MRL4_MELGA  14128 | 84.1  69.2  83 | 3.8e-201  1.2e-122  1e-108 | 18 | 376  (all) | 0.16 | C,T,Q |
| **H0YWL5**  **gi\|224092982** | Similar to semaphorin-3C (SEMA3C) | F1P3L3_CHICK  G3UPZ0_MELGA  2257 | 96.3  96.0  95 | 0e0  0e0  0e0 | 6 | 44  (all) | 0.01 | C,T,Q |
| **H0ZGF0**  **gi\|449481700** | Histone H2B (H2B-V, H2B-I, H2B-VII, H2B-VIII) | H2B5_CHICK  G1NRI6_MELGA  18290/17702 | 100.0  99.2  99 | 5.3e-46  8.6e-46  3e-66 | 5 | 21  (all) | 0.04 | Q |
| **H0Z920**  **gi\|224094017** | Similar to carboxypeptidase M (CPM) | E1C041_CHICK  G1NDL5_MELGA  12413 | 85.0  85.3  87 | 2.4e-172  8.5e-172  1e-131 | 14 | 247  (all) | 0.11 | C |
| **gi\|224094562**  **H0ZG82** | Histone H2A (H2AFJ) | H2AJ_CHICK  H9H207_MELGA  21053 | 100.0  92.1  97 | 3.6e-50  9.1e-46  1e-65 | 4 | 38  (all) | 0.09 | C,T,Q |
| **H0ZGS6**  **gi\|224094659** | Similar to phospholipase B (PLDB1) | G1NHA1_MELGA  E1BZF7_CHICK  10689 | 91.5  91.3  90 | 0e0  0e0  1e-147 | 6 | 27  (all) | 0.01 | C,T,Q |
| **H0ZHF7**  **gi\|224094705** | Similar to α-N-acetylgalactosaminidase (NAGA) | F1NJF8_CHICK  G3UPD1_MELGA  14035 | 84.7  80.3  87 | 2.4e-160  2.9e-145  1e-109 | 5 | 35  (all) | 0.01 | C,T |
| **H0ZLS8**  **gi\|224095440** | Heat shock protein 90kDa-β/endoplasmin (HSP90B1) | ENPL_CHICK  G1NK19_MELGA  1971 | 98.2  98.2  98 | 0e0  0e0  0e0 | 10 | 36  (all) | <0.01 | C,T,Q |
| **H0ZP74**  **gi\|449482107** | Protein kinase C and casein kinase substrate in neurons protein 2 (PACSIN2) | E1C007_CHICK  G1NLV8_MELGA  7566/10661 | 99.8  93.0  87 | 2.0e-159  2.2e-145  1e-140 | 4 | 23  (Z1,3b) | 0.01 | Q |
| **H1A593**  **gi\|224095998** | Similar to lactosylceramide 4-α-galactosyltransferase | E1C034_CHICK  G1NS03_MELGA  6347 | 71.5  70.6  70 | 6.5e-119  1.2e-117  1e-144 | 3 | 6  (Z1,3b) | <0.01 |  |
| **H0ZR45**  **gi\|224096470** | Similar to MANSC domain-containing protein 1 (MANSC1) | F1NZW5_CHICK  G3URN7_MELGA  24288 | 57.5  58.0  66 | 3.3e-71  7.1e-66  1e-31 | 3 | 16  (Z1,3b) | <0.01 | T |
| **H1A0U4**  **gi\|224096590** | Similar to calumenin (CALU) | E1BXF4_CHICK  11292 | 60.3  60 | 3.4e-25  3e-19 | 5 | 88  (all) | 0.07 | C |
| **gi\|224098158**  **H0Z2M6** | Similar to iduronate 2-sulfatase (IDS) | F1NFI0_CHICK  G1N835_MELGA  5974 | 84.7  83.5  82 | 2.2e-203  5.4e-202  1e-172 | 4 | 23  (Z1,3b) | <0.01 | Q |
| **H0Z6P7**  **gi\|224098392** | Phosphoglycerate kinase (PGK1) | F1NU17_CHICK  G1N3D8_MELGA  15260 | 97.1  96.1  96 | 2.9e-169  2.2e-160  1e-107 | 5 | 24  (Z1,2,3b) | <0.01 | C,T |
| **gi\|224136256**  **H1A3L5** | Similar to biotinidase; shares 7 peptides with H0YXX4/gi\|449492705 (BTD, Biotinidase isoform 2) | E1C3J7_CHICK  G1NED4_MELGA  6873 | 87.9  87.9  86 | 7.1e-195  7.1e-195  0e0 | 2 | 25  (all) | 0.01 | C,T,Q |
| **H0ZYD6**  **gi\|224147926** | Similar to ephrin-A3 (EFNA3) |  |  |  | 2 | 8  (Z1,3b) | <0.01 |  |
| **gi\|224169433**  **H0ZW92** | Ras-related protein rab-10 (RAB10) | F1NMT5_CHICK  G3UR98_MELGA  17613 | 88.4  88.4  100 | 2.7e-32  2.9e-32  3e-40 | 2 | 26  (Z1,3b) | 0.01 | C,T,Q |
| **gi\|224172798**  **H0ZZK5** | Similar to laminin subunit γ-1 (LAMC1); shares 4 peptides with H0Z0P2 | G1MSJ8_MELGA  Q90ZN3_CHICK  1462 | 87.5  85.8  82 | 0e0  0e0  0e0 | 17 | 143  (all) | 0.01 | C,T,Q |
| **H0Z0P2**  **gi\|224073548** | Similar to laminin subunit γ-3 (LAMC1); shares 4 peptides with H0ZZK5 | F1NI05_CHICK  G1MSJ8_MELGA  1133 | 84.7  83.8  84 | 0e0  0e0  1e-161 | 3 | 19  (all) | <0.01 | C,T,Q |
| **gi\|310750352**  **H0ZSY4** | Glyceraldehyde-3-phosphate dehydrogenase (GAPDH) | F1NH87_CHICK  G1NMR6_MELGA  971 | 98.8  98.8  82 | 2.4e-134  2.5e-134  1e-148 | 12 | 186  (all) | 0.10 | C,T,Q |
| **gi\|315113883**  **H0Z7F2** | 15 kDa selenoprotein (SEP15) | M9P293_MELGA  (IPI00571120) | 100.0 | 1.4e-22 | 3 | 34  (all) | 0.02 | C,T |
| **H0ZNW5**  **gi\|371940847** | Von Willebrand factor (VWF) | F5XVB5_CHICK  G1NM99_MELGA  18140 | 79.6  78.5  84 | 0e0  0e0  6e-85 | 42 | 409  (all) | 0.04 | T |
| **gi\|401461803**  **H0ZLL6** | Similar to thioredoxin reductase 1, cytoplasmic (TXNRD1) | G1NJW8_MELGA  F6S2M1_CHICK  5207 | 87.9  90.5  84 | 1.1e-187  1.2e-181  0e0 | 4 | 28  (all) | 0.01 | T |
| **gi\|401664564**  **H0YRF2** | Glutathione peroxidase 3 (GPX3) | G1N365_MELGA  F1NPJ8_CHICK  19686 | 88.3  87.5  81 | 4.7e-49  9.1e-49  6e-68 | 10 | 404  (all) | 1.06 | C,T,Q |
| **gi\|449470890**  **H0Z1U5** | Pyruvate kinase (PKM) | F1NW43_CHICK  G1MSA4_MELGA  7153 | 95.8  91.9  85 | 2.9e-214  2.5e-205  1e-173 | 21 | 345  (all) | 0.09 | C,T,Q |
| **H0Z4R0**  **gi\|449470945** | Annexin (ANXA2) | ANXA2_CHICK  G1MZD9_MELGA  16079/887 | 98.5  98.2  98 | 4.3e-131  1.2e-130  1e-105 | 15 | 204  (all) | 0.06 | C,T,Q |
| **H0Z6T0**  **gi\|449470999** | Disintegrin and metalloproteinase domain-containing protein 10 (ADAM10) | Q8QFX0_CHICK  G1N1F6_MELGA  2267/29154 | 93.7  94.0  93 | 0e0  0e0  0e0 | 5 | 26  (Z1,3b) | <0.01 | C,T,Q |
| **H0Z998**  **gi\|449471071** | Similar to secretogranin-3 (SCG3) | E1BSQ1_CHICK  G1N454_MELGA  7340/8898 | 92.3  93.1  83 | 4.6e-145  3.3e-141  1e-166 | 11 | 57  (Z1,3b) | 0.01 | C,T |
| **gi\|449471958**  **H0ZVB1** | Similar to β-hexosaminidase subunit alpha | F1NEX5_CHICK  G1MRV8_CHICK  7139/10068 | 86.0  84.9  86 | 9.4e-152  7.0e-151  0e0 | 12 | 113  (all) | 0.03 | C,T,Q |
| **gi\|449471458**  **H0Z606** | Similar to thrombospondin type-1 domain-containing protein 4 (THSD4) | F1NVQ5_CHICK  G1N0M4_MELGA  11387 | 88.3  93.8  93 | 0e0  0e0  1e-149 | 8 | 28  (Z1,3b) | <0.01 | C,T,Q |
| **H0ZD74**  **gi\|449471648** | Similar to lactadherin (MFGE8) | E1C0K5_CHICK  G1N944_MELGA  9870/11245 | 88.7  87.2  89 | 4.8e-132  1.8e-130  1e-107 | 15 | 278  (all) | 0.27 | C,T,Q |
| **H0ZD86**  **gi\|449471652** | Similar to hyaluronan and proteoglycan link protein 3 (HAPLN3) | G1N954_MELGA  E1BUN0_CHICK  15813 | 79.4  78.9  66 | 7.3e-136  2.5e-135  1e-63 | 15 | 220  (all) | 0.13 | C,T,Q |
| **H0ZDX6**  **gi\|449471681** | ST8 alpha-N-acetyl-neuraminide alpha-2,8-sialyltransferase 2 (ST8SIA2) | F1NIM6_CHICK  G3UPI4_MELGA  19840/26094/24323 | 96.2  95.9  97 | 1.7e-158  2.6e-145  3e-68 | 5 | 54  (all) | 0.02 |  |
| **H0ZFA7**  **gi\|449471729** | Similar to proprotein convertase subtilisin/kexin type 6 (PCSK6); shares 1 peptide with H0YR65 (PCSK5) | F1NA29_CHICK  G1NB15_MELGA  5733 | 90.7  88.1  74 | 0e0  1.6e-210  0e0 | 3 | 16  (Z1,3b) | <0.01 | C |
| **H0ZJI3**  **gi\|449471853** | Ras GTPase-activating-like protein IQGAP1 (IQGAP1) | G1NED9_MELGA  F1NY82_CHICK  4876 | 95.8  94.1  96 | 0e0  0e0  0e0 | 10 | 18  (Z1,2,3b) | <0.01 | T |
| **H0ZI75**  **gi\|449471885** | Similar to cathepsin H (CTSH) | F5CE71_CHICK  G1NDQ5_MELGA | 88.1  87.6 | 9.1e-89  1.0e-80 | 3 | 48  (all) | 0.04 | T |
| **gi\|449471913**  **H0ZIL0** | Similar to α-mannosidase 2x (MAN2A2) | F1NXU9_CHICK  G1NE21_MELGA  6255/4296 | 89.7  88.6  91 | 0e0  0e0  0e0 | 13 | 71  (all) | 0.01 | C,T,Q |
| **H0ZIZ6**  **gi\|449471931** | Similar to protein disulfide-isomerase A3 (PDIA3) | PDIA3_CHICK  G1NDL0_MELGA  8258/6729 | 93.5  94.4  92 | 1.9e-190  5.2e-165  0e0 | 17 | 121  (Z1,3,3b) | 0.02 | T,Q |
| **gi\|449471984**  **H0ZVJ4** | Similar to repulsive guidance molecule A (RGMA) | RGMA_CHICK  G1NA39_MELGA  11670 | 89.5  89.5  92 | 2.3e-93  2.6e-93  1e-114 | 6 | 76  (all) | 0.03 | C,T,Q |
| **H0Z8S1**  **gi\|449472570** | Membrane-bound transcription factor site-1 protease (MBTPS1) | E1C6Y2_CHICK  G1MQ93_MELGA  985 | 98.2  98.2  94 | 0e0  0e0  0e0 | 9 | 45  (Z1,3,3b) | 0.01 | T,Q |
| **H0Z7D7**  **gi\|449472689** | Golgi apparatus protein 1 (GLG1) | GSLG1_CHICK  G1MRQ6_MELGA  780 | 96.8  97.3  96 | 0e0  0e0  0e0 | 34 | 642  (all) | 0.10 | C,T,Q |
| **gi\|449473017**  **H0ZCH7** | Similar to cadherin-1 (CDH1) | E1C6M9_CHICK  G1N7A9_MELGA  1542/3257 | 73.5  70.2  74 | 6.7e-129  2.2e-124  0e0 | 5 | 32  (all) | <0.01 | C,T,Q |
| **H0ZDH8**  **gi\|449473062** | Similar to carbohydrate sulfotransferase 4 (CHST4); shares 1 peptide with H0ZDQ4 (CHST5) | E1C5G5_CHICK  G1NRE8_MELGA  7129 | 90.5  89.6  89 | 1.8e-144  1.9e-143  1e-179 | 2 | 3  (Z2,3b) | <0.01 |  |
| **H0Z021**  **gi\|449473355** | Similar to fibulin-2 (FBLN2) | F1NWN4_CHICK  G1N1D3_MELGA  5477/12336/10272 | 81.5  73.1  74 | 1.3e-136  3.1e-136  0e0 | 6 | 31  (all) | <0.01 | T |
| **gi\|449473387**  **H0Z1T8** | Plasma membrane calcium-transporting ATPase 2 (ATP2B2) | G1MZP2_MELGA  F1NFC7_CHICK  22389 | 97.7  95.7  100 | 0e0  0e0  1e-66 | 21 | 187  (all) | 0.02 | C,T,Q |
| **H0Z430**  **gi\|449473480** | Dystroglycan (DAG1) | R4GH71_CHICK  G1MW21_MELGA  4210 | 95.2  94.7  96 | 6.4e-212  1.8e-205  0e0 | 10 | 206  (all) | 0.07 | C,T,Q |
| **gi\|449473623**  **H0ZAR1** | Similar to filamin-B (FLNB) | F1N8D4_CHICK  G1N4G8_MELGA  71/63 | 92.6  93.7  93 | 0e0  0e0  0e0 | 18 | 30  (Z1,3b) | <0.01 | T |
| **gi\|449473789** | Extracellular matrix protein 2 (ECM2) | E1BV71_CHICK  G1MYY9_MELGA  5427 | 76.5  76.1  76 | 4.9e-127  1.1e-124  0e0 | 4 | 8  (Z1,3,3b) | <0.01 |  |
| **gi\|449473846**  **H0Z7F7** | Similar to semaphorin-3G (SEMA3G) | G1MXI6_MELGA  F1NQ93_CHICK  3281/621 | 84.2  83.9  68 | 2.0e-202  1.3e-201  1e-148 | 16 | 161  (all) | 0.03 | C,T,Q |
| **H0Z8V2**  **gi\|449473882** | Transketolase (TKT) | F1P1A5_CHICK  G1N238_MELGA  4394 | 96.4  96.1  88 | 0e0  0e0  0e0 | 6 | 12  (Z2,3b) | <0.01 | Q |
| **H0ZAB9**  **gi\|449473927** | ADP-ribosylation factor 4 (ARF4); shares 1 peptide with H0YPN5 (ARF1) | G1N3Z1_MELGA  Q5ZKR9_CHICK  16886/18449 | 98.1  95.2  95 | 1.0e-58  1.8e-58  8e-89 | 3 | 10  (Z1,3b) | <0.01 | C,T,Q |
| **H0YPX7**  **gi\|449474791** | Similar to stanniocalcin-2 (Fragment; STC2) | E1BRJ2_CHICK  G1MX28_MELGA  12122 | 91.2  91.2  91 | 2.1e-100  1.9e-100  1e-132 | 3 | 22  (Z1,3b) | 0.01 | C,Q |
| **gi\|449474881** | **aa~319-774** ovoinhibitor-like; shares 23 peptides with H0YQL7 | IOV7_CHICK  G1MZX1_MELGA  7445 | 71.2  66.1  71 | 7.0e-144  5.4e-130  1e-146 | 25 | 896  (all) | 0.53* | C,T,Q |
| **H0YQL7** | Uncharacterized protein/similar to ovoinhibitor; shares 23 peptides with gi\|449474881 | IOV7_CHICK  G1MZX1_MELGA  7445 | 73.2  66.8  71 | 5.0e-136  6.2e-120  1e-146 | 1 | 8  (Z1) |  |  |
| **gi\|449474987**  **H0ZVA7** | SPOCK-1/testican-1; shares 7 peptides with H0YSI2 | F1NW71_CHICK  G1N7F0_MELGA  18342/26927 | 94.1  97.9  86 | 1.1e-118  1.3e-92  2e-92 | 9 | 56  (all) | 0.01* |  |
| **H0YSI2** | Similar to testican-1(SPOCK1); shares 7 peptides with gi\|449474987 | F1NW71_CHICK  G1N7F0_MELGA  18342/26927 | 94.1  97.9  86 | 4.0e-119  5.8e-93  2e-92 | 1 | 12  (Z3b) |  |  |
| **H0YU07**  **gi\|449475124** | Similar to protocadherin gamma subfamily C, 3 (PCDHGC3) | R4GLV8_CHICK  G1MSV1_MELGA  1364 | 88.3  88.6  87 | 0e0  0e0  0e0 | 4 | 12  (Z1,3b) | <0.01 |  |
| **H0YST5**  **gi\|449475173** | Uncharacterized protein (FSTL4) | G1N8T8_MELGA  F1NWP0_CHICK  1775/19271 | 85.5  85.3  85 | 0e0  0e0  0e0 | 17 | 157  (all) | 0.03 | Q |
| **gi\|449475300** | Similar to LOC100229528/similar to protocadherin (β-1) | F1P1T8_CHICK  2597/5696/11390 | 73.6  71 | 0e0  0e0 | 4 | 16  (Z1,3b) | <0.01 |  |
| **gi\|449475308**  **H1A0M5** | Similar to protocadherin γ A3-like (PCDHGB5); shares 1 peptide with gi\|449475310 | R4GLV8_CHICK  G1MSV1_MELGA  11859 | 61.8  61.7  75 | 2.0e-128  2.1e-128  1e-107 | 4 | 19  (Z1,2,3b) | <0.01 |  |
| **gi\|449475310** | Similar to protocadherin γ-B3-like;  Shares 1 peptide with gi\|449475308 | F1P1T8_CHICK  G1MST6_MELGA  11859 | 48.2  46.9  75 | 8.9e-69  4.6e-65  1e-107 | 1 | 14  (Z1,2,3b) | <0.01 |  |
| **H0ZX95**  **gi\|449475325** | Similar to nucleotide exchange factor SIL1 (SIL1) | F1NWG7_CHICK  G1MRI3_MELGA  29976 | 83.4  82.1  93 | 9.4e-107  4.9e-105  1e-27 | 9 | 33  (all) | 0.01 | C,T,Q |
| **gi\|449475443**  **H0YYI9** | Similar to netrin-3 (NTN3); shares 1 peptide with H0Z423 (NTN1) | NET3_CHICK  G1NC79_MELGA  3495/12297/21722 | 90.2  73.9  66 | 0e0  6.3e-124  0e0 | 16 | 332  (all) | 0.15 |  |
| **H0YZD2**  **gi\|449475525** | Nucleoside diphosphate kinase (NME3) | R4GM98_CHICK  G1NBU7_MELGA  22926 | 80.5  84.1  83 | 5.8e-60  2.5e-56  5e-53 | 7 | 85  (all) | 0.05 | T,Q |
| **H0Z6D1**  **gi\|449475843** | Similar to amiloride-sensitive sodium channel subunit β (SCNN1B) | G1N4H6_MELGA  F1NE95_CHICK  8294/12995/9850/ 21965 | 88.4  87.7  84 | 6.4e-211  2.0e-210  1e-166 | 2 | 11  (Z1,3b) | <0.01 |  |
| **gi\|449476362**  **H0ZDY3** | Kinase FAM20C (FAM20C) | E1C4X0_CHICK  G1MSD1_MELGA  14673/15389 | 99.5  98.6  90 | 1.4e-175  5.1e-136  1e-127 | 10 | 184  (all) | 0.08 | C,T,Q |
| **H0ZE90**  **gi\|449476366** | Neuronal pentraxin-2 (NPTX2) | E1C7S1_CHICK  G1MS71_MELGA  14012/14364/15545 | 95.3  98.9  98 | 5.1e-147  4.9e-102  1e-127 | 5 | 61  (all) | 0.01 | C,T,Q |
| **H0ZGH7**  **gi\|449476458** | Similar to transmembrane channel-like 5 (TMC5) | G1N7F8_MELGA  F1NIK1_CHICK | 75.9  75.0 | 8.5e-197  1.2e-190 | 3 | 12  (Z1,3b) | 0.01 | C |
| **gi\|449476510** | Similar to mesothelin; shares 6 peptides with H0ZYM3; aa225-391 and 721-887 of this entry are completely identical! | G1N1K4_MELGA  F1NZV7_CHICK  2480/5817 | 76.6  74.9  50 | 8.2e-56  6.4e-55  1e-111 | 36 | 1505  (all) | 0.91 | C,T,Q |
| **H0ZYM3** | Similar to mesothelin; fragment shares 6 peptides with gi\|449476510 | F1NZV7_CHICK  G1N1K4_MELGA  13801/5817 | 65.7  63.4  63 | 6.0e-56  3.8e-43  1e-29 | 1 | 2  (Z2,3b) | <0.01 |  |
| **H0ZFH8**  **gi\|449477362** | Similar to phospholipase B domain containing 2 (PLBD2) | F1P0Z3_CHICK  G1NCL1_MELGA  20334/14737 | 78.4  80.3  75 | 2.4e-136  1.1e-110  2e-35 | 8 | 44  (all) | 0.01 | C,T,Q |
| **H0YVR2**  **gi\|449477715** | α-1,2-mannosidase (MAN1B1) | F1NGN6_CHICK  G1MQH0_MELGA  4371/13896 | 89.9  90.3  78 | 0e0  0e0  0e0 | 13 | 109  (all) | 0.02 | C,T |
| **gi\|449477748**  **H0YW11** | Uncharacterized protein/similar to extracellular fatty acid-binding protein | EXFAB_CHICK  G1MQ16_MELGA  EXFAB_COTJA | 56.1  44.2  45.6 | 1.1e-16  4.6e-15  1.9e-15 | 2 | 16  (Z1,3b) | 0.01 | C,T,Q |
| **H1A127**  **gi\|449477841** | Similar to arrestin domain-containing protein 1 (ARRDC1) | Q5ZKV5_CHICK  G1MTX5_MELGA  10776 | 95.1  94.2  94 | 1.0e-121  2.9e-121  5e-99 | 5 | 64  (all) | 0.03 | C,T,Q |
| **H0YY82**  **gi\|449477859** | Syntaxin-binding protein 1 (STXBP1) | G1MUZ5_MELGA  F1NBA0_CHICK  3635/8744/12818/  18738 | 97.2  97.2  96 | 1.1e-172  1.4e-172  0e0 | 4 | 31  (all) | <0.01 | Q |
| **H0YZ67**  **gi\|449477876** | Uncharacterized protein (Fragment)/ Similar to α-1-acid glycoprotein | A7UEB0_CHICK  G1MWU3_MELGA  15394 | 57.1  55.9  47 | 4.1e-39  1.4e-38  2e-33 | 2 | 92  (all) | 0.09 | C,T,Q |
| **gi\|449477880** | Similar to α-1-acid glycoprotein | Q8JIG5_CHICK  G1MWU3_MELGA  15394 | 27.7  26.7  63 | 1.4e-10  2.3e-10  2e-12 | 2 | 74  (all) | 0.09 |  |
| **H0Z046**  **gi\|449478118** | Dynamin-1 (DNM1); shares 2 peptides with H0ZYE3 (DNM2) | E1BXY4_CHICK  G1MY24_MELGA  21148 | 95.3  95.2  99 | 1.2e-153  1.4e-153  9e-72 | 1 | 4  (Z3b) | <0.01 |  |
| **H0Z570**  **gi\|449478260** | Similar to A disintegrin and metalloproteinase with thrombospondin motifs 13 (ADAMTS13-1) | E1C7E8_CHICK  G1N475_MELGA  9241/5277 | 70.2  70.7  82 | 0e0  0e0  1e-114 | 7 | 26  (Z1,3b) | <0.01 | T |
| **H0Z9Z7**  **gi\|449478489** | Similar to olfactomedin-like protein 2A (OLFML2A) | G1N8B3_MELGA  F1P0X8_CHICK  5218 | 89.1  67.8  68 | 6.6e-40  1.1e-39  1e-101 | 3 | 23  (all) | 0.01 | C,T,Q |
| **H0YYC0**  **gi\|449478810** | Similar to soluble calcium-activated nucleotidase 1 (CANT1) | F1NLP1_CHICK  G1N2S5_MELGA  8125/13912 | 88.0  85.4  88 | 1.3e-105  8.4e-49  1e-140 | 5 | 52  (all) | 0.02 | C,T,Q |
| **gi\|449478841**  **H0YZ62** | Similar to protein disulfide isomerase (P4HB) | PDIA1_CHICK  G1N1E5_MELGA  5571/23699 | 84.5  88.4  85 | 2.5e-165  1.2e-134  0e0 | 14 | 107  (all) | 0.03 | C,T,Q |
| **gi\|449479154**  **H0ZBU9** | Similar to α-N-acetylgalactosaminide α-2,6-sialyltransferase 2 (ST6GALNAC2) | SIA7B_CHICK  G1MYB0_MELGA  30526 | 77.3  76.9  92 | 1.8e-124  5.6e-123  2e-24 | 9 | 84  (all) | 0.03 | T |
| **gi\|449479481**  **H0ZH07** | Similar to retinoid-inducible serine carboxypeptidase (SCPEP1) | Q5F3W4_CHICK  G1N836_MELGA  8543 | 83.8  82.8  76 | 7.7e-160  2.1e-158  1e-150 | 4 | 19  (Z1,3b) | 0.01 | C,T,Q |
| **H0YYF6**  **gi\|449479528** | Similar to myeloperoxidase/eosinophil peroxidase (LPO) | F1P3V5_CHICK  G1MSP4_MELGA | 78.2  76.8 | 0e0  0e0 | 4 | 27  (all) | <0.01 | C |
| **gi\|449479736**  **H0Z4W0** | 14-3-3 protein epsilon (YWHAE); shares 3 peptides with other 14-3-3 proteins | 1433E_CHICK  13891 | 99.6  100 | 1.0e-105  1e-120 | 8 | 106  (all) | 0.06 | C,T,Q |
| **H0Z5S2**  **gi\|449479753** | Similar to pigment epithelium-derived factor (PEDF) | G1N131_MELGA  E1C7H6_CHICK  5860 | 90.3  89.6  87 | 6.2e-147  2.7e-146  0e0 | 27 | 1199  (all) | 1.62 | C,T,Q |
| **H0Z4F3**  **gi\|449480101** | Similar to β-glucuronidase (GUSB) | F1NI86_CHICK  G1MZ51_MELGA  10377/12466 | 88.6  85.0  84 | 0e0  0e0  1e-143 | 3 | 27  (all) | 0.01 | T |
| **gi\|449480130**  **H0Z5Q3** | **aa1990-2391** of gi\|449480130 contained all identified peptides and is similar to alpha-2-antiplasmin | G1N0Z7_MELGA  F1NAR5_CHICK  5112/6916 | 77.2  78.2  75 | 5.8e-131  6.0e-128  0e0 | 19 | 539  (all) | 0.11 | C,T,Q |
| **H0Z864**  **gi\|449480248** | Similar to carboxypeptidase D (CPD) | E1BYS4_CHICK  G1N356_MELGA  1909/1604/12177/  21372/19352 | 88.7  89.5  82 | 0e0  0e0  0e0 | 15 | 146  (all) | 0.02 | C,T,Q |
| **H0ZCC0**  **gi\|449480381** | Similar to carbonic anhydrase 4(CA4) | E1C004_CHICK  G1N6Y5_MELGA  17901 | 64.3  64.1  59 | 2.8e-88  1.3e-87  1e-53 | 16 | 387  (all) | 0.32 | C,T,Q |
| **H0YUM7**  **gi\|449480565** | Rab GDP dissociation inhibitor beta (GDI2) | G1MWF4_MELGA  F1NCZ2_CHICK  10820/13389 | 99.5  99.3  85 | 4.7e-192  1.7e-191  1e-149 | 15 | 126  (all) | 0.03 | C,T,Q |
| **gi\|449480691**  **H0YV31** | Similar to inter-α-trypsin inhibitor heavy chain H5 (ITIH5) | G1MYQ9_MELGA  E1BTM0_CHICK  1657/4833/15012/  23213 | 89.7  89.4  72 | 0e0  0e0  0e0 | 34 | 498  (all) | 0.10 | C |
| **H0YWS0**  **gi\|449490222** | Guanine nucleotide-binding protein G(I) subunit α-1 (GNAI1) | F1BXS2_CHICK  G1N2G8_MELGA | 98.2  98.4 | 3.6e-146  4.2e-137 | 3 | 30  (all) | 0.02 | C |
| **H0YXD6**  **gi\|449480905** | Reelin (RELN) | F1NE63_CHICK  G1N471_MELGA  1431/4855/15763/  15998/19442/25109 | 97.0  97.0  76 | 0e0  0e0  0e0 | 6 | 11  (Z1,3b) | <0.01 |  |
| **gi\|449480925**  **H0YXT6** | **aa108-938** similar to cadherin-related family member 3 | E1BZR7_CHICK  G1N4Q7_CHICK  17097 | 70.6  71.7  59 | 3.4e-71  1.5e-70  1e-59 | 12 | 265  (all) | 0.04 | C,T |
| **H0YYP9**  **gi\|449480953** | Similar to laminin subunit beta-1 (LAMB1) | G1NC71_MELGA  F1NJ23_CHICK  6802/10263 | 88.7  92.8  90 | 0e0  0e0  0e0 | 4 | 8  (Z1,3b) | <0.01 | C,T,Q |
| **gi\|449481035**  **H0Z2H6** | Protein FAM3C | E1BWA9_CHICK  G1N943_MELGA | 95.8  95.3 | 1.9e-79  7.3e-79 | 2 | 3  (Z1,3b) | <0.01 | C,T |
| **H0Z1M8**  **gi\|449481277** | Similar to V-type proton ATPase subunit S1 (ATP6AP1) | G1N8K5_MELGA  F1NNV6_CHICK  29511 | 80.3  78.9  91 | 8.2e-147  4.4e-142  1e-32 | 7 | 116  (all) | 0.10 | C,T |
| **H0Z9B1**  **gi\|449481456** | Similar to cathepsin E-A | G1NPE0_MELGA  E1C897_CHICK  22801 | 89.5  88.4  92 | 4.9e-144  1.2e-142  2e-51 | 8 | 55  (all) | 0.05 | T |
| **H0Z834**  **gi\|449481578** | Similar to N-acetylglucosamine-6-sulfatase (GNS) | F1NI04_CHICK  G1ND08_MELGA  12604/14217 | 93.9  95.5  94 | 0e0  9.3e-216  1e-124 | 13 | 397  (all) | 0.36 | C,T,Q |
| **H0ZJ37**  **gi\|449481963** | Similar to chromobox protein 6/Neuronal pentraxin receptor (NPTXR) | E1BUW1_CHICK  G1NL88_MELGA  10920/11231 | 93.5  93.1  94 | 2.8e-209  8.0e-191  1e-144 | 3 | 20  (Z3,3b) | <0.01 | Q |
| **H0ZKL6**  **gi\|449481995** | Myosin-9 (MYH9) | MYH9_CHICK  G1NJB2_MELGA  164 | 95.8  95.8  95 | 0e0  0e0  0e0 | 14 | 34  (Z1,3b) | <0.01 | T |
| **H0ZPG9**  **gi\|449482118** | Similar to poly(U)-specific endoribonuclease-A | E1C036_CHICK  G1NLW9_MELGA | 82.8  82.1 | 5.6e-108  7.9e-106 | 7 | 85  (all) | 0.06 |  |
| **gi\|449482150**  **H0ZP53** | Similar to signal peptide, CUB and EGF-like domain-containing protein 1 (SCUBE1) | F1NZ94_CHICK  G1NLZ2_MELGA  12923/16797 | 94.5  96.9  96 | 0e0  0e0  2e-98 | 14 | 210  (all) | 0.04 | C,T,Q |
| **H0YQR9**  **gi\|449482627** | Uncharacterized protein (Fragment; TPP1)/similar to Tripeptidyl peptidase I | F1NB83_CHICK  H9H1Z2_MELGA  17464 | 73.8  73.7  76 | 7.9e-89  5.7e-83  8e-71 | 7 | 66  (all) | 0.03 | C,T,Q |
| **gi\|449482631** | Protocadherin-16; shares 16 peptides with H0YQS4 | E1C566_CHICK  G3US48_MELGA  1797/2995/3699/  4974 | 82.1  68.1  76 | 0e0  0e0  0e0 | 20 | 207  (all) | 0.01* | T |
| **H0YQS4** | Uncharacterized protein (DCHS1)/similar to dachsous cadherin-related 1; shares 16 peptides with gi\|449482631 | E1C566_CHICK  G3US48_MELGA  1797/2995/3699/  4974 | 89.3  74.7  81 | 0e0  0e0  0e0 | 1 | 10  (Z1,3b) |  |  |
| **H1A104**  **gi\|449482682** | Similar to transmembrane protease serine 2 (TMPRSS2) | F1NY88_CHICK  G1NP04_MELGA  6804 | 79.3  77.2  65 | 7.3e-110  8.4e-109  8e-69 | 3 | 15  (Z1,3,3b) | <0.01 | C,T |
| **H0Z8C1**  **gi\|449482762** | Renin/prorenin receptor (ATP6AP2) | F1NB42_CHICK  G1NP47_MELGA  20902/22830 | 89.8  89.2  71 | 1.8e-128  7.3e-128  3e-61 | 10 | 200  (all) | 0.19 | C,T,Q |
| **H0ZAF6**  **gi\|449482803** | Peroxiredoxin-4 (PRDX4); shares 1 peptide with B5G0M2 (PRDX1) | G1NP83_MELGA  F1NNS8_CHICK | 99.5  98.4 | 1.3e-86  1.3e-85 | 6 | 53  (all) | 0.04 | C,T |
| **H0ZLP1**  **gi\|449483778** | DnaJ homolog subfamily C member 3 (DNAJC3) | F1P053_CHICK  G1NPW6_MELGA  5586 | 96.1  95.8  86 | 5.5e-146  3.6e-143  0e0 | 14 | 136  (all) | 0.03 | C,T,Q |
| **gi\|449484555**  **H0ZQD0** | Similar to olfactomedin-4 (OLFM4) | G1NQ14_MELGA  F1NXH9_CHICK  10423/10854 | 70.1  69.9  74 | 8.8e-135  7.0e-134  1e-120 | 12 | 10  (all) | 0.02 | C,T,Q |
| **H1A515**  **gi\|449484659** | Similar to stromelysin-1/matrilysin (MMP7) | G3UV36_MELGA  Q5ZMQ8_CHICK  19792 | 71.2  72.1  80 | 9.4e-84  1.8e-83  3e-68 | 8 | 62  (all) | 0.02 |  |
| **H0ZRR0**  **gi\|449484747** | Similar to serine protease 23 (PRSS23) | E1C744_CHICK  G1NRE7_MELGA  7257/8220 | 96.2  96.2  96 | 4.7e-163  1.2e-162  0e0 | 3 | 17  (Z2,3b) | <0.01 | C,T,Q |
| **H0ZS52**  **gi\|449484831** | Similar to Tsukushin (TSKU) | F1NDH7_CHICK  G1NRF2_MELGA  7859 | 89.6  86.7  88 | 3.2e-115  2.0e-108  1e-172 | 6 | 120  (all) | 0.06 | C,T,Q |
| **gi\|449484853**  **H0ZSC5** | Similar to solute carrier family 2, facilitated glucose transporter member 3 (SLC2A3) | G1NME1_MELGA  F1NTJ1_CHICK  9238/9954 | 88.7  87.0  66 | 7.0e-161  2.6e-157  1e-129 | 10 | 137  (all) | 0.11 | C,Q |
| **H0ZRP9**  **gi\|449485032** | Similar to dipeptidyl-peptidase 1 (CTSC) | F1NWG2_CHICK  G1NQP8_MELGA  14839 | 90.4  88.9  79 | 4.3e-180  7.5e-178  1e-113 | 6 | 113  (all) | 0.05 | C,T,Q |
| **gi\|449485099**  **H1A5U5** | Similar to ovostatin | F1NEW8_CHICK  G1NK51_MELGA  455/25086 | 75.1  74.6  75 | 0e0  0e0  1e-165 | 64 | 1474  (all) | 0.23 | C,T,Q |
| **H0ZS92**  **gi\|449485103** | Similar to α-2-macroglobulin (A2M-3); shares 13 peptides with H0ZS79 (A2M-2) | E1C544_CHICK  G1NMF6_MELGA  4167/10397/13509/  25258 | 77.8  72.2  68 | 0e0  0e0  1e-163 | 5 | 11  (Z1,3,3b) | <0.01 | C,Q |
| **gi\|449485115** | Similar to α-2-macroglobulin (A2M-1); shares 20 peptides with H0ZSA5 and 3 with H0ZSA0 | E1C544_CHICK  G1NMI3_MELGA  6814/16447/18513 | 35.0  33.8  85 | 1.4e-163  1.4e-152  2e-93 | 35 | 554  (all) | 0.04* | (C) |
| **H0ZSA5** | Similar to α-2-macroglobulin-like 1 (A2ML1)/ovostastin-like; shares 20 peptides with gi\|449485115 | G1NME9_MELGA  F1NTK2_CHICK  30/6814/16447 | 51.7  69.6  67 | 1.8e-184  1.8e-96  0e0 | 4 | 31  (all) |  |  |
| **H0ZSQ9**  **gi\|449485223** | Similar to complement C1r subcomponent (C1RL) | G1NMY0_MELGA  F1NAB7_CHICK  2875/5917/12223 | 81.5  80.4  57 | 0e0  1.0e-187  0e0 | 6 | 31  (Z1,3b) | <0.01 | T |
| **gi\|449485361**  **H0ZT52** | Limbic system-associated membrane protein (LSAMP) | G3UV27_MELGA  LSAMP_CHICK  24578/26356 | 99.3  98.6  100 | 2.0e-124  1.0e-123  2e-46 | 9 | 51  (all) | 0.07 |  |
| **gi\|449485458**  **H0ZT25** | Similar to follistatin-related protein 1 (FSTL1) | F1NM70_CHICK  G1NN97_MELGA  10961 | 67.1  61.9  80 | 1.8e-60  2.9e-45  1e-113 | 6 | 21  (Z3,3b) | 0.01 | C,Q |
| **H0ZTE6**  **gi\|449485775** | Similar to vitamin K-dependent protein S (PROS1) | G1NNL9_MELGA  E1C6L4_CHICK | 89.2  87.9 | 0e0  3.2e-214 | 12 | 255  (all) | 0.12 | C,T |
| **H0ZTG0**  **gi\|449485798** | Similar to cellular repressor of E1A-stimulated genes 1 (CREG1) | CREG1_CHICK  G1NNN9_MELGA  28044 | 87.2  93.2  91 | 4.9e-77  2.6e-48  4e-30 | 3 | 39  (all) | 0.05 | C,T,Q |
| **gi\|449485917**  **H0ZTL2** | Similar to amyloid β A4 protein (APP); shares 1 peptide with H0YQL6 (APLP2) | F1C440_CHICK  G1NNT9_MELGA  2256/21843 | 95.8  95.6  87 | 3.8e-120  6.7e-120  0e0 | 5 | 36  (Z1,3b) | <0.01 | C,T,Q |
| **gi\|449486029**  **H0ZVL5** | Similar to leukocyte surface antigen CD47 | Q6XFR0_CHICK  G1NNJ4_MELGA | 47.6  54.8 | 1.2e-39  3.2e-15 | 3 | 17  (Z1,3,3b) | <0.01 |  |
| **gi\|449486185**  **H0Z3M3** | Syndecan-4 (SDC4) | Q5I5K4_MELGA  SDC4_CHICK  16511/19784 | 88.1  86.9  87 | 2.6e-62  1.3e-60  2e-81 | 2 | 21  (Z1,2,3b) | 0.02 | C,T |
| **gi\|449486338**  **H0Z417** | Similar to 14-3-3 protein β/α (YWHAB); shares 3 peptides with other 14-3-3 proteins | G1MXY2_MELGA  1433B_CHICK  12358/20781 | 85.7  85.7  78 | 1.7e-77  3.7e-77  4e-57 | 5 | 40  (Z1,3b) | 0.01 | T,Q |
| **H0Z487**  **gi\|449486344** | Similar to adenosine deaminase (ADA) | G1MXM4_MELGA  F1P2A6_CHICK  21214/10723 | 82.8  82.2  81 | 4.4e-131  7.2e-131  7e-64 | 10 | 112  (all) | 0.04 |  |
| **H0Z6V7**  **gi\|449486379** | Similar to carboxypeptidase (CTSA) | F1NIN2_CHICK  G1N8A7_MELGA  7861/12705 | 90.7  88.3  84 | 2.1e-194  2.2e-188  0e0 | 11 | 229  (all) | 0.15 | C,T,Q |
| **H0ZCQ0**  **gi\|449486463** | Guanine nucleotide-binding protein G(S) subunit α (GNAS) | F1NM72_CHICK  G1N9J0_MELGA  27012 | 94.4  98.7  91 | 3.2e-140  2.9e-136  2e-39 | 5 | 30  (all) | 0.01 | C,T |
| **H0ZE54**  **gi\|449486501** | Phospholipid-translocating ATPase (ATP9A) | F1NUR8_CHICK  G1NAQ5_MELGA  6049/9653/25043 | 98.5  96.4  73 | 0e0  0e0  1e-128 | 2 | 11  (Z1,Z3b) | <0.01 |  |
| **H0YXS3**  **gi\|449486900** | Guanine nucleotide binding protein (G protein), β-polypeptide 2 (GNB1) | F1NLV4_CHICK  G1NBT6_MELGA  18276 | 98.9  90.9  91 | 1.1e-146  1.0e-115  1e-88 | 5 | 45  (all) | 0.02 | C,T |
| **gi\|449486956**  **H0YY23** | Similar to von Willebrand factor A domain-containing protein 1 (VWA1) | F1NPH3_CHICK  G1MUX6_MELGA  1692/9912 | 81.2  75.5  74 | 1.0e-122  4.7e-107  0e0 | 12 | 177  (all) | 0.07 | C,T,Q |
| **H0YZL6**  **gi\|449487042** | Similar to calsyntenin-1 (CLSTN1) | E1BYQ5_CHICK  G1MXV5_MELGA  1237 | 94.9  93.2  93 | 0e0  0e0  0e0 | 17 | 187  (all) | 0.05 | T,Q |
| **gi\|449487095**  **H0Z0S3** | Similar to agrin (AGRN) | G3UQJ3_MELGA  F1NWQ3_CHICK  166/1170/18626/  23251 | 63.9  64.9  60 | 1.9e-212  2.2e-175  0e0 | 18 | 166  (all) | 0.01 | C,T,Q |
| **H0Z0E4**  **gi\|449488113** | Similar to glutamine-fructose-6-phosphate aminotransferase [isomerizing] 1 (GFPT1) | Q5ZIG5_CHICK  G1MPR9_MELGA  2646/17387 | 86.4  86.3  86 | 1.5e-188  3.8e-188  0e0 | 5 | 33  (Z1,3b) | <0.01 |  |
| **gi\|449488183**  **H0Z269** | Similar to ADAM28 (ADAM7) | G1MR45_MELGA  F1NU67_CHICK  3606 | 76.2  76.2  56 | 1.2e-110  9.3e-110  1e-161 | 9 | 107  (all) | 0.03 | C,T,Q |
| **H0Z285**  **gi\|449488187** | Similar to stanniocalcin-1(STC1) | F1NU46_CHICK  G1MR66_MELGA  6907 | 89.9  90.0  90 | 1.4e-98  3.7e-85  1e-119 | 9 | 309  (all) | 0.27 | C,T,Q |
| **H1A3U9**  **gi\|449488345** | Similar to lysyl oxidase 2 (LOXL2) | G1MRH0_MELGA  LOXL2_CHICK  10096/13117/12356/  20794 | 91.1  90.9  74 | 7.6e-203  7.8e-202  1e-128 | 18 | 477  (all) | 0.30 | C,T,Q |
| **H0Z117**  **gi\|449488711** | Similar to keratin, type II cytoskeletal cochleal (KRT78); shares 2 peptides with CON_P13647, 6 with CON_P02538 and 1 with CON_P04264 | G1NF62_MELGA  K2CO_CHICK  15221/14618 | 90.3  89.3  84 | 4.9e-80  3.1e-79  2e-61 | 8 | 46  (all) | 0.01 | C,T,Q |
| **H0YS66**  **gi\|449488959** | α-1,2-(MAN1C1); shares 1 peptide with H0ZNP7 (MAN1A1) | F1P0Z9_CHICK  G1MSL3_MELGA  8085/3839 | 89.1  88.8  88 | 4.2e-182  6.1e-182  1e-149 | 2 | 5  (Z3b) | <0.01 |  |
| **gi\|449489074**  **H0YTP8** | β-xylosidase/α-L-arabinofuranosidase 2-like; domains: Glycoside hydrolase family 3, FN3_like | H9KYZ6_CHICK  8019 | 79.5  85 | 2.0e-22  2e-71 | 11 | 64  (all) | 0.01 | Q |
| **gi\|449489078** | Collagen VIII, α-2 (COL8A2); shares 1 peptide with H0ZNY7 (COL10A1) | F1P194_CHICK | 89.7 | 1.5e-98 | 4 | 21  (Z1,3,3b) | 0.01 |  |
| **H0YQC7**  **gi\|449489234** | Similar to sortilin-related receptor (SORL1) | G3UQY4_MELGA  E1BUD4_CHICK  3860/14813 | 92.3  92.7  81 | 0e0  0e0  0e0 | 7 | 31  (Z1,3b) | <0.01 | C,T,Q |
| **H0YP50**  **gi\|449489268** | Uncharacterized protein (HYOU1) | G3UTS6_MELGA  HYOU1_CHICK  1123/1302 | 87.0  86.6  86 | 6.3e-206  1.7e-205  0e0 | 17 | 122  (all) | 0.01 | C,T,Q |
| **H0YQ36**  **gi\|449489436** | Uncharacterized protein (MCAM/S-gicerin) | Q98923_CHICK  G3US47_MELGA  12290 | 65.1  65.1  64 | 2.2e-81  1.9e-81  4e-54 | 3 | 18  (Z1,3b) | 0.01 |  |
| **gi\|449490475**  **H0YTI4** | Complement component receptor 1-like | G1MYD1_MELGA  R4GHS1_CHICK  2279 | 42.2  51.9  38 | 1.5e-193  1.3e-113  5e-49 | 3 | 6  (Z2,3b) | <0.01 |  |
| **H1A4C0**  **gi\|449490637** | Similar to acidic mammalian chitinase (CHIA) | G3UUJ2_MELGA  F1NM31_CHICK  12721/16224 | 71.1  70.3  69 | 6.7e-47  7.7e-47  1e-101 | 2 | 20  (all) | 0.02 |  |
| **gi\|449491026**  **H0YW18** | Plakoglobin (JUP) | E1C1V3_CHICK  G1NCE5_MELGA  18134 | 97.8  96.8  98 | 0e0  9.2e-133  5e-74 | 17 | 79  (all) | 0.01 | C,T,Q |
| **H0YW77**  **gi\|449491211** | Similar to 2',3'-cyclic-nucleotide 3'-phosphodiesterase (CNP) | G1MW69_MELGA  O57389_CHICK  6550/12516 | 88,6  86.2  85 | 1.6e-152  4.0e-150  0e0 | 4 | 17  (Z1,3b) | <0.01 |  |
| **gi\|449491300**  **H0YXB2** | Similar to synaptic vesicle membrane protein VAT-1-like protein | F1NBS9_CHICK  G1MYR9_MELGA  7957/7835 | 89.4  88.2  87 | 2.9e-95  3.0e-94  1e-119 | 8 | 104  (all) | 0.04 |  |
| **gi\|449491688**  **H0YQK2** | Guanine nucleotide-binding protein subunit α-11 (GNA11); shares 1 peptide with H0YYZ8 (GNAQ) | H9KZ66_CHICK  GNA11_MELGA  11559 | 100.0  99.7  76 | 1.6e-132  1.6e-132  1e-121 | 5 | 32  (all) | 0.01 | C,Q |
| **H0YPY3**  **gi\|449491779** | Uncharacterized protein (MUC16) | F1NIZ7_CHICK  G1MSW6_MELGA  16426 | 41.8  38.8  65 | 1.0e-44  8.7e-24  9e-63 | 2 | 11  (all) | <0.01 | T,Q |
| **gi\|449491826**  **H0YQC3** | Similar to C3 and PZP-like α-2-macroglobulin domain-containing protein 8 (CPAMD8) | G1N1H6_MELGA  F1NN85_CHICK  2615/5166/1099 | 93.1  93.6  85 | 0e0  0e0  0e0 | 14 | 60  (Z1,3b) | <0.01 | C,T,Q |
| **H0YR81**  **gi\|449491890** | Similar to receptor-type tyrosine-protein phosphatase S (PTPRS); shares 1 peptide with H0ZAM5 (PTPRF) | G3UQ69_MELGA  F1NWE3_CHICK  577/8051 | 94.2  75.0  89 | 0e0  0e0  0e0 | 5 | 15  (Z1,2,3b) | <0.01 | C,Q |
| **gi\|449491943**  **H0YRX1** | Elongation factor 2 (EEF2) | G1NFA2_MELGA  F1NFS0_CHICK  1643/3005 | 98.9  98.9  98 | 4.1e-173  4.5e-173  0e0 | 14 | 99  (all) | 0.02 | C,T,Q |
| **gi\|449492047**  **H0YQ44** | Uncharacterized protein (VILL)/villin-1 | E1C5U6_CHICK  G1MWE6_MELGA  1309 | 87.8  86.6  60 | 0e0  0e0  0e0 | 19 | 205  (all) | 0.03 | C,T,Q |
| **H0YST2**  **gi\|449492211** | Similar to vimentin (VIM) | F1NJ08_CHICK  G1N6W4_MELGA  8582/9534/25615 | 97.1  98.8  96 | 5.6e-111  4.3e-60  1e-174 | 5 | 12  (Z1,3b) | <0.01 | C |
| **H0YVD4**  **gi\|449492350** | TUBB2A-1/similar to tubulin β-chain (1/2/3/7); shares 10 peptides with B5G4G7 | G1K338_CHICK  G3X8P3_MELGA  5781 | 99.8  99.8  98 | 2.2e-197  5.2e-196  0e0 | 2 | 12  (all) | <0.01 | C,T,Q |
| **gi\|449492508**  **H0YVV0** | Similar to desmoplakin (DSP) | E1BWI0_CHICK  G1MXH6_MELGA  234/108 | 91.6  90.4  77 | 0e0  0e0  0e0 | 20 | 111  (all) | 0.01 | C,T,Q |
| **gi\|449492705**  **H0YXX4** | Similar to biotinidase isoform 2 (BTD);shares 7 peptides with H1A3L5 (Similar to Biotinidase) | E1C3J7_CHICK  G1NED4_MELGA  6873 | 82.7  82.4  86 | 1.3e-120  5.0e-120  0e0 | 10 | 179  (all) | 0.07 | C,T,Q |
| **gi\|449492973**  **H0Z538** | Similar to acyloxyacyl hydrolase (AOAH) | F1NF94_CHICK  G1NHE6_MELGA  20373/20856 | 84.1  79.8  87 | 1.8e-215  1.8e-202  4e-52 | 4 | 11  (Z1,3b) | <0.01 |  |
| **gi\|449493197** | Similar to prostatic acid phosphatase-like; domain: Histidine phosphatase superfamily | F1NT31_CHICK  G1NG25_MELGA | 52.9  51.5 | 1.9e-85  8.9e-82 | 16 | 346  (all) | 0.29 | (C) |
| **H0Z2A9**  **gi\|449493221** | β-galactosidase (GLB1); | G1NGW0_MELGA  Q5ZLM4_CHICK  9639/12865/19780 | 85.5  90.0  72 | 0e0  3.7e-137  1e-119 | 9 | 205  (all) | 0.09 | C,T,Q |
| **H0ZEH2**  **gi\|449493878** | Programmed cell death protein 6 (PDCD6) | F1NHD8_CHICK  G1N6J5_MELGA  27206 | 95.4  96.2  96 | 6.2e-70  2.7e-63  5e-48 | 2 | 5  (Z2,3,3b) | <0.01 | C,T,Q |
| **gi\|449493914**  **H0ZDW4** | Similar to polypeptide N-acetylgalactosaminyltransferase (GALNT12) | G1N5V3_MELGA  F1NMD3_CHICK  3797 | 92.6  92.0  61 | 3.3e-175  6.9e-105  0e0 | 2 | 14  (all) | <0.01 | C,T,Q |
| **gi\|449494054**  **H0ZKE0** | Similar to transthyretin | G1NDK1_MELGA  TTHY_CHICK | 70.7  65.3 | 3.7e-43  1.1e-40 | 5 | 20  (Z1,2,3b) | 0.05 | C |
| **gi\|449494147**  **H0ZLI3** | Inositol monophosphatase 3 (IMPAD1) | E1C648_CHICK  G1NEV7_MELGA  12646/25462 | 92.6  96.5  97 | 7.0e-117  3.3e-93  5e-46 | 5 | 75  (all) | 0.05 | C,T,Q |
| **H0ZHK2**  **gi\|449494664** | Protein APCDD1 | APCD1_CHICK  G1N9Y6_MELGA  7974/19789 | 95.9  93.9  90 | 0e0  0e0  0e0 | 12 | 105  (all) | 0.03 | C,T,Q |
| **gi\|449494704**  **H0ZII9** | Similar to emilin-2 | G1NBE1_MELGA  F1NBG2_CHICK  1455 | 76.5  80.3  82 | 3.2e-140  5.7e-101  0e0 | 6 | 17  (Z1,3b) | <0.01 | Q |
| **H0ZLW2**  **gi\|449494846** | Similar to γ-glutamyl hydrolase (GGH) | F1NR48_CHICK  G1NF82_MELGA  26558 | 79.4  85.7  73 | 3.7e-117  2.2e-94  3e-22 | 8 | 163  (all) | 0.08 | C,T,Q |
| **H0ZNQ7**  **gi\|449494986** | Similar to cadherin-17 (CDH17) | E1BX79_CHICK  G1NHF7_MELGA  4539/24961 | 69.1  69.1  74 | 0e0  0e0  1e-172 | 4 | 16  (Z1,2,3b) | <0.01 |  |
| **H0ZYW1**  **gi\|449512482** | Serine/threonine-protein phosphatase (PPP1CB) | G1NDD0_MELGA  PP1B_CHICK  12530 | 99.5  98.9  98 | 2.9e-90  1.2e-89  2e-93 | 2 | 3  (Z1,3b) | <0.01 | T |
| **H0Z2D1**  **gi\|449495976** | EH domain-containing protein 3 (EHD3); shares 4 peptides with H0ZL62 (EHD4) | F1NGM0_CHICK  G1N1U5_MELGA  14498/19626/28794/  29019 | 97.8  99.6  93 | 1.2e-204  3.2e-179  1e-104 | 22 | 196  (all) | 0.04 | T,Q |
| **gi\|449496019**  **H0Z503** | Similar to epithelial cell adhesion molecule (EPCAM) | G1N0W4_MELGA  EPCAM_CHICK  24218 | 76.6  74.6  69 | 5.3e-92  5.6e-91  3e-33 | 2 | 7  (Z1,3b) | <0.01 | C,T |
| **gi\|449496679** | HSP90-β; shares 5 peptides with H0ZRE5 (HSP 90-α) | G1NEA9_MELGA  F1NC33_CHICK  C7G498_COTJA | 64.2  64.3  64.2 | 3.8e-110  5.3e-110  1.4e-109 | 2 | 31  (all) | 0.01 | C,T,Q |
| **H0ZGX0**  **gi\|449496874** | Similar to ribonuclease T2 (RNASET2) | Q7LZG0_CHICK  G1NI79_MELGA | 76.8  76.4 | 4.1e-75  4.8e-74 | 5 | 47  (Z1,3,3b) | 0.02 | T |
| **gi\|449497133**  **H0ZLH1** | Sorting nexin (SNX9) | E1BTY4_CHICK  G1NJK8_MELGA  17675/22619 | 95.5  88.6  97 | 0e0  8.6e-214  6e-93 | 2 | 5  (Z3,3b) | <0.01 |  |
| **H0ZJE2**  **gi\|449497279** | Similar to ERO1-like β (ERO1LB); shares 1 peptide with H0ZSA4 (ERO1L) | E1C917_CHICK  G1NGY0_MELGA | 93.1  93.0 | 2.0e-190  8.8e-183 | 12 | 203  (all) | 0.07 | T |
| **H0ZLB9**  **gi\|449497375** | Ezrin (EZR); shares 10 peptides with H0YX94 (moesin) | G1NJR0_MELGA  Q9YGW6_CHICK  4836/4547 | 96.2  96.2  85 | 1.4e-126  1.4e-126  0e0 | 30 | 410  (all) | 0.14 | C,T,Q |
| **gi\|449497556**  **H0ZMA1** | Similar to LOC100227597/**aa65-1720** fibronectin type III domain-containing protein 1 (FNDC1) | G1NIR6_MELGA  F1NSG1_CHICK  18760/14151/18304/  19121/20983 | 68.5  69.3  93 | 2.8e-128  4.8e-124  6e-94 | 85 | 2342  (all) | 0.39 | C |
| **H0ZNP7**  **gi\|449497887** | α-1,2-mannosidase (MAN1A1); shares 1 peptide with H0YS66 (MAN1C1) | F1N9D0_CHICK  G1NKV0_MELGA  11923/24808 | 94.4  95.0  97 | 6.6e-199  3.2e-191  1e-116 | 14 | 166  (all) | 0.10 | C,T |
| **H0ZNZ2**  **gi\|449497901** | Similar to non-specific protein-tyrosine kinase (FRK) | E1C2F6_CHICK  G1NKZ9_MELGA  16015/20433 | 83.2  83.4  78 | 7.1e-101  6.6e-97  4e-79 | 3 | 17  (Z1,3b) | <0.01 |  |
| **H0ZQZ8**  **gi\|449498102** | Similar to CD109 | F1NX21_CHICK  G1NM32_MELGA  5136/22279 | 81.6  80.1  85 | 0e0  0e0  2e-82 | 11 | 33  (Z2,3,3b) | <0.01 | T |
| **H0ZS56**  **gi\|449498157** | Protein disulfide-isomerase A6 (PDIA6) | G1NML0_MELGA  F1NK96_CHICK  22852 | 96.1  95.1  95 | 2.4e-183  2.9e-182  5e-65 | 2 | 15  (Z1,3b) | <0.01 | C,T |
| **H0ZSG7**  **gi\|449498224** | Similar to apolipoprotein B-100 (APOB) | F1NV02_CHICK  G1NMN7_MELGA  8/277/129/1095 | 79.1  76.8  79 | 0e0  0e0  0e0 | 38 | 195  (all) | <0.01 | C |
| **H0YVQ8**  **gi\|449498416** | Similar to tetraspanin-6 (TSPAN6) | E1C857_CHICK  G1MYM6_MELGA  14051/18417 | 86.2  85.0  74 | 6.7e-64  3.0e-62  6e-48 | 2 | 17  (Z1,3b) | 0.01 | C,T,Q |
| **H0YX94**  **gi\|449498505** | Moesin; shares 10 peptides with H0ZLB9 (Ezrin) | E1BV34_CHICK  G1MQ39_MELGA  3203 | 98.3  98.3  97 | 1.1e98  3.0e-94  0e0 | 2 | 2  (Z3b) | <0.01 | C |
| **H0YXR4**  **gi\|449498865** | Glypican-4 (GPC4) | F1NAU1_CHICK  G1MVZ0_MELGA  6640/16694/14949 | 94.7  94.3  84 | 6.2e-210  1.3e-209  0e0 | 9 | 73  (all) | 0.02 | C,T,Q |
| **gi\|449499399**  **H0YTL4** | Similar to serum albumin (ALB) | G1NCR2_MELGA  ALBU_CHICK  16345/19800 | 71.5  69.6  80 | 6.6e-213  7.2e-207  1e-87 | 56 | 2857  (Z1,2,3, 3b) | 2.93 | C,T,Q |
| **gi\|449499828**  **H0YVF8** | Uncharacterized (Fragment; ABCG2)/similar to ATP-binding cassette sub-family G member 2 | E1C5B1_CHICK  G1N6J2_MELGA  2993 | 64.0  65.6  55 | 7.4e-51  3.8e-76  0e0 | 3 | 5  (Z1,3b) | <0.01 |  |
| **H0YZQ4**  **gi\|449500255** | Similar to β-mannosidase (MANBA) | F1NBF1_CHICK  G1NFQ1_MELGA  8456 | 85.3  84.6  62 | 0e0  0e0  1e-120 | 7 | 48  (all) | 0.01 | C,T |
| **H0Z095**  **gi\|449500286** | Similar to nephronectin (NPNT) | G1N2S7_MELGA  E1C8K1_CHICK  19411 | 83.3  78.3  88 | 3.2e-151  2.3e-65  2e-31 | 17 | 260  (all) | 0.11 | C,T,Q |
| **H0Z6B3**  **gi\|449500451** | Similar to carboxypeptidase E (CPE) | G1MTM8_MELGA  R4GFJ0_CHICK  6735/24538 | 95.2  93.8  95 | 2.2e-164  9.5e-131  0e0 | 14 | 146  (all) | 0.05 | C,Q |
| **H0Z7V0**  **gi\|449500550** | Similar to N(4)-(β-N-acetylglucosaminyl)-L-asparaginase/aspartylglucosaminidase (AGA) | F1NQI0_CHICK  G1N4J2_MELGA | 87.2  84.2 | 1.8e-124  4.2e-121 | 5 | 79  (all) | 0.04 | C,T |
| **gi\|449500756**  **H0Z9H0** | Similar to protocadherin Fat 1 (FAT1) | G1NG27_MELGA  F1NWW5_CHICK  483/376/2159 | 91.7  92.0  89 | 0e0  0e0  0e0 | 5 | 15  (Z1,3b) | <0.01 | C,T,Q |
| **H0ZHL2**  **gi\|449501028** | Similar to ADP-ribosyl cyclase 2 (BST1) | F1NG83_CHICK  G1NJ40_MELGA | 79.3  78.9 | 3.4e-93  2.2e-93 | 3 | 14  (Z1,3b) | 0.01 | C,T |
| **gi\|449501232**  **H0ZHJ9** | Similar to prominin-1 A (PROM1) | G1NJ24_MELGA  F1CLF0_CHICK  22484/23281 | 84.9  84.1  90 | 0e0  0e0  1e-65 | 7 | 82  (all) | 0.01 | T |
| **H0ZIJ5**  **gi\|449501297** | Similar to epididymis-specific alpha-mannosidase (MAN2B2) | G1NJI0_MELGA  F1NQF6_CHICK  3610/22648/27848 | 83.2  83.9  76 | 0e0  6.4e-159  5e-69 | 34 | 782  (all) | 0.21 | C,T,Q |
| **H0ZEB8**  **gi\|449501999** | Similar to nucleobindin-2 (NUCB2) | F1NGB1_CHICK  G1N538_MELGA  7172/6496 | 94.7  94.3  98 | 1.6e-104  5.7e-104  0e0 | 32 | 1090  (all) | 0.76 | C,T,Q |
| **H0ZH08**  **gi\|449502119** | Similar to mucin-5B | MUC5B_CHICK  G1N988_MELGA  1055/5308/9118 | 88.4  88.0  66 | 0e0  0e0  1e133 | 10 | 89  (all) | 0.01 | C,T,Q |
| **H0ZHH9**  **gi\|449502121** | Similar to chitinase domain-containing protein 1 (CHID1) | F1NER5_CHICK  G1N9S4_MELGA | 93.3  89.8 | 1.7e-159  8.8e-127 | 8 | 78  (all) | 0.03 | C,T |
| **gi\|449502358**  **H0ZL62** | Similar to EH domain-containing protein 4 (EHD4); shares 4 peptides with H0Z2D1 (EHD3) | G1NFT2_MELGA  F1NV47_CHICK  6467/13766/17078/  24018/29768 | 94.2  94.2  78 | 9.6e-176  2.7e-175  1e-175 | 15 | 206  (all) | 0.03 | T,Q |
| **H0Z7H5**  **gi\|449503956** | Similar to transmembrane protein 132A (TMEM132A) | R4GGR7_CHICK  H9H102_MELGA | 78.3  59.4 | 1.8e-85  2.2e-80 | 9 | 67  (all) | 0.02 | C,T |
| **H0Z9E5**  **gi\|449504022** | Similar to plasma protease C1 inhibitor (SERPING1) | F1NA58_CHICK  G1NBE9_MELGA  24662/13582/26678 | 72.9  71.2  70 | 1.4e-88  1.2e-85  2e-34 | 6 | 85  (all) | 0.03 | C,T,Q |
| **gi\|449504198**  **H0ZGZ6** | Mucin-5AC; shares 53 peptides with H0ZGY9 and 18 with H0ZGW8 | E1C037_CHICK  G1N8Z1_MELGA  6295/4106/10751/108067 | 65.4  80.4  83 | 0e0  0e0  0e0 | 88 | 4468  (all) | 0.54* | C,T,Q |
| **H0ZGY9** | Similar to mucin-5AC; shares 53 peptides with gi\|449504198 | G1N8Z1_MELGA  E1C037_CHICK  6295/4106/10751/108067 | 77.5  71.0  83 | 0e0  0e0  0e0 | 2 | 207  (all) |  |  |
| **H0ZGW8** | Uncharacterized protein (MUC5AC); shares 18 peptides with gi\|449504198 | G1N931_MELGA  6950/1527 | 74.3  69 | 0e0  1e-147 | 2 | 48  (all) |  |  |
| **gi\|449504355**  **H0ZME2** | Similar to SPARC-related modular calcium-binding protein 1 (SMOC1) | F1NY60_CHICK  G1NGZ7_MELGA  28143 | 89.1  90.3  100 | 1.6e-128  2.2e-121  4e-44 | 5 | 48  (Z1,3b) | 0.01 |  |
| **H0ZML6**  **gi\|449504363** | α-actinin-1 (ACTN1) | R9PXN4_CHICK  G1NH44_MELGA  1534/1475/21820/  24732 | 99.6  99.3  97 | 0e0  0e0  0e0 | 12 | 27  (all) | <0.01 | C,T,Q |
| **H0ZN18**  **gi\|449504370** | Similar to thrombospondin-1 (THBS1) | F1P0J8_CHICK  G1NHK4_MELGA  724/1249/11945 | 94.6  95.5  94 | 0e0  0e0  0e0 | 12 | 53  (Z1,3b) | <0.01 | T,Q |
| **gi\|449504457**  **H0ZQ74** | Similar to galactocerebrosidase (GALC) | G1NJZ8_MELGA  F1NJ89_CHICK  22350/27739 | 88.2  87.2  88 | 0e0  0e0  4e-64 | 6 | 51  (all) | 0.01 | C,T,Q |
| **gi\|449504486**  **H0ZQY4** | Similar to plasma serine protease inhibitor/ α-1-antiproteinase 2-like (SERPINA12-2/A3) | F1NPN5_CHICK  G1NKH5_MELGA  22439 | 76.0  48.4  72 | 1.0e-114  6.3e-72  4e-37 | 5 | 39  (all) | 0.01 |  |
| **H1A123**  **gi\|449504674** | Similar to epididymal secretory protein E1/ Stathmin-3 (NPC2) | Q5ZJS7_CHICK  G3USI2_MELGA  24219 | 92.6  92.6  94 | 1.8e-38  2.0e-38  6e-30 | 5 | 61  (all) | 0.10 | C,T,Q |
| **H0Z256**  **gi\|449504751** | Similar to testican-2 (SPOCK2); domains: Kazal, EFh, TG_like 1 | R4GIB8_CHICK | 75.9 | 1.9e-67 | 2 | 7  (Z1,3b) | <0.01 |  |
| **H0ZAA2**  **gi\|449505265** | Similar to tolloid-like 2/ Metalloendopeptidase (TLL2) | F1NRI8_CHICK  G3US74_MELGA  3113/11920/28549  21127 | 93.6  96.8  66 | 0e0  0e0  0e0 | 7 | 34  (Z1,3b) | <0.01 |  |
| **gi\|449505613** | Similar to lipase member M | E1BWZ1_CHICK  G1N854_MELGA  9956/7514 | 72.0  73.5  70 | 1.7e-59  2.8e-59  1e-129 | 6 | 38  (all) | 0.01 |  |
| **H0ZEU2**  **gi\|449505657** | Similar to myoferlin (MYOF) | E1BW21_CHICK  G1N9U8_MELGA  1172/20297/23401 | 92.9  84.8  93 | 0e0  0e0  1e-121 | 2 | 7  (Z1,3b) | <0.01 | Q |
| **H0ZHE4**  **gi\|449505762** | Phosphoglycerate mutase 1 (PGAM1) | F1NHM9_CHICK  G1NBW3_MELGA  13512 | 97.5  97.5  97 | 1.8e-45  1.8e-45  8e-62 | 4 | 22  (Z2,3,3b) | 0.04 | C,T,Q |
| **gi\|449505784**  **H0ZHW4** | Similar to prominin-1-A-like (PROM2) | Q90XC6_CHICK  G1MUN6_MELGA  22275 | 69.1  66.4  76 | 4.2e-104  5.6e-99  5e-38 | 5 | 29  (Z1,3b) | <0.01 | C,T |
| **H0ZL84**  **gi\|449505943** | Similar to GDNF family receptor alpha-1 (GFRA1) | GFRA1_CHICK  G3UPH7_MELGA  7237/20607 | 94.0  93.5  82 | 1.1e-199  1.4e-196  1e-175 | 3 | 29  (all) | 0.01 |  |
| **gi\|449505989**  **H0ZL93** | Similar to pancreatic triacylglycerol lipase (PNLIPRP3-1) | F1P5K3_CHICK  G1NFD3_MELGA  7766/14539 | 49.7  49.7  44 | 4.6e-104  1.4e-102  1e-77 | 7 | 16  (Z1,2,3b) | 0.01 |  |
| **gi\|449506123**  **H0ZM61** | Similar to deleted in malignant brain tumors 1 (DMBT1) | G1NGA2_MELGA  E1BZF9_CHICK  7857 | 61.7  62.1  61 | 8.9e-119  2.0e-118  1e-125 | 10 | 119  (all) | 0.02 |  |
| **H0YXZ8**  **gi\|449506307** | Similar to fibronectin (FN1) | F1NJT3_CHICK  G1MWJ4_MELGAA0A060PIY8_COTJA | 93.6  92.6  93.6 | 0e0  0e0  0e0 | 45 | 577  (all) | 0.03 | C,T,Q |
| **gi\|449506522**  **H0Z3W6** | Insulin-like growth factor-binding protein 2 (IGFBP2) | Q802T1_CHICK  G1NGG4_MELGA  19296 | 91.0  91.5  96 | 2.8e-72  4.5e-72  4e-48 | 3 | 41  (all) | 0.03 |  |
| **H0Z3W8**  **gi\|449506910** | Insulin-like growth factor-binding protein 5 (IGFBP5) | F1ND88_CHICK  Q9DGI2_COTJA | 99.1  99.1 | 2.6e-48  1.4e-48 | 9 | 297  (all) | 1.26 | C,Q |
| **H0Z681**  **gi\|449506994** | Similar to β-galactosidase (GLB1L) | F1NW93_CHICK  H9H1U2_MELGA  6635/7550 | 81.4  91.8  53 | 0e0  1.9e-202  1e-106 | 3 | 22  (Z1,3b) | <0.01 | C,T,Q |
| **gi\|449507052**  **H0Z8Z3** | Similar to dipeptidyl peptidase 4 (DPP4) | G1NET3_MELGA  Q5ZI81_CHICK  2196/21451 | 81.4  80.1  81 | 0e0  0e0  0e0 | 13 | 80  (all) | 0.01 | T |
| **H0Z9H6**  **gi\|449507069** | Similar to cordon-bleu protein-like 1 (COBLL1) | F1NLV6_CHICK  G1NEF1_MELGA  3065/8667/19822/  29708 | 71.1  66.8  60 | 3.0e-179  5.5e-77  0e0 | 4 | 13  (Z1,3b) | <0.01 |  |
| **gi\|449508006**  **H0Z0W4** | Similar to antithrombin-III (SERPINC1) | G1MT40_MELGA  F1NLP7_CHICK  5717 | 66.7  65.8  89 | 1.6e-119  2.1e-118  1e-118 | 7 | 67  (all) | 0.02 | C,T,Q |
| **H0Z7Q9**  **gi\|449508327** | Protein CYR61 (cysteine-rich, angiogenic inducer, 61; CYR61; IPI00578074) | CYR61_CHICK  G1N0Q2_MELGA  9187 | 95.4  95.3  87 | 2.2e-135  7.9e-133  1e-166 | 5 | 76  (all) | 0.01 | C |
| **H0Z858**  **gi\|449508450** | Similar to di-N-acetylchitobiase (CTBS), shares 2 peptides with gi\|224057612 (Vitellogenin-2-like) | F1NRM4_CHICK  G1NAD9_MELGA  9593/8527 | 89.5  89.9  77 | 2.2e-135  2.2e-135  1e-146 | 8 | 185  (all) | 0.08 | C,T,Q |
| **gi\|449508478**  **H0Z9P8** | Similar leucine-rich repeat-containing protein 19 (LRRC19) | F1NGV7_CHICK  G1NBL5_MELGA  11977 | 75.9  74.1  60 | 3.7e-49  6.4e-47  6e-96 | 2 | 27  (all) | 0.01 | C |
| **H0ZAM5**  **gi\|449508498** | Receptor-type tyrosine-protein phosphatase F (PTPRF); shares 1 peptide with H0YR81 (PTPRS) | F1N897_CHICK  G1NBY3_MELGA  4319/6084/7499 | 98.3  94.9  98 | 0e0  0e0  0e0 | 18 | 113  (all) | <0.01 | C,T,Q |
| **gi\|449508571**  **H0ZCW3** | Similar to tetraspanin (TSPAN1) | F1NBL2_CHICK  G1NDT9_MELGA  16378 | 75.8  74.2  55 | 2.1e-67  2.1e-67  2e-66 | 3 | 43  (all) | 0.33 | (C) |
| **H1A388**  **gi\|449509160** | Similar to cyclic AMP-dependent transcription factor ATF-6 α (ATF6) | F1NXG4_CHICK  G1N0W8_MELGA  14909/23971/29367 | 77.3  80.5  71 | 1.4e-161  1.6e-159  1e-60 | 3 | 5  (Z1,3b) | <0.01 |  |
| **H1A3Q8**  **gi\|449509191** | Similar to regenerating islet-derived protein 4 (REG4) | G1MZE6_MELGA  E1BZV4_CHICK  8715 | 60.0  58.3  57% | 9.8e-39  1.4e-38  2e-11 | 2 | 10  (Z1,3b) | <0.01 | C,T,Q |
| **H1A439**  **gi\|449509315** | Similar to sulfhydryl oxidase 1 (QSOX1) | QSOX1_CHICK  G3UTX4_MELGA  2299/5467/13235 | 74.2  72.6  72 | 3.9e-186  1.0e-144  0e0 | 23 | 991  (Z1,2,3, 3b) | 0.56 | C,T,Q |
| **H0Z500**  **gi\|449509472** | Similar to glypican-1 (GPC1) | F1P150_CHICK  Q6QA54_MELGA  3589/20854 | 92.6  92.2  82 | 4.5e-209  3.1e-208  0e0 | 5 | 21  (Z1,3b) | <0.01 | C,T,Q |
| **H0Z885**  **gi\|449509524** | Similar to ovotransferrin (LTF) | G1MVV5_MELGA  Q4ADJ7_CHICK  2596/24968 | 77.3  75.8  74 | 0e0  0e0  0e0 | 43 | 1501  (all) | 0.69 | C,T,Q |
| **gi\|449509755** | Procollagen-lysine,2-oxoglutarate 5-dioxygenase 2; shares 25 peptides with H0ZER1 | F1NXB0_CHICK  G1N4C8_MELGA  14084/22196 | 94.7  87.5  75 | 0e0  5.0e-197  2e-93 | 26 | 386  all) | 0.05* | C,T |
| **H0ZER1** | Procollagen-lysine,2-oxoglutarate 5-dioxygenase 2 (PLOD2); shares 25 peptides with gi\|449509755 | G1N4C8_MELGA  F1NXB0_CHICK  14084/22196 | 93.7  91.6  82 | 0e0  2.3e-200  3e-97 | 1 | 45  (all) |  |  |
| **gi\|449509821**  **H0ZJJ6** | Similar to α-2-HS-glycoprotein (AHSG) | G1NAQ8_MELGA  E1BZE1_CHICK  12006 | 75.1  74.0  66 | 3.2e-111  2.7e-108  8e-68 | 4 | 118  (all) | 0.20 | C,T |
| **H0ZFP0**  **gi\|449509897** | Similar to leucine-rich repeat-containing protein 15 (LRRC15) | F1N822_CHICK  4010 | 79.2  77 | 1.2e-151  0e0 | 2 | 5  (Z1,2) | <0.01 |  |
| **H0ZG88**  **gi\|449509913** | Similar to interleukin-1 receptor accessory protein (ILRAP) | G1N684_MELGA  E1C117_CHICK  6206/5403 | 85.5  87.1  87 | 0e0  8.3e-123  0e0 | 2 | 6  (Z2,3,3b) | <0.01 | C,T,Q |
| **H0ZJD2**  **gi\|449509966** | Similar to chordin (CHRD-1) | G1NBK1_MELGA  R4GI42_CHICK | 89.7  86.7 | 3.3e-168  2.2e-162 | 8 | 32  (Z1,3,3b) | <0.01 | C,T |
| **gi\|449509974**  **H0ZJK7** | Similar to kininogen-1(KNG1) | E1BX43_CHICK | 74.6 | 1.5e-76 | 13 | 239  (all) | 0.08 |  |
| **gi\|449510014**  **H0ZLC8** | Similar to Golgi integral membrane protein 4 (GOLIM4) | G1NDC2_MELGA  E1BXE0_CHICK  6052/6973 | 81.3  76.7  71 | 3.1e-37  1.0e-30  1e-162 | 12 | 48  (Z1,3b) | 0.01 | C,T |
| **H0ZLS5**  **gi\|449510033** | Similar to ovocalyxin-32 (RARRES1) | D3KYT5_CHICK | 56.3 | 8.5e-47 | 7 | 198  (all) | 0.40 | C |
| **H0ZZA0**  **gi\|449510108** | ATP synthase subunit β (ATP5B) | H9L340_CHICK  H9H062_MELGA | 98.8  99.5 | 3.6e-92  5.9e-69 | 3 | 3  (Z1,3b) | <0.01 | T |
| **H0ZVQ5**  **gi\|449510382** | Similar to choline transporter-like 2 (SLC44A2) |  |  |  | 8 | 50  (Z1,3,3b) | 0.01 |  |
| **gi\|449510589**  **H0ZY95** | Similar to peflin (PEF1) | H9KZ63_CHICK  G3UT72_MELGA  8164 | 80.0  79.9  83 | 4.6e-35  9.1e-28  2e-83 | 2 | 9  (Z1,3b) | 0.01 | C,Q |
| **gi\|449510911**  **H1A258** | Similar to glucosylceramidase | R4GIA6_CHICK | 83.2 | 7.1e-68 | 8 | 46  (all) | 0.01 |  |
| **H1A041**  **gi\|449511711** | Similar to lamin-A (LMNA) | LMNA_CHICK  G1N7C3_MELGA  8553/26573 | 82.1  51.2  60 | 4.4e-105  1.4e-55  1e-46 | 3 | 4  (Z1,2,3b) | <0.01 | C |
| **gi\|449512423**  **H0ZX61** | Similar to CD276 | G1MTD5_MELGA  F1NI56_CHICK  11598/10046 | 82.4  82.1  84 | 9.4e-107  4.9e-105  2e-51 | 4 | 46  (all) | 0.04 |  |
| **gi\|449512477**  **H1A5U7** | Similar to fibroblast growth factor-binding protein 1 (FGFBP1) | F1NG86_CHICK  G1NJ28_MELGA  13722 | 69.7  69.7  66 | 7.5e-69  4.0e-69  5e-88 | 4 | 93  (all) | 0.05 | T |
| **gi\|449513692**  **H0ZXB7** | Similar to β-1,4-galactosyltransferase 3 |  |  |  | 2 | 13  (Z1,3b) | 0.01 |  |
| **H0YQQ3**  **gi\|449513868** | Similar to cathepsin L1/L2 (CTSL1) | F1NYJ1_CHICK  G1N4I7_MELGA  9862 | 96.6  94.8  84 | 5.0e-146  2.9e-84  1e-162 | 4 | 160  (all) | 0.18 | C,T,Q |
| **H0YRA6**  **gi\|449513891** | Annexin (ANXA1) | F1N9S7_CHICK  G1N2K9_MELGA  23618 | 90.6  66.8  57 | 6.3e-118  2.5e-68  2e-27 | 9 | 54  (Z1,2,3b) | 0.02 | C,T,Q |
| **gi\|449513913**  **H0YR65** | Similar to proprotein convertase subtilisin/kexin type 5; shares 1 peptide with H0ZFA7 (PCSK6) | F1NU61_CHICK  G3UV24_MELGA  12312/12892/17888/  28313/30344 | 81.9  72.1  72 | 0e0  0e0  1e-114 | 4 | 18  (Z1,2,3b) | <0.01 |  |
| **gi\|449513925**  **H0YRN6** | Similar to peptidyl-glycine α-amidating monooxygenase (PAM) | F1NQN1_CHICK  G1N537_MELGA  13480/14851 | 87.6  67.8  73 | 0e0  1.0e-162  7e-51 | 23 | 266  (all) | 0.04 |  |
| **H0YU57**  **gi\|449514211** | Similar to transitional endoplasmic reticulum ATPase (VCP) | Q5ZMU9_CHICK  G1MUF1_MELGA  1913/19066 | 96.8  90.2  96 | 0e0  9.7e-209  0e0 | 9 | 33  (Z1,2,3b) | <0.01 | C,T,Q |
| **gi\|449514273**  **H0YV99** | Similar to complement component 9 (C9); domains: TSP1, LDLRA, MACPF |  |  |  | 6 | 20  (Z1,3b) | <0.01 |  |
| **H0Z063**  **gi\|449514410** | Similar to arylsulfatase B (ARSB) | F1P099_CHICK  27451 | 89.0  86 | 5.0e-210  1e-43 | 5 | 29  (all) | <0.01 | Q |
| **H0ZHM0** | Similar to UDP-GlcNAc:βGal beta-1,3-N-acetylglucosaminyltransferase 7 (B3GNT7); shares 11 peptides with gi\|350534788/B5FXB7 | F1NU54_CHICK  G1N7V1_MELGA  6163/8823 | 93.7  94.3  93 | 2.9e-140  5.9e-140  0e0 | 2 | 20  (Z1,3b) | 0.07* | C,T,Q |
| **B5FXB7**  **gi\|350534788** | Putative β-13-N-acetylglucosaminyltransferase (B3GNT7); shares 11 peptides with H0ZHM0 | F1NU54_CHICK  G1N7V1_MELGA  6163/8823 | 86.8  87.5  86 | 3.1e-149  2.5e-146  0e0 | 14 | 272  (all) |  |  |
| **H0YPM9**  **gi\|449489337** | Uncharacterized protein (TMPRSS4); domains: SRCR-like, Peptidase S1A | F1N9M8_CHICK  G1MXR5_MELGA  11515 | 79.1  77.9  64 | 4.1e-115  6.4e-114  2e-53 | 3 | 22  (all) | <0.01 | C,T,Q |
| **H0YQL9**  **gi\|224067572** | Uncharacterized protein (OVM)/similar to ovomucoid; shares 5 peptides with H1A0C8 | G1MZY1_MELGA  IOVO_CHICK  IOVO_COTJA | 70.1  64.8  69.4 | 1.4e-65  1.9e-60  1.0e-57 | 11 | 382  (all) | 1.73* | C,T,Q |
| **gi\|449512555**  **H1A0C8** | Similar to ovomucoid; shares 5 peptides with H0YQL9 | IOVO_MELGA  IOVO_CHICK  IOVO_COTJA | 67.6  62.4  64.1 | 8.8e-57  5.5e-52  5.1e-50 | 2 | 74  (all) |  |  |
| **H0YRU7**  **gi\|224089166** | α-mannosidase (MAN2A1) | F1NKR1_CHICK  G3UQ40_MELGA  6738/25812/22889 | 90.7  69.8  57 | 0e0  0e0  1e-82 | 22 | 162  (all) | 0.02 | C,T |
| **H0YS14**  **gi\|449490180** | Similar to complement receptor type 2/ Complement regulatory soluble protein (CR2/CRES) | G1MYD5_MELGA  F1P2M6_CHICK  2511/741/7089 | 54.3  54.0  53 | 1.7e-181  7.3e-180  0e0 | 7 | 88  (all) | 0.02 | (C) |
| **H0YSA5**  **gi\|449488884** | Similar to chloride intracellular channel protein 4 (CLIC4);shares 6 peptides with H0ZUQ9 | F1NYZ7_CHICK  G1MT68_MELGA  11834 | 93.6  95.3  96 | 2.5e-93  1.8e-93  1e-140 | 9 | 117  (all) | 0.04* | C,T,Q |
| **gi\|224178762**  **H0ZUQ9** | Similar to chloride intracellular channel protein (CLIC4); shares 5 peptides with H0YSA5 | G1MT68_MELGA  FR1NYZ7_CHICK  11834 | 96.9  96.4  96 | 2.5e-78  4.0e-78  1e-107 | 1 | 30  (Z1,2,3b) |  |  |
| **H0YSJ4**  **gi\|350539137** | Similar to palmitoyl-protein thioesterase 1 (PPT1) | F1NYK1_CHICK  G1N1R1_MELGA  15308/24474 | 86.8  82.3  61 | 1.5e-113  6.8e-98  2e-85 | 7 | 55  (all) | 0.01 | C,T,Q |
| **H0YUD9** | Similar to avidin-related protein | R4GLN1_CHICK  G1MSZ2_MELGA  24728 | 79.6  53.5  49 | 5.2e-41  2.0e-20  2e-20 | 5 | 50  (all) | 0.04 | C,T,Q |
| **H0YVU1**  **gi\|449495663** | Actin-related protein 2 (ACTR2) | G3UV97_MELGA  F1NRM5_CHICK  17821/20606/26054 | 99.7  99.7  100 | 5.0e-154  5.0e.154  6e-80 | 2 | 8  (Z1,3b) | <0.01 | T |
| **H0YWI3**  **gi\|350537445** | Similar to integral membrane protein 2A (ITM2A) | F1P4X6_CHICK  G1MSA8_MELGA  19984/21988 | 94.0  95.1  95 | 2.0e-109  1.3e-98  2e-75 | 3 | 12  (Z2,3,3b) | 0.01 | C,T,Q |
| **H0YXM0** | Similar to IGF-binding protein 7 (IGFBP7) | F1NVP4_CHICK  G1N8M9_MELGA  18344 | 93.8  89.3  95 | 7.5e-81  1.2e-47  7e-64 | 3 | 20  (all) | 0.01 | C,T,Q |
| **H0YXZ3** | Similar to LOC419409/Golgi phosphoprotein 4 | E1BU50_CHICK  G1MUR4_MELGA  10834/10704/11207 | 92.7  80.9  71 | 4.0e-45  4.1e-40  4e-60 | 5 | 32  (all) | 0.01 | C,T,Q |
| **H0YYU5** | Puromycin-sensitive aminopeptidase (NPEPPS) | G1MUX4_MELGA  F1P456_CHICK  1927/5771 | 95.3  95.3  86 | 0e0  0e0  0e0 | 18 | 82  (all) | 0.01 | T |
| **H0Z125** | β-actin (Q90WZ7); shares 14 peptides with H0Z0Q3 and 10 with H0ZNH2 | Q58J72_MELGA  ACTB_CHICK  7902 | 97.8  97.8  87 | 1.5e-130  1.5e-130  1e-160 | 2 | 17  (Z1,2,3) | 0.01 | C,Q |
| **H0Z157**  **gi\|449504732** | Similar to proactivator polypeptide (PSAP-1) | E1BSP1_CHICK  G1MZV1_MELGA  1756/2306 | 89.1  76.3  83 | 2.6e-194  9.9e-122  0e0 | 19 | 483  (all) | 0.20 | C,T,Q |
| **H0Z1I2**  **gi\|224045479** | Similar to prostatic acid phosphatase (ACPP) | F1NT31_CHICK  G1NG25_MELGA  26951 | 85.2  87.4  89 | 5.8e-172  4.9e-143  1e-37 | 7 | 161  (Z1,2,3, 3b) | 0.06 | C |
| **H0Z2G9**  **gi\|449501641** | Similar to leucine zipper protein 2 (LUZP2) | G1MV75_MELGA  F1P144_CHICK | 85.5  84.0 | 1.4e-48  4.2e-47 | 7 | 110  (Z1,2,3, 3b) | 0.06 | C |
| **H0Z423**  **gi\|449479002** | Netrin-1 (NTN1); shares 1 peptide with H0YYI9 (Netrin-3) | NET1_CHICK  H9H0I5_MELGA  3495/10003 | 97.0  98.0  97 | 1.2e-199  3.0e-124  0e0 | 1 | 94  (all) | 0.09 |  |
| **H0Z777**  **gi\|449500526** | Polypeptide N-acetylgalactosaminyltransferase (GALNT7) | G1N4S2_MELGA  F1NPU9_CHICK  19345/11549/15791 | 96.2  95.9  90 | 0e0  0e0  0e01e-76 | 4 | 23  (all) | 0.01 |  |
| **H0Z7E2**  **gi\|449486391** | Similar to bactericidal permeability-increasing protein (BPI) | F1NXX9_CHICK  G1N743_MELGA  17946 | 70.4  70.9  65 | 9.4e-140  1.2e-138  7e-62 | 13 | 144  (all) | 0.05 | T |
| **H0Z7I9**  **gi\|449486399** | BPIL1/Tenp-like | I0J172_CHICK  T2MK81_MELGA  - | 61.7  63.0 | 8.3e-105  4.7e-86 | 19 | 1180  (all) | 3.72 | C,T |
| **H0Z8L2**  **gi\|224073876** | Similar to gelsolin (GSN) | G1N751_MELGA  F1NKF3_CHICK  2080/3462/4097 | 90.7  90.6  90 | 0e0  0e0  0e0 | 22 | 344  (all) | 0.07 | C,T,Q |
| **H0Z9A2**  **gi\|197127412** | T-complex protein 1 subunit beta (CCT2) | Q5F424_CHICK  G1NPD9_MELGA  15095/19006 | 97.8  97.9  97 | 2.6e-203  5.4e-147  2e-94 | 3 | 4  (Z1,3b) | <0.01 |  |
| **H0ZCH4**  **gi\|449486461** | Similar to cathepsin Z (CTSZ); shares 2 peptides with H1A1L3 | E1C4M3_CHICK  G1N9B0_MELGA  15110 | 93.8  94.3  93 | 4.7e-117  9.3e-103  1e-115 | 4 | 49  (all) | 0.02* | C,T,Q |
| **H1A1L3**  **gi\|224078756** | Similar to cathepsin Z; shares 2 peptides with H0ZCH4 | G1N9B0_MELGA  E1C4M3_CHICK  15110 | 94.3  94.3  92 | 4.8e-62  7.8e-62  3e-80 | 1 | 1  (Z1) |  | C |
| **H0ZEP4** | Heparin cofactor II (SERPIND1) | O73840_CHICK  G1MQH6_MELGA  10000/9359 | 89.4  89.4  90 | 1.7e-179  6.1e179  1e-140 | 2 | 4  (Z1,3b) | <0.01 | C,T,Q |
| **H0ZGJ3**  **gi\|449476462** | Similar to transmembrane channel-like 7 (TMC7) | F1NIM0_CHICK  G1N7C5_MELGA  2361 | 78.4  67.6  78 | 0e0  2.0e-183  0e0 | 4 | 7  (Z1,3b) | <0.01 |  |
| **H0ZH52**  **gi\|449500994** | Slit homolog 2 protein (SLIT2); shares 3 peptides with gi\|224068202 (SLIT3) | F1NR89_CHICK  G1NIV1_MELGA  13685/23949/16772/  20544 | 98.0  97.9  97 | 0e0  0e0  1e-122 | 48 | 1025  (all) | 0.21 | C,T,Q |
| **H0ZHZ7** | Similar to 14-3-3 protein eta (YWHAH); shares 3 peptides with other 14-3-3 proteins | G1NR71_MELGA  Q5ZKJ3_CHICK  13907 | 100.0  100.0  100 | 1.7e-88  1.9e-88  1e-122 | 1 | 2  (Z3b) | <0.01 |  |
| **H0ZIC9** | Similar to calcium and integrin-binding protein 1 (CIB1) | F1P103_CHICK  G1NDJ9_MELGA  18780 | 88.8  85.9  82 | 2.7e-56  9.3e-42  2e-57 | 3 | 34  (all) | 0.03 | C,T,Q |
| **H0ZS62** | Similar to ovostatin (A2M-1) | F1NU63_CHICK  G3URW8_MELGA  389/455/5368/  14911/18662 | 78.6  78.4  78 | 0e0  0e0  0e0 | 79 | 2982  (all) | 1.20 | C,Q |
| **H0ZS79**  **gi\|350537035** | Similar to α-2-macroglobulin (A2M-2); shares 13 peptides with H0ZS92 (A2M-3) | E1NF29_CHICK  G1NMH1_MELGA  9582/25258 | 78.8  78.3  78 | 0e0  0e0  1e-111 | 26 | 267  (all) | 0.02 | C,Q |
| **H0ZSA0** | Similar to α-2-macroglobulin-like 1; shares 3 peptides with gi\|449485115 | F1NTK2_CHICK  G1NME9_MELGA  17778 | 54.4  63.0  72 | 1.7e-177  8.7e-177  5e-60 | 16 | 275  (all) | 0.11 | C,Q |
| **H0ZSL3**  **gi\|449498236** | Similar to G-protein coupled receptor 116 (GPR116) | F1NEZ6_CHICK  G1NN99_MELGA  2081/3063 | 61.2  60.4  63 | 0e0  0e0  0e0 | 5 | 23  (Z1,3,3b) | <0.01 | T |
| **H0ZST9**  **gi\|197129222** | Similar to cathepsin B (CTSB) | G1NN37_MELGA  F1N9D8_CHICK  8924/17826 | 94.4  92.9  72 | 1.0e-150  1.4e-148  1e-156 | 12 | 167  (all) | 0.08 | C,T,Q |
| **H0ZT35** | Similar to sodium/potassium-transporting ATPase subunit α-1 (ATP1A1) | F1NSY1_CHICK  G1NNB4_MELGA  1069/1484/8468/  16229 | 96.9  96.1  96 | 0e0  0e0  0e0 | 5 | 11  (Z1,3b) | <0.01 | C,T,Q |
| **H0ZYE3** | Similar to dynamin-2 (DNM2); shares 2 peptides with H0Z046 (DNM1) | E1BXY4_CHICK  G1MY24_MELGA | 64.7  64.7 | 2.6e-144  2.4e-146 | 4 | 11  (Z1,2,3b) | <0.01 |  |
| **H0ZZX0** | Uncharacterized protein (LSR)/ILDR2; domain: IG_like | F1NTX7_CHICK  G1NNP5_MELGA  20163 | 47.4  47.4  54 | 3.5e-10  1.3e-10  1e-18 | 2 | 10  (Z2,3b) | 0.01 |  |
| **H1A1I4** | Similar to sphingomyelin phosphodiesterase (SMPD1) | R4GHJ3_CHICK  14179 | 82.2  80 | 1.1e-83  7e-87 | 5 | 116  (all) | 0.10 | C,Q |
|  |  |  |  |  |  |  |  |  |

*, iBAQ % for combined sequences. C, chicken [30-36,38]; T, turkey [52] ; Q, quail [53]. **^1^**, FASTA against UniProt protein sequence database. **^2^**, BLASTP as provided by BioEdit Sequence Alignment Editor under Accessory Aplications, Local Blast, against the NODAI Coturnix predicted gene database.
